# Synthesis and Anticancer Activity Evaluation of New 5-((5-Nitrofuran-2-yl)allylidene)-2-thioxo-4-thiazolidinones

**DOI:** 10.3390/ph18111598

**Published:** 2025-10-22

**Authors:** Magdalena Podolak, Volodymyr Horishny, Rostyslav Dudchak, Agnieszka Gornowicz, Robert Czarnomysy, Dmytro Mural, Serhii Holota, Krzysztof Bielawski, Roman Lesyk, Anna Bielawska

**Affiliations:** 1 Department of Biotechnology, Faculty of Pharmacy, Medical University of Bialystok, Jana Kilinskiego 1, 15-089 Bialstok, Poland; agnieszka.gornowicz@umb.edu.pl (A.G.); anna.bielawska@umb.edu.pl (A.B.); 2 Department of Pharmaceutical, Organic and Bioorganic Chemistry, Danylo Halytsky Lviv National Medical University, Pekarska 69, 79010 Lviv, Ukraine; vgor58@ukr.net (V.H.); golota_serg@yahoo.com (S.H.); 3 Department of Synthesis and Technology of Drugs, Faculty of Pharmacy, Medical University of Bialystok, Jana Kilińskiego 1, 15-089 Bialystok, Poland; rostyslav.dudchak@sd.umb.edu.pl (R.D.); robert.czarnomysy@umb.edu.pl (R.C.); krzysztof.bielawski@umb.edu.pl (K.B.); 4 Department of Pharmaceutical Chemistry, National University of Pharmacy, Valentynivska St., 4, 61168 Kharkiv, Ukraine; dmural003@gmail.com; 5 Molecular Design Center, Danylo Halytsky Lviv National Medical University, Pekarska 69, 79010 Lviv, Ukraine; 6 Department of Biotechnology and Cell Biology, Medical College, University of Information Technology and Management in Rzeszow, Sucharskiego 2, 35-225 Rzeszow, Poland

**Keywords:** 4-thiazolidinone, hybrid molecules, breast cancer, intrinsic pathway, cytotoxic activity

## Abstract

**Background/Objectives**: Cancer persists as a leading concern in the current medical field. As such, scientists are continuously researching new compounds with anticancer potential. In this study, we explored fifteen new 4-thiazolidinone derivatives as potential anticancer compounds. 4-Thiazolidinones are a well-established group of active structures, most commonly applied for the treatment of Parkinson’s disease and diabetic neuropathy. However, they are actively researched as potential anticancer agents. A number of derivatives have qualified for Phase II and III clinical trials as antitumor agents. **Methods**: MTT cytotoxicity assay was applied to identify the most active compounds. Three out of the fifteen tested structures displayed a significant inhibitory effect on the MCF-7 and MDA-MB-231 cell lines. To further investigate the influence of compounds on breast cancer cells, we analyzed their capability to induce apoptosis using flow cytometry assessment with Annexin V and propidium iodide dyes. Next, flow cytometry analysis of JC-1 dye was utilized to research their capability to affect mitochondrial membrane. Afterwards, concentrations of important proapoptotic proteins such as Bax and cytochrome C were assessed with a highly sensitive ELISA method. **Results**: Further analysis with a fluorescent microscope displayed that novel compounds significantly increase the generation of reactive oxygen species. **Conclusions**: The results represented in this article displayed that the most active compounds positively affected the activation of the intrinsic apoptotic pathway in the tested breast cancer cells.

## 1. Introduction

**Figure 1:**
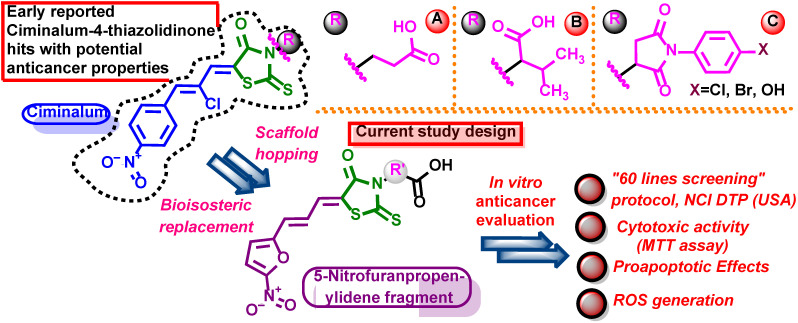
Structures of Ciminalum-4-thiazolidinone hybrid molecules as antitumor agents and design of the present study.

Ciminalum-4-thiazolidinone hybrid molecules are intensely researched in the field of medicinal chemistry as potential antitumor agents. Such compounds are characterized by their potent anticancer effects, targeted mechanisms of activity, and satisfactory cytotoxicity properties towards normal human cells [1]. The special interest presents Ciminalum-4-thiazolidinone hybrids with the carboxylic group or its functional derivatives in position three of the 4-thiazolidinone ring (Figure 1). A library of Ciminalum-4-thiazolidinone hybrid molecules bearing a carboxylic group and its derivatives was designed, synthesized, and evaluated for its anticancer potential following the “60 lines screening” protocol (NCI DTP, USA) by Buzun et al. [2]. Among the studied compounds, a derivative with propanoic acid residue at N-3 (Figure 1, substructure A) was identified with a total GI_50_ value of 1.57 µM and GI_50_ concentration range of <0.01–0.02 μM toward the MOLT-4, SR (Leukemia), SW-620 (Colon cancer), SF-539 (CNS cancer), and SK-MEL-5 (Melanoma) cell lines. Afterwards, Szlachcikowska et al. expanded the knowledge of the anticancer effects of the mentioned hybrid (Figure 1, substructure A) and showcased its effects on a number of molecular targets, such as NF-κB, β-tubulin, and β-actin. The tested structure reduced NF-κB gene expression and protein level in BJ and A549 cell lines. Furthermore, it also reduced the expressions of PPARγ and NRF2 in the BJ cell line [3]. In a continuation of their studies, Buzun et al. reported on the other hybrid with a 3-methylbutanoic acid fragment (Figure 1, substructure B) which displayed a potent cytotoxic activity towards MCF-7 and MDA-MB-231 breast cancer cell lines, with IC_50_ equal to 5.02 µM and 15.24 µM, respectively [4]. Moreover, the studied compounds (Figure 1, substructure B) also induced apoptosis, decreased mitochondrial membrane potential, increased caspase-9 and caspase-8 concentrations, and decreased Topoisomerase II concentration.

Another series of ciminalum-4-thiazolidinone hybrid molecules, bearing phenylsuccinimide moieties (Figure 1, substructures C) were tested in according with the “60 lines screening” protocol (NCI DTP (USA)). Among the tested compounds there were structures that have been identified as potential antitumor agents possessing submicromolar cytotoxic activity towards leukemia, colon cancer, CNS, and ovarian cancer cell lines with a high selectivity index [5]. Derivatives (Figure 1, substructures C) were also found to be potent towards human tongue squamous cell carcinoma cells of the SCC-15 line with IC_50_ values in the range of 6.72–39.85 µM [6]. In-depth studies showcased that such hybrid molecules (Figure 1, substructures C) possessed significant toxicity towards a number of breast cancer cell lines: MDA-MB-231, MCF-7, T-47D, HCC1954 cells, and murine breast cancer 4T1 cells with the IC_50_ values ranging from 1.37 to 21.85 µM [7]. All of the mentioned hybrids (Figure 1, substructures A–C) did not display potent cytotoxic effects towards normal human keratinocytes of the HaCaT line and mitogen-activated lymphocytes of the peripheral blood of healthy human donors. Mentioned hybrids were characterized with high selectivity indexes [2,3,4,5,6,7].

Previously described 5-nitrofuran derivatives also displayed considerable anticancer effects [8]. Well-known FDA-approved antibiotic nitrofuroxazide promoted apoptosis by inhibiting the STAT3 protein in myeloma and colon cancer cells [9,10]. Another widely utilized 5-nitrofuran antibiotic furazolidone promoted programmed cell death in acute myeloid leukemia [11]. Interestingly enough, synthesized nitrofurantoin derivatives showcased potent anticancer activity towards colon cancer (Caco-2), hepatic cancer (HepG-2), cervical cancer (HeLa), and breast cancer (MCF-7) by promoting oxidative stress and inducing DNA damage, inherently leading to apoptosis induction [12].

Moreover, 5-nitrofuranpropenylidene moiety is a crucial structure fragment of Eeyarestatins—small molecules that were initially identified as inhibitors of protein dislocation from the endoplasmic reticulum into the cytosol [13], however, nowadays are often studied as anticancer agents against different tumor types acting via multiple mechanisms [14,15,16,17]. Additionally, Guillon et al. [18] used a 5-nitrofuranpropenylidene fragment for the design of the 1,2,4-triazole, with bearing imines acting as dual iNOS and tumor cell growth inhibitors.

Considering the antitumor potential of the described hybrids and the possibilities of applying scaffold hopping and bioisosteric replacement methodologies, in the present paper, we report a synthesis of 4-thiazolidinone hybrids with a 5-nitrofuranpropenylidene moiety and an evaluation of their anticancer properties.

## 2. Results

### 2.1. Chemistry

**Scheme 1:**
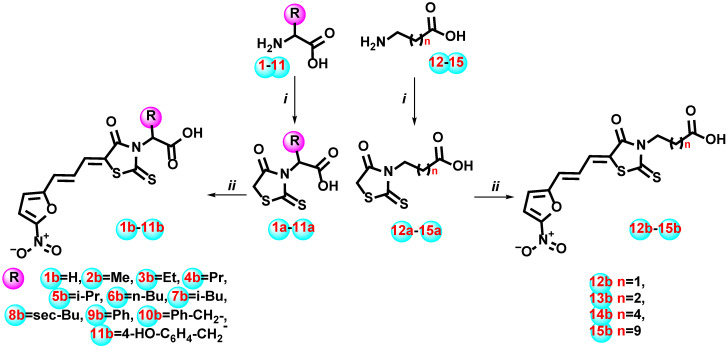
Synthesis of rhodanine carboxylic acids with nitrofuranpropenylidene fragment **1b**–**15b**. Reagents and conditions: (i) (a) appropriate amino acid **1**–**15** (10 mmol), CS_2_ (11 mmol), KOH water solution (30 mmol), r.t., stirring 1 h; (b) ClCH_2_COONa (11 mmol), r.t., stirring 12 h; (c) 6 N HCl solution (5 mL), reflux 1 h; (ii) **1a**–**15a** (10 mmol), 3-(5-nitrofuran-2-yl)acrylaldehyde (12 mmol), AcONa (10 mmol), AcOH (50 mL), reflux, 4–5 h, 80–88%.

The synthetic design of the reported study was based on diversifying the substituent at the N-3 atom of the 4-thiazolidinone ring with carboxylic group-bearing moieties. The set of commercially available aminoacids **1**–**15** was used as starting building blocks. Initially, aminoacids **1**–**15** were subjected to a one-pot reaction of dithiocarbamate generation with CS_2_ in the basic medium and cyclization by sodium chloroacetate, with treatment by concentrated HCl yielding to derivatives **1a**–**15a,** as presented in Scheme 1. Then, obtained rhodanine-3-carboxylic acids **1a**–**15a** were transformed to the corresponding 5-ylidene derivatives **1b**–**15b** in the Knoevenagel condensation, with 3-(5-nitrofuran-2-yl)acrylaldehyde using as a catalyst an anhydrous sodium acetate in the glacial acetic acid medium with yields of 80–88%.

The structures of the synthesized derivatives **15a**, **1b**–**15b** were confirmed by ^1^H, ^13^C NMR, LC-MS, and IR spectra (copies of the corresponding spectra are presented in the Supplementary Materials Figures S1–S63). In the ^1^H NMR spectra of the synthesized 5-ylidene derivatives **1b**–**15b** protons of propenylidene moiety resonate as a doublet at 7.56–7.66 ppm, a doublet of doublets at 7.09–7.15 ppm, and one proton signal is overlapped with furan ring proton signal giving a multiplet at 7.34–7.44 ppm. Coupling constant values for these protons in the range of ~11–16 Hz confirm that synthesized compounds **1b–15b** exist with the stereochemistry of the propenylidene moiety as depicted in Scheme 1 and are in correspondence with early values reported for related structures [19,20,21]. Additionally, one furan ring proton resonates as a doublet at 7.78–7.80 ppm with a relevant coupling constant (*J* = 3.9 Hz). The aliphatic and aromatic protons of amino acid moieties resonate in the corresponding regions with relevant spin–spin coupling constants. In the ^13^C NMR spectra of rhodanine derivatives, signals of C=O and C=S groups of the core heterocycle and carboxylic group were characteristic and appeared at 163.0–169.0 and 191.9–193.0 ppm, respectively. The aliphatic and aromatic carbons of amino acid moieties give singlet signals in the relevant regions. In the IR spectra of the synthesized derivatives **1b**–**15b** the absorption bands at ~1700–1730 and ~1680–1700 cm^−1^ are assigned to the C=O stretching vibrations, respectively. The absorption bands at ~1570–1580 and 1559 cm^−1^ appeared in the IR spectra of **1b**–**15b** and corresponded to the C=C vibrations. Absorption bands at ~1507–1515 and ~1312–1346 cm^−1^ are assigned to N-O stretching vibrations. The molecular ion peaks observed at the *m/z* values in the positive ionization mode in the mass spectra confirmed the formation of the compounds **15a**, **1b**–**15b**.

### 2.2. Biology

#### 2.2.1. In Vitro Evaluation of the Anticancer Activity According to the DTP NCI Protocol

Synthesized compounds **4b**–**6b**, **9b,** and **12b**–**14b** were tested on a panel of 60 cancer cell lines at one dose assay (10^−5^ M) within the Developmental Therapeutic Program (DTP) by the National Cancer Institute (NCI, Bethesda, MD, USA). Primary anticancer assays were performed according to the NCI protocol, as described before [22,23,24]. The percentage of growth (GP) was evaluated spectrophotometrically versus controls not treated with test agents after 48 h of exposure and using SRB protein assay to estimate cell viability or growth. The obtained results provided important insights regarding the anticancer activity of the new structures and are presented in (Supplementary Materials, Tables S1 and S2). Based on the screening, three compounds were studied in more detail for specific activity against breast cancer cells.

#### 2.2.2. Cytotoxic Activity

The NCI-60 screening protocol utilizes the sulforhodamine B (SRB) assay following 48 h of compound exposure. However, due to the pronounced anticancer potency of the tested compounds, an additional evaluation was conducted after 24 h of treatment. The MTT assay was performed on eleven cancer cell lines: human breast cancer (MDA-MB-231, MCF-7, HCC 1954), human gastric cancer (AGS), human colorectal adenocarcinoma (HT-29, DLD-1, HCT 116), human neuroblastoma (SH SY5Y), and human leukemia (JURKAT, RMPI 8866, K-562) cell lines. The study was conducted in triplicate at the following seven concentrations: 0.62, 1.25, 2.5, 5, 10, 20, and 40 μM. Compound **14b** displayed the most potent anticancer activity on all cell lines among the tested compounds.

**Figure 2:**
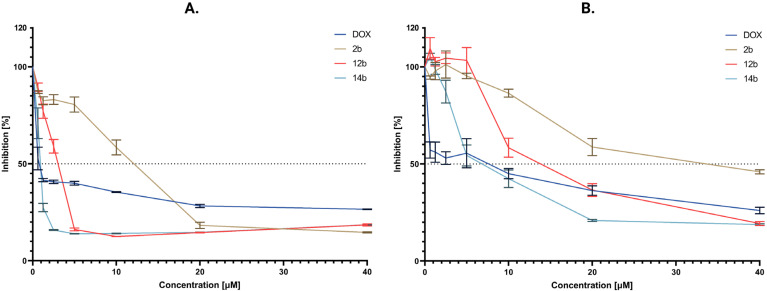
Dose–response curves representing the results of the MTT assay for the cytotoxicity of cancer cells of the most active compounds **2b**, **12b**, and **14b**. (**A**) MCF-7 line (**B**) MDA-MB-231 line. The dotted line represents the value of 50% cell inhibition in relation to the concentration of the tested compounds. The number of repetitions for each compound and for each line is 3.

The results demonstrated significant inhibition of cell proliferation even at this earlier time point, indicating the potency of the cytotoxic activity of novel compounds (Table 1). Considerably important results were noted for the breast cancer MDA-MB-231 cell line with 6.61 μM after 24 h of treatment. The above-mentioned cell line is well-known for its lack of estrogen, progesterone receptors, and HER2 amplification. Because of those factors, it is comparatively resistant towards numerous chemotherapeutic methods and drugs [25,26]. Additionally, the anticancer activity of the compound **14b** on the MCF-7 breast cancer cell line was even more impressive with 0.85 μM after 24 h of treatment. Displayed activity was significant in comparison to other thiazolidinone and rhodanine derivatives. For example, the most potent thiazolidinone structure displayed IC_50_ 1equal to 7.6 μM and 8.4 μM for MDA-MB-231 and MCF-7, respectively [27]. In another article, the results of novel thiazolidinone derivatives were even less impressive with IC_50_ equal to 12 μM and 24 μM after 24 h of treatment for MCF-7 and MDA-MB-231, accordingly [28]. Similarly to thiazolidinone derivatives, previously reported novel rhodanine structures did not display more adequate anticancer activity on the tested breast cancer cell lines [29,30]. Considering those dose-dependent results, breast cancer MDA-MB-231 and MCF-7 cells were chosen for further analysis (Figure 2).

Another method of assessing the cytotoxic effects of tested compounds, [^3^H]-thymidine incorporation assay, was applied. This method provided the capability to analyze the antiproliferative activity of novel structures on MCF-7 and MDA-MB-231 through their ability to incorporate thymidine into the cells. The acquired results further confirmed the potent activity of the **14b** compound as the most prominent structure among the novel compounds (Table 2). The test was carried out in triplicate at concentrations of 5, 15, and 30 μM for the MDA-MB-231 cell line and 1, 5, and 10 μM for the MCF-7 cell line.

#### 2.2.3. Proapoptotic Effects of Studied Compounds

**Figure 3:**
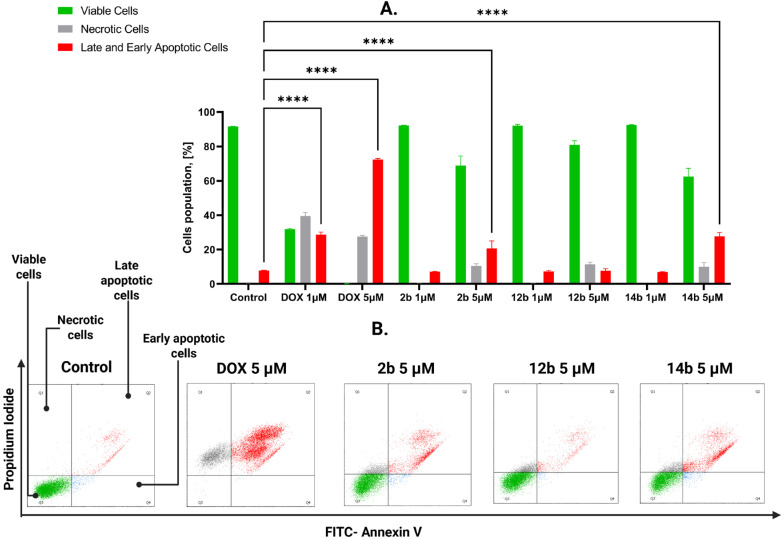
Results of flow cytometry analysis on MCF-7 cell line after 24 h of incubation with tested compounds **2b**, **12b**, **14b** and the reference drug doxorubicin in concentrations 1 and 5 μM. (**A**) The graph shows the obtained results and compares late apoptotic cells after treatment to the viable cells. Means ± SD are shown with *N* = 2. ANOVA test **** *p* ≤ 0.0001. (**B**) The graph visualizes the cytograms obtained from flow cytometry analysis. The number of repetitions for each compound and for each line is 3.

The three chosen compounds, **2b**, **12b,** and **14b,** were tested using Annexin V-FITC and propidium iodide to assess their effects on apoptosis induction on MCF-7 and MDA-MB-231 cell lines. To evaluate the population of cells, four states were determined: unstained cells (alive), cells stained with propidium iodide (necrotic), cells stained with FITC-labeled Annexin V (early apoptotic), and cells stained with both Annexin V and propidium iodide (late apoptotic). As shown in Figure 3, after 24 h of incubation, compound **2b** in concentration 1 μM on the MCF-7 cell line displayed 7.7% cells in late and early apoptosis combined, while the necrotic cell population was equal to 0.7%. In a concentration of 5 μM the values were 8.5% and 12.2%, respectively. For **12b** in a concentration equal to 1 μM, the obtained results showcased 7.2% of cells in early and late apoptosis and 0.7% in a state of necrosis. For 5 μM concentration results were 23.7% and 11.5%, respectively. For the compound **14b** the values for 1 μM concentration were 6.9% of combined apoptotic cells and 0.6% of necrotic cells, whereas for 5 μM concentration the obtained data displayed 29.2% of early and late apoptotic cells and 11.7% of necrotic cells.

**Figure 4:**
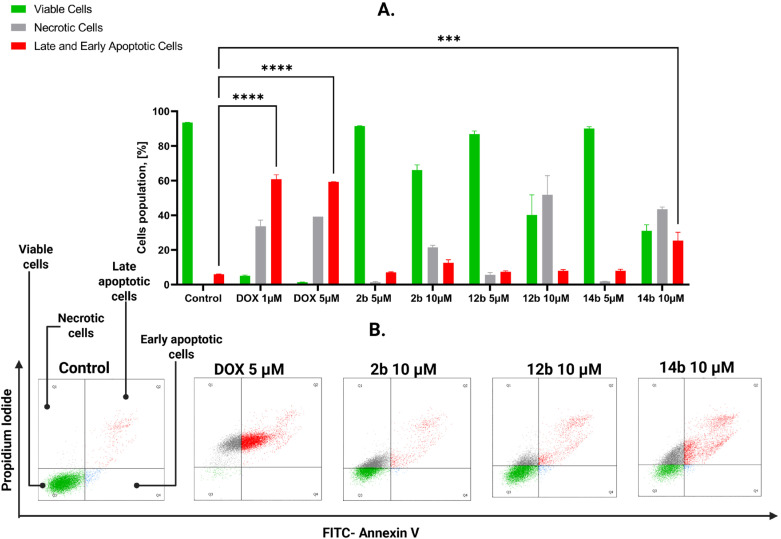
Results of flow cytometry analysis on MDA-MB-231 cell line after 24 h of incubation with tested compounds **2b**, **12b**, **14b** in concentrations 5 and 10 μM and the reference drug doxorubicin in concentration 1 and 5 μM. (**A**) The graph shows the obtained results and compares late apoptotic cells after treatment to the viable cells. Means ± SD are shown with *N* = 2. ANOVA test **** *p* ≤ 0.0001; *** *p* ≤ 0.001 (**B**) The graph visualizes the cytograms obtained from flow cytometry analysis. The number of repetitions for each compound and for each line is 3.

Similarly to Figure 3, Figure 4 showcases the flow cytometric assessment of compounds effects on apoptosis induction on the MDA-MB-231 cell line after 24 h of incubation with tested structures. For this cell line, based on previous results, higher concentrations of tested compounds were chosen: 5 and 10 μM. Compound **2b** displayed 7.8% of combined apoptotic cells and 6.6% of necrotic cells in 5 μM concentration, and 7.5% and 44% in 10 μM concentration, respectively. At the same time, compound **12b** showcased 7.3% of early and late apoptotic cells, 1.2% of necrotic cells in 5 μM concentration, and 13.8% and 22.3% in 10 μM concentration, consequently. However, the most significant results among novel structures were displayed by compound **14b** with 5.7% cells in the state of programmed cell death, 1.4% in the state of necrosis in 5 μM concentration, and 28.8% and 42.6% in 10 μM concentration, respectively.

**Figure 5:**
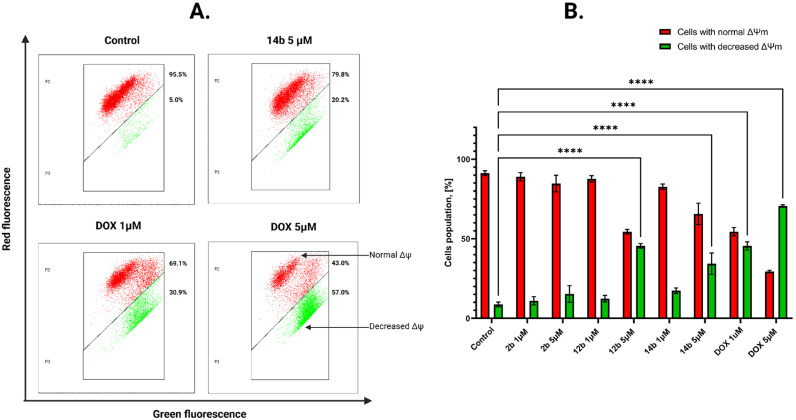
Results of flow cytometry measurement (**A**) of changes in the mitochondrial membrane potential (ΔΨm) of MCF-7 breast cancer cells after 24 h of incubation with tested compounds in concentrations 1 and 5 μM and DOX (1 and 5 μM). The graph (**B**) visualizes the cytograms from the analysis of compounds. Means ± SD are shown with *N* = 2. ANOVA tests were used to demonstrate differences between cells treated with compounds. **** *p* ≤ 0.0001. The number of repetitions for each compound and for each line is 3.

**Figure 6:**
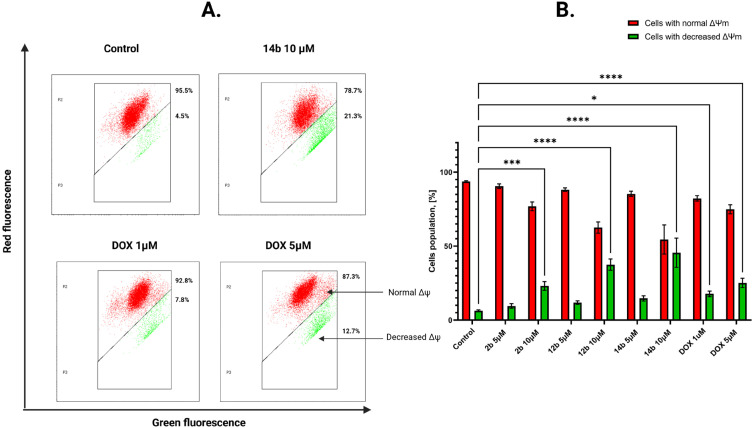
Results of flow cytometry measurement (**A**) of changes in the mitochondrial membrane potential (ΔΨm) of MDA-MB-231 breast cancer cells after 24 h of incubation with tested compounds in concentrations 5 and 10 μM and DOX (1 and 5 μM). The graph (**B**) visualizes the cytograms from the analysis of compounds. Means ± SD are shown with *N* = 2. ANOVA tests were used to demonstrate differences between cells treated with compounds. * *p* ≤ 0.5; *** *p* ≤ 0.001; **** *p* ≤ 0.0001. The number of repetitions for each compound and for each line is 3.

To examine the impact of the most active compounds, **2b**, **12b** and **14b,** on mitochondrial membrane potential (ΔΨm), the flow cytometric assessment of JC-1 staining was utilized. This method involves marking cells with fully functional mitochondria with red fluorescence due to the accumulation of the dye in hyperpolarized mitochondrial membrane. In case of a damaged membrane, the dye decomposes into monomers, emitting green fluorescence. The most active novel 4-thiazolidinone derivative, **14b**, induced a decrease in mitochondrial potential by 8.75% for 1 μM concentration and 20.25% for 5 μM concentration in the MCF-7 cell line (Figure 5). For MDA-MB-231 cells, the values were as follows: 4.25% for 5 μM and 20.95% for 10 μM concentration. The obtained results signify the compound’s capability to decrease mitochondrial potential on both cell lines at a higher concentration (Figure 6).

#### 2.2.4. Generation of Reactive Oxygen Species

**Figure 7:**
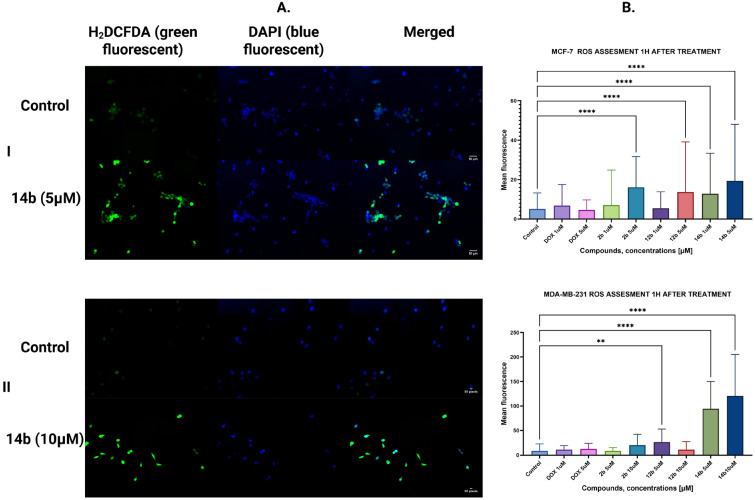
Results of H_2_DCFDA and DAPI double staining. (**I**) MCF-7 cell line, (**II**) MDA-MB-231 cell line, (**A**) representative microscope images, (**B**) graph showcasing the obtained results with one-way ANOVA test. *N* > 250; ** *p* ≤ 0.01; **** *p* ≤ 0.0001.

The double staining method was applied to analyze novel compounds’ effects on the generation of reactive oxygen species. The first dye, H_2_DCFDA, after conversion to DCF, serves as an indicator of oxidative stress (green fluorescence), while the second one, known as DAPI, was applied to showcase nuclear genetic material of MCF-7 and MDA-MB-231 breast cancer cells (blue fluorescence). The concentrations of compounds were 1 and 5 μM for the MCF-7 cell line and 5 and 10 μM for the MDA-MB-231 cell line. The incubation time after treatment was one hour in both cell lines. The compound that caused the highest generation of ROS was **14b** in all tested concentrations and cell lines (Figure 7). Interestingly enough, doxorubicin, a known oxidative stress generator [31], displayed fewer effects on reactive oxygen species generation than the most potent new 4-thiazolidinone derivative.

#### 2.2.5. Activation of the Intrinsic Mitochondrial Pathway by the Tested Compounds

**Figure 8:**
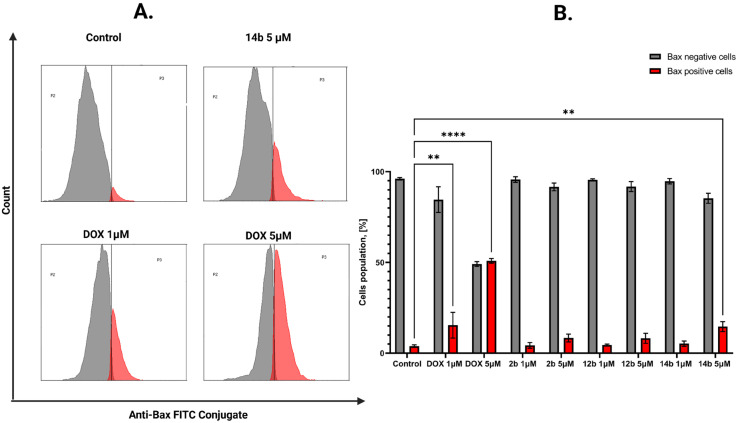
Results of flow cytometry measurement representative cytograms (**A**) of anti-Bax antibody on MCF-7 breast cancer cells after 24 h of incubation with tested compounds (1 and 5 μM) and DOX (1 and 5 μM). The graph (**B**) visualizes the cytograms from the analysis of compounds. Means ± SD are shown with *N* = 3. ANOVA tests were used to demonstrate differences between cells treated with compounds. ** *p* ≤ 0.01; **** *p* ≤ 0.0001. The number of repetitions for each compound and for each line is 3.

**Figure 9:**
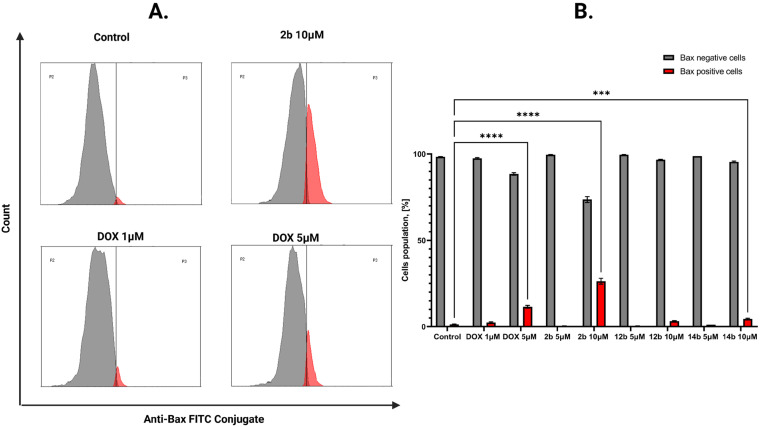
Results of flow cytometry measurement representative cytograms (**A**) of anti-Bax antibody on MDA-MB-231 breast cancer cells after 24 h of incubation with tested compounds (5 and 10 μM) and DOX (1 and 5 μM). The graph (**B**) visualizes the cytograms from the analysis of compounds. Means ± SD are shown with *N* = 3. ANOVA tests were used to demonstrate differences between cells treated with compounds. *** *p* ≤ 0.001; **** *p* ≤ 0.0001. The number of repetitions for each compound and for each line is 3.

The effect of the tested compounds on the intrinsic mitochondrial pathway of programmed cell death was further assessed. For this purpose, the concentration of the proapoptotic Bax protein from the Bcl-2 family was examined [32]. The concentration of this protein was analyzed in breast cancer cells after treatment with novel compounds at concentrations of 1 and 5 µM for the MCF-7 line and 5 and 10 µM for the MDA-MB-231 line. The most significant result on the MCF-7 cell line was obtained for the **14b** compound in 5 µM concentration, where the Bax-positive cell population was 14.67%, while in the MDA-MB-231 cell line the most notable result was displayed by compound 2b with 26.33% of cells in the Bax-positive population (Figure 8 and Figure 9).

**Figure 10:**
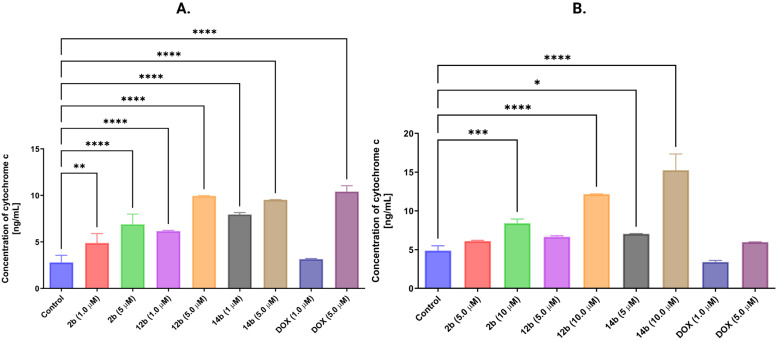
Results of ELISA method of concentration of cytochrome C in MCF-7 (**A**) and MDA-MB-231 (**B**) breast cancer cells after 24 h of incubation with compounds **2b**, **12b**, **14b** in concentrations 1 and 5 μM for MCF-7 cell line and 5 and 10 μM for MDA-MB-231 cell line. Doxorubicin’s concentrations for both lines were 1 and 5 μM. Means ± SD are shown with *N* = 3. ANOVA tests were used to demonstrate differences between cells treated with compounds. * *p* ≤ 0.5; ** *p* ≤ 0.01; *** *p* ≤ 0.001; **** *p* ≤ 0.0001. The number of repetitions for each compound and for each line is 3.

The influence of the examined compounds on the level of cytochrome C, one of the crucial proteins of the intrinsic pathway of programmed cell death, was assessed. This protein is released into the cytoplasm from the mitochondrial intermembrane space as a result of the decrease in mitochondrial membrane potential [33]. An increase in the concentration of cytochrome C was noted for all tested structures, as well as their concentrations after 24 h of treatment on both MCF-7 and MDA-MB-231 cell lines. The most significant result was reported for compound **14b** at a concentration of 5 μM in the MCF-7 cell line and 10 µM in the MDA-MB-231 cell line, where the concentration of cytochrome C was 9.54 ng/mL and 15.26 ng/mL, respectively (Figure 10).

**Figure 11:**
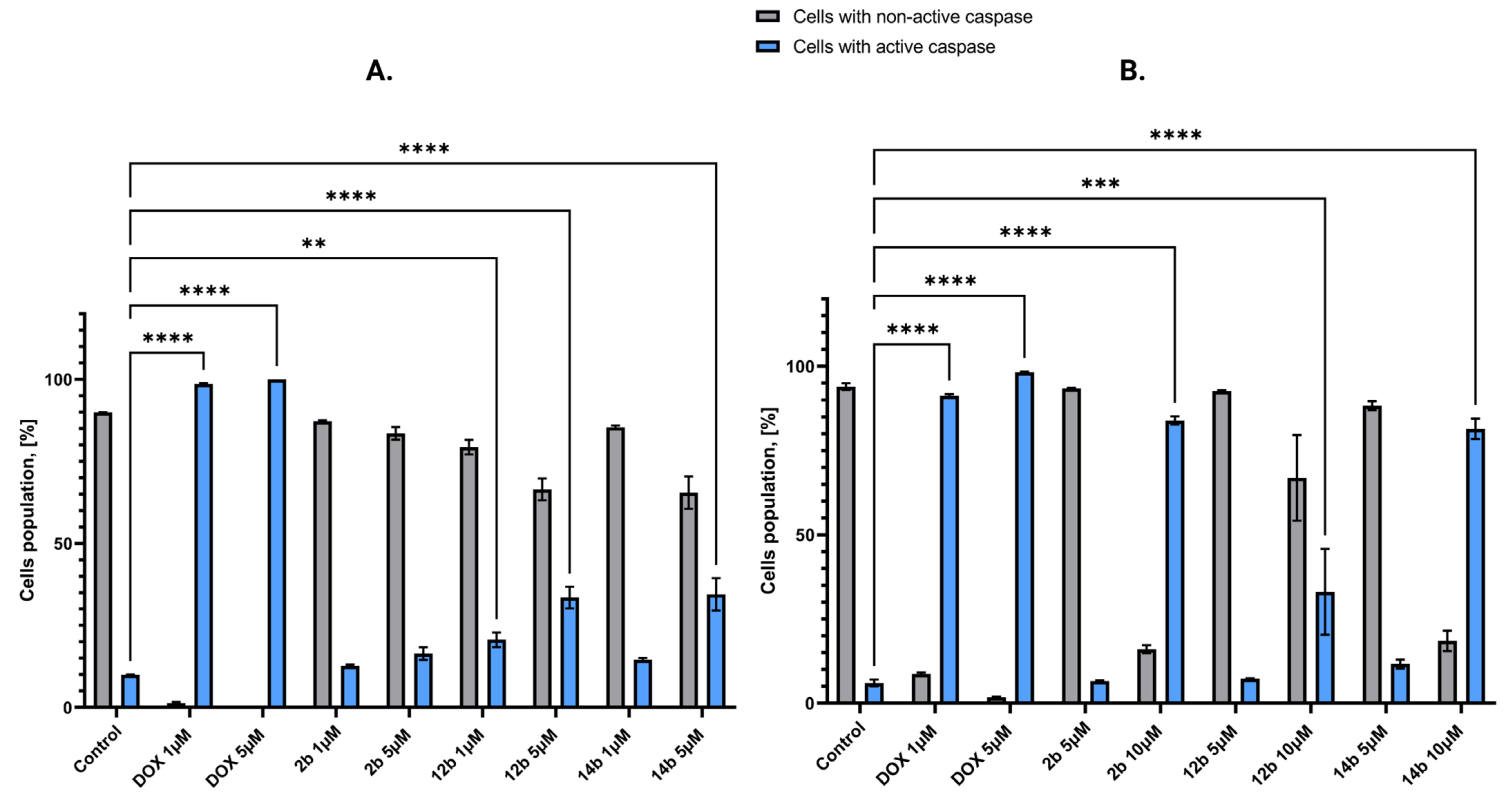
Results of flow cytometry method of concentration of caspase-9 in MCF-7 (**A**) and MDA-MB-231 (**B**) breast cancer cells after 24 h of incubation with compounds **2b**, **12b**, **14b** in concentrations 1 and 5 μM for MCF-7 cell line and 5 and 10 μM for MDA-MB-231 cell line. Doxorubicin’s concentrations for both lines were 1 and 5 μM. Means ± SD are shown with *N* = 3. ANOVA tests were used to demonstrate differences between cells treated with compounds; ** *p* ≤ 0.01; *** *p* ≤ 0.001; **** *p* ≤ 0.0001. The number of repetitions for each compound and for each line is 3.

To further analyze the compounds’ effects on the intrinsic pathway of the process of programmed cell death, it was decided to assess the expression of initiator caspase-9 on MCF-7 and MDA-MB-231 cell lines after 24 h of treatment using the flow cytometry method. Caspase-9 is an initiator caspase that directly activates executive caspase-3; as such, it has a significant effect on apoptosis [32]. In the MCF-7 line, except compound **2b**, all of the tested structures displayed statistically significant increase in caspase-9 in all tested concentrations. For the MDA-MB-231 line, the results were even more prominent, with every tested compound and concentration displaying a notable increase in cells with active caspase-9. However, the most remarkable result was observed for compound **2b** with 83.95% of cells with active caspase-9 (Figure 11).

**Figure 12:**
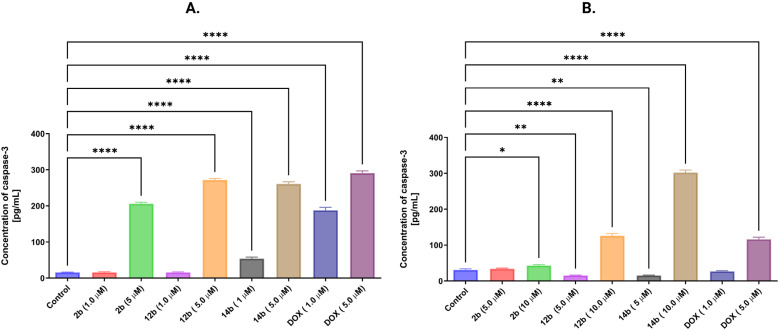
ELISA results of caspase-3 assessment in MCF-7 (**A**) and MDA-MB-231 (**B**) breast cancer cells after 24 h of incubation with compounds **2b**, **12b**, **14b** in concentrations 1 and 5 μM for MCF-7 cell line and 5 and 10 μM for MDA-MB-231 cell line. Doxorubicin’s concentrations for both lines were 1 and 5 μM. Means ± SD are shown with *N* = 3. ANOVA tests were performed to demonstrate statistical differences between cells treated with compounds. * *p* ≤ 0.5; ** *p* ≤ 0.01; **** *p* ≤ 0.0001. The number of repetitions for each compound and for each line is 3.

To confirm the activity of the caspase pathway, an effector caspase-3 was assayed on MCF-7 and MDA-MB-231 cell lines after 24 h of treatment using the ELISA method. Novel 4-thiazolidinone derivatives were tested in concentrations of 1 and 5 μM for the MCF-7 cell line and 5 and 10 μM for the MDA-MB-231 cell line. All of the new compounds showcased an increase in caspase-3 concentration in MCF-7 cells at a concentration equal to 5 μM, with compound **14b** also displaying a statistically significant result in 1 μM concentration. Additionally, compound **14b** exhibited astonishing results on the MDA-MB-231 cell line, where the tested structure in a concentration of 10 μM increased caspase-3 concentration at least two times more than was observed in other compounds and concentrations, including the positive control, doxorubicin. Compound **12b** in a concentration equal to 10 μM displayed a significant increase in caspase-3 as well. Interestingly enough, compound **2b** displayed a slight increase in caspase-3 concentration at its highest concentration (Figure 12).

## 3. Discussion

Cancer is the second leading cause of death worldwide, after cardiovascular disease. Every year, 20 million people are diagnosed with cancer, of which 2.3 million are people with breast cancer, which accounts for 11.6% of all cancers [34]. Traditional treatment for breast cancer includes radiotherapy, tumor resection, endocrine therapy, immunotherapy, and chemotherapy, with each therapy being personalized and depending on the type of cancer, the patients’ condition, and their age. The continuously growing concern is the ability of cancer cells to develop resistance towards frequently used anticancer drugs from the anthracycline group (doxorubicin, epirubicin), taxanes (taxol, docetaxel), or alkylating agents (cisplatin, carboplatin, cyclophosphamide) [35,36]. The design and synthesis of compounds with multifaceted mechanisms of action are increasingly used in cancer treatment. This allows for the avoidance of potential tumor resistance in comparison to traditional drugs with a single-directional mechanism [37,38]. The compounds presented in this paper demonstrated multifaceted anticancer activity towards numerous breast cancer cell lines. Additionally, it is worth mentioning that the most promising novel compound, **14b**, displayed remarkably potent anticancer activity with IC_50_ equal to 0.85 µM after 24 h of treatment, which was comparable to doxorubicin, with IC_50_ of 0.73 µM, and superior to cisplatin results, which required a concentration of around 80µM to inhibit more than 50% of the MCF-7 and MDA-MB-231 [39].

**Figure 13:**
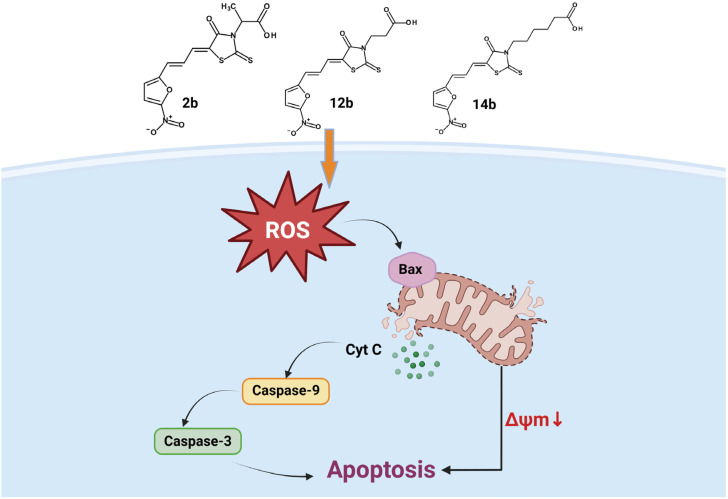
Mechanism of action of 5-((5-nitrofuran-2-yl)allylidene)-2-thioxo-4-thiazolidinones.

Based on the results obtained in biological tests, we noted the impact of compounds on the two most important structures in the cell genetic material and mitochondria (Figure 13). The damaging effect on mitochondria by tested compounds can be attributed to the increased generation of reactive oxygen species. This starts a cascade of activations of numerous proapoptotic factors, such as increase in Bax protein expression, decrease in mitochondrial potential, release of cytochrome C, and increased caspases-9 and -3 concentrations in cancer cells [40]. Increasing the concentration of Bax protein causes permeabilization of the mitochondrial membrane [41]. It reduces mitochondrial potential, which disrupts the synthesis of ATP, the main energy carrier in the cell, which addresses the cell into the apoptotic pathway [42]. Due to the damaged mitochondrial membrane, cytochrome C is released into the cytosol. This protein is located in the intermembrane space of the mitochondria, playing a role as an electron transporter in the respiratory chain. However, it also serves as a signal along the intrinsic pathway of apoptosis. As such, released cytochrome C leads to the activation of initiator caspase-9, which, in turn, causes the activation of effector caspase-3. This protein is a final step in the intrinsic pathway of apoptosis and the one that ultimately leads the cell to the process of programmed cell death [43].

At the same time, the influence on genetic material was demonstrated by tests for the incorporation of [^3^H]-thymidine which allowed for the assessment of the proliferation inhibition and the effect of compounds on DNA structure [44]. The tested compounds effectively inhibited the incorporation of [^3^H]-thymidine into the DNA chain even at lower concentrations.

**Figure 14:**
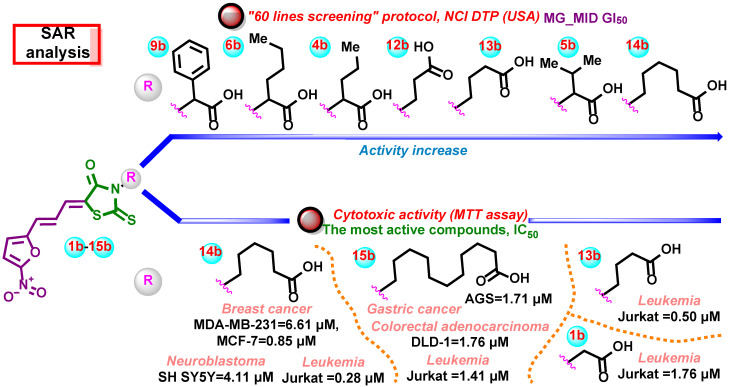
Preliminary structure–anticancer activity relationships for the synthesized 5-((5-nitrofuran-2-yl)allylidene)-2-thioxo-4-thiazolidinones.

Based on the anticancer activity screening results, some preliminary structure–activity relationships for the synthesized 5-((5-nitrofuran-2-yl)allylidene)-2-thioxo-4-thiazolidinones have been possible to conclude (Figure 14).

Regarding the preliminary SAR analysis of prescreening data, it is worth mentioning that the presence of the 5-(5-nitrofuran-2-yl)allylidene moiety turned out to be a necessary requirement for achieving the anticancer effects. It is worth mentioning that the presence of the 5-(5-nitrofuran-2-yl)allylidene moiety led to the almost equal activity level compared with derivatives containing 5-(2-chloro-3-(4-nitrophenyl))allylidene (Ciminalum) substituent [2]. The nature of the COOH-bearing moiety at the N3 position is a crucial factor that leads to the selective impact on cell lines as well as the potency of the effects. The obtained results indicate that the molecules with unbranched alkyl chains were the most active in “60 lines screening” as well as in the MTT assay (Figure 14). However, this statement requires further studies and tests with a larger library of structurally modified analogs.

## 4. Materials and Methods

### 4.1. Chemistry

Melting points were measured in open capillary tubes on a Cole-Parmer IA9200 melting point apparatus (Antylia Scientific Ltd., Stone, UK) and are uncorrected. The elemental analyses (C, H, N) were performed using the Thermo Scientific FlashSmart Elemental Analyzer (Thermo Fisher Scientific Inc., Waltham, MA, USA) and were within ±0.4% of the theoretical values. The 400 MHz-^1^H and 100 MHz-^13^C spectra were recorded on a Varian Unity Plus 400 (400 MHz) spectrometer (Varian Inc., Paulo Alto, CA, USA). All spectra were recorded at room temperature, except where indicated otherwise, and were referenced internally to solvent reference frequencies. Chemical shifts (δ) are quoted in ppm and coupling constants (*J*) are reported in Hz. LC–MS spectra were obtained on a Finnigan MAT INCOS-50 (Thermo Finnigan LLC, San Jose, CA, USA). The infrared spectra *υ* cm^−1^ (KBr) were determined on a Shimadzu IRSpirit-TX spectrometer (Kyoto, Japan). The reaction mixture was monitored by thin layer chromatography (TLC) using commercial glass-backed TLC plates (Merck Kieselgel 60 F254, Darmstadt, Germany). Solvents and reagents which are commercially available were used without further purification.

The synthesis of corresponding rhodanine-3-carboxylic acids was carried out according to methods described in the references: for compounds **1a**, **5a**, **7a**, **8a** in [45], **2a**–**4a**, **6a** in [46], **9a** in [47], **10a** in [48], **11a** in [49], and **12a**–**15a** in [50], respectively. Synthesis of 5-ylidene derivatives **1b–15b** was performed following the protocol reported in Finiuk et al. (2022) [5]. Purification of the compounds was carried out by recrystallization from isopropanol for the **15a**; glacial acetic acid for the **1b**–**6b**, **9b**, and **11b**–**15b**; and mixture glacial acetic acid–water (1:1) for the **7b**, **8b**, and **10b**.

Doxorubicin hydrochloride (DOX) was purchased from Sigma-Aldrich (St. Louis, MO, USA).

11-(4-Oxo-2-thioxothiazolidin-3-yl)undecanoic acid (**15a**)

Yellow powder, yield 91%, MP 89–91 °C (*i*-PrOH). ^1^H NMR (400 MHz, DMSO-*d*_6_, δ): 11.93 (s, 1H, COOH), 4.23 (s, 2H, CH_2_), 3.82 (t, *J* = 7.5 Hz, 2H, CH_2_), 2.17 (t, *J* = 7.4 Hz, 2H, CH_2_), 1.49 (dt, *J* = 27.7, 7.3 Hz, 4H, 2*CH_2_), 1.23 (d, *J* = 8.4 Hz, 12H, 6*CH_2_). ^13^C NMR (100 MHz, DMSO-*d*_6_, δ): 203.1 (C=S), 174.4 (C=O), 174.3 (C=O), 45.1 (CH_2_), 43.8 (CH_2_), 35.7 (CH_2_), 33.6 (CH_2_), 28.8 (CH_2_), 28.7 (CH_2_), 28.6 (CH_2_), 28.4 (CH_2_), 26.1 (CH_2_), 26.0 (CH_2_), 24.4 (CH_2_). LCMS (ESI+) *m/z* 318.0 (100.0%, [M+H]^+^). Anal. calc. for C_14_H_23_NO_3_S_2_: C, 52.97%; H, 7.30%; N, 4.41%. Found: C, 53.10%; H, 7.50%; N, 4.60%.

2-((*Z*)-5-((*E*)-3-(5-Nitrofuran-2-yl)allylidene)-4-oxo-2-thioxothiazolidin-3-yl)acetic acid (**1b**)

Orange-brown powder, yield 80%, MP 232 with decomp. °C (AcOH). ^1^H NMR (400 MHz, DMSO-*d*_6_, δ): 13.47 (brs, 1H, COOH), 7.80 (d, *J* = 4.0 Hz, 1H, furan), 7.66 (d, *J* = 11.7 Hz, 1H, CH=), 7.44–7.34 (m, 2H, furan+ CH=), 7.15 (dd, *J* = 15.1, 11.7 Hz, 1H, CH=), 4.72 (s, 2H, CH_2_). ^13^C NMR (100 MHz, DMSO-*d*_6_, δ): 192.3 (C=S), 167.2 (C=O), 165.5 (C=O), 154.0 (furan), 151.8 (furan), 131.9 (CH=), 129.0 (CH=), 127.6 (C-5), 126.7 (furan), 115.8 (CH=), 115.2 (furan), 44.9 (CH_2_). IR (KBr): 1733 and 1700 (C=O), 1577 and 1559 (C=C), 1514 and 1312 (N-O) cm^−1^. LCMS (ESI+) *m/z* 341.0 (98.7%, [M+H]^+^). Anal. calc. for C_12_H_8_N_2_O_6_S_2_: C, 42.35%; H, 2.37%; N, 8.23%. Found: C, 42.50%; H, 2.50%; N, 8.40%.

2-((*Z*)-5-((*E*)-3-(5-Nitrofuran-2-yl)allylidene)-4-oxo-2-thioxothiazolidin-3-yl)propanoic acid (**2b**)

Brown powder, yield 87%, MP >250 °C (AcOH). ^1^H NMR (400 MHz, DMSO-*d*_6_, δ): 13.23 (brs, 1H, COOH), 7.80 (d, *J* = 4.0 Hz, 1H, furan), 7.59 (d, *J* = 11.7 Hz, 1H, CH=), 7.43–7.33 (m, 2H, furan+ CH=), 7.15 (dd, *J* = 15.2, 11.8 Hz, 1H, CH=), 5.62–5.54 (m, 1H, CH), 1.52 (d, *J* = 7.2 Hz, 3H, CH_3_). ^13^C NMR (100 MHz, DMSO-*d*_6_, δ): 191.9 (C=S), 169.4 (C=O), 165.5 (C=O), 154.0 (furan), 151.8 (furan), 131.5 (CH=), 128.9 (CH=), 127.5 (C-5), 126.3 (furan), 115.8 (CH=), 115.2 (furan), 52.8 (CH), 13.32 (CH_3_). IR (KBr): 1743 and 1696 (C=O), 1580 and 1559 (C=C), 1507 and 1339 (N-O) cm^−1^. LCMS (ESI+) *m/z* 355.0 (97.0%, [M+H]^+^). Anal. calc. for C_13_H_10_N_2_O_6_S_2_: C, 44.06%; H, 2.84%; N, 7.91%. Found: C, 44.20%; H, 3.00%; N, 8.10%.

2-((*Z*)-5-((*E*)-3-(5-Nitrofuran-2-yl)allylidene)-4-oxo-2-thioxothiazolidin-3-yl)butanoic acid (**3b**)

Light brown powder, yield 86%, MP 223 with decomp. °C (AcOH). ^1^H NMR (400 MHz, DMSO-*d*_6_, δ): 13.29 (brs, 1H, COOH), 7.80 (d, *J* = 4.0 Hz, 1H, furan), 7.60 (d, *J* = 11.7 Hz, 1H, CH=), 7.43–7.34 (m, 2H, furan+ CH=), 7.14 (dd, *J* = 15.1, 11.7 Hz, 1H, CH=), 5.46 (t, *J* = 7.8 Hz, 1H, CH), 2.17 (dq, *J* = 12.2, 6.9, 6.4 Hz, 2H, CH_2_), 0.83 (t, *J* = 7.4 Hz, 3H, CH_3_). ^13^C NMR (100 MHz, DMSO-*d*_6_, δ): 193.1 (C=S), 168.9 (C=O), 166.1 (C=O), 154.0 (furan), 151.6 (furan), 131.8 (CH=), 129.1 (CH=), 127.5 (C-5), 126.7 (furan), 115.9 (CH=), 115.3 (furan), 58.6 (CH), 20.9 (CH_2_), 10.6 (CH_3_). IR (KBr): 1718 and 1705 (C=O), 1586 and 1559 (C=C), 1515 and 1339 (N-O) cm^−1^. LCMS (ESI+) *m/z* 369.0 (100.0%, [M+H]^+^). Anal. calc. for C_14_H_12_N_2_O_6_S_2_: C, 45.65%; H, 3.28%; N, 7.60%. Found: C, 45.80%; H, 3.50%; N, 7.80%.

2-((*Z*)-5-((*E*)-3-(5-Nitrofuran-2-yl)allylidene)-4-oxo-2-thioxothiazolidin-3-yl)pentanoic acid (**4b**)

Brown powder, yield 82%, MP 220 with decomp. °C (AcOH). ^1^H NMR (400 MHz, DMSO-*d*_6_, δ): 13.29 (brs, 1H, COOH), 7.79 (d, *J* = 4.0 Hz, 1H, furan), 7.60 (d, *J* = 11.8 Hz, 1H, CH=), 7.43–7.34 (m, 2H, furan+ CH=), 7.17 (dd, *J* = 15.0, 11.7 Hz, 1H, CH=), 5.53 (s, 1H, CH), 2.12 (h, *J* = 9.2, 7.9 Hz, 2H, CH_2_), 1.23 (dq, *J* = 15.2, 7.3 Hz, 2H, CH_2_), 0.87 (t, *J* = 7.3 Hz, 3H, CH3). ^13^C NMR (100 MHz, DMSO-*d*_6_, δ): 191.9 (C=S), 169.0 (C=O), 165.1 (C=O), 154.0 (furan), 151.8 (furan), 131.9 (CH=), 129.0 (CH=), 127.5 (furan), 127.6 (C-5), 115.9 (CH=), 115.2 (furan), 57.0 (CH), 29.5 (CH_2_), 18.9 (CH_2_), 13.5 (CH_3_). IR (KBr): 1718 and 1702 (C=O), 1573 and 1559 (C=C), 1507 and 1339 (N-O) cm^−1^. LCMS (ESI+) *m/z* 383.0 (100.0%, [M+H]+). Anal. calc. for C_15_H_14_N_2_O_6_S_2_: C, 47.11%; H, 3.69%; N, 7.33%. Found: C, 47.30%; H, 3.90%; N, 7.50%.

3-Methyl-2-((*Z*)-5-((*E*)-3-(5-nitrofuran-2-yl)allylidene)-4-oxo-2-thioxothiazolidin-3-yl)butanoic acid (**5b**)

Orange-brown powder, yield 85%, MP 213 with decomp. °C (AcOH). ^1^H NMR (400 MHz, DMSO-*d*_6_, δ): 13.18 (s, 1H, COOH), 7.80 (d, *J* = 4.0 Hz, 1H, furan), 7.63 (d, *J* = 11.7 Hz, 1H, CH=), 7.44–7.34 (m, 2H, furan+ CH=), 7.15 (dd, *J* = 15.0, 11.7 Hz, 1H, CH=), 5.17–5.11 (m, 1H, CH), 2.75–2.67 (m, 1H, CH), 1.18 (d, *J* = 6.5 Hz, 3H, CH_3_), 0.73 (d, *J* = 6.9 Hz, 3H, CH_3_). ^13^C NMR (100 MHz, DMSO-*d*_6_, δ): 188.9 (C=S), 168.5 (C=O), 165.8 (C=O), 154.0 (furan), 151.9 (furan), 132.3 (CH=), 129.3 (CH=), 127.5 (C-5), 125.5 (furan), 115.9 (CH=), 115.3 (furan), 62.1 (CH), 27.1 (CH), 21.6 (CH_3_), 18.8 (CH_3_). IR (KBr): 1718 and 1702 (C=O), 1573 and 1559 (C=C), 1507 and 1339 (N-O) cm^−1^. LCMS (ESI+) *m/z* 383.0 (100.0%, [M+H]^+^). Anal. calc. for C_15_H_14_N_2_O_6_S_2_: C, 47.11%; H, 3.69%; N, 7.33%. Found: C, 47.30%; H, 3.90%; N, 7.50%.

2-((*Z*)-5-((*E*)-3-(5-nitrofuran-2-yl)allylidene)-4-oxo-2-thioxothiazolidin-3-yl)hexanoic acid (**6b**)

Brown powder, yield 87%, MP 200 with decomp. °C (AcOH). ^1^H NMR (400 MHz, DMSO-*d*_6_, δ): 13.22 (brs, 1H, COOH), 7.79 (d, *J* = 4.0 Hz, 1H, furan), 7.59 (d, *J* = 11.7 Hz, 1H, CH=), 7.42–7.32 (m, 2H, furan+ CH=), 7.16 (dd, *J* = 15.0, 11.8 Hz, 1H, CH=), 5.51 (s, 1H, CH), 2.15 (q, J = 7.5 Hz, 2H, CH_2_), 1.27–1.14 (m, 2H, 2*CH_2_), 0.82 (t, *J* = 6.6 Hz, 3H, CH_3_). ^13^C NMR (100 MHz, DMSO-*d*_6_, δ): 192.9 (C=S), 169.0 (C=O), 165.7 (C=O), 154.0 (furan), 151.8 (furan), 131.9 (CH=), 129.0 (CH=), 127.4 (C-5), 125.9 (furan), 115.9 (CH=), 115.2 (furan), 57.2 (CH), 27.75 (CH_2_), 27.14 (CH_2_), 21.75 (CH_2_), 13.68 (CH_3_). IR (KBr): 1718 and 1700 (C=O), 1579 and 1559 (C=C), 1507 and 1339 (N-O) cm^−1^. LCMS (ESI+) *m/z* 397.0 (100.0%, [M+H]^+^). Anal. calc. for C_16_H_16_N_2_O_6_S_2_: C, 48.48%; H, 4.07%; N, 7.07%. Found: C, 48.70%; H, 4.20%; N, 7.30%.

4-Methyl-2-((*Z*)-5-((*E*)-3-(5-nitrofuran-2-yl)allylidene)-4-oxo-2-thioxothiazolidin-3-yl)pentanoic acid (**7b**)

Red-brown powder, yield 81%, MP 111–114 °C (AcOH:H2O). ^1^H NMR (400 MHz, DMSO-*d*_6_, δ): 13.32 (brs, 1H, COOH), 7.79 (d, *J* = 4.1 Hz, 1H, furan), 7.59 (d, *J* = 11.8 Hz, 1H, CH=), 7.42–7.33 (m, 2H, furan+ CH=), 7.13 (dd, *J* = 15.0, 11.6 Hz, 1H, CH=), 5.54 (s, 1H, CH), 2.15 (s, 1H, CH), 1.99 (ddd, *J* = 14.4, 9.2, 4.7 Hz, 1H, CH2), 1.47 (dd, *J* = 13.4, 7.0 Hz, 1H, CH_2_), 0.91 (d, *J* = 6.5 Hz, 3H, CH_3_), 0.86 (d, *J* = 6.6 Hz, 3H, CH_3_). ^13^C NMR (100 MHz, DMSO-*d*_6_, δ): 192.6 (C=S), 169.2 (C=O), 165.8 (C=O), 154.0 (furan), 151.8 (furan), 131.9 (CH=), 129.0 (CH=), 127.5 (C-5), 125.2 (furan), 115.9 (CH=), 115.2 (furan), 55.8 (CH), 36.33 (CH), 24.75 (CH_2_), 22.78 (CH_3_), 21.88 (CH_3_). IR (KBr): 1714 and 1700 (C=O), 1581 and 1559 (C=C), 1507 and 1339 (N-O) cm^−1^. LCMS (ESI+) *m/z* 397.0 (98.7%, [M+H]^+^). Anal. calc. for C_16_H_16_N_2_O_6_S_2_: C, 48.48%; H, 4.07%; N, 7.07%. Found: C, 48.60%; H, 4.20%; N, 7.20%.

3-Methyl-2-((*Z*)-5-((*E*)-3-(5-nitrofuran-2-yl)allylidene)-4-oxo-2-thioxothiazolidin-3-yl)pentanoic acid (**8b**)

Brown powder, yield 88%, MP 109–112 °C (AcOH:H2O). ^1^H NMR (400 MHz, DMSO-*d*_6_, δ): 13.24 (brs, 1H, COOH), 7.80 (d, *J* = 4.2 Hz, 1H, furan), 7.62 (d, *J* = 11.7 Hz, 1H, CH=), 7.44–7.34 (m, 2H, furan+ CH=), 7.16 (dd, *J* = 15.1, 11.7 Hz, 1H, CH=), 5.18 (d, *J* = 7.4 Hz, 1H, CH), 2.50 (m, 1H, CH), 1.23 (m, 1H, CH_2_) 1.14 (d, *J* = 6.5 Hz, 3H), 0.93 (m, 1H, CH_2_), 0.79 (t, *J* = 7.3 Hz, 3H). ^13^C NMR (100 MHz, DMSO-*d*_6_, δ): 192.8 (C=S), 168.1 (C=O), 163.3 (C=O), 154.0 (furan), 151.1 (furan), 132.3 (CH=), 129.2 (CH=), 127.4 (C-5), 123.3 (furan), 115.9 (CH=), 115.2 (furan), 64.5 (CH), 61.5 (CH), 24.8 (CH_2_), 17.4 (CH3), 10.8 (CH_3_). IR (KBr): 1718 and 1700 (C=O), 1577 and 1559 (C=C), 1507 and 1339 (N-O) cm^−1^. LCMS (ESI+) *m/z* 397.0 (100.0%, [M+H]^+^). Anal. calc. for C_16_H_16_N_2_O_6_S_2_: C, 48.48%; H, 4.07%; N, 7.07%. Found: C, 48.70%; H, 4.30%; N, 7.20%.

2-((*Z*)-5-((*E*)-3-(5-Nitrofuran-2-yl)allylidene)-4-oxo-2-thioxothiazolidin-3-yl)-2-phenylacetic acid (**9b**)

Orange-brown powder, yield 85%, MP 213 with decomp. °C (AcOH). ^1^H NMR (400 MHz, DMSO-*d*_6_, δ): 13.56 (brs, 1H, COOH), 7.79 (d, *J* = 3.8 Hz, 1H, furan), 7.63 (d, *J* = 11.7 Hz, 1H, CH=), 7.58–7.44 (m, 2H, furan+ CH=), 7.40–7.32 (m, 5H, arom+CH=), 7.24–7.08 (m, 1H, arom), 6.77 (s, 1H, CH). ^13^C NMR (100 MHz, DMSO-*d*_6_, δ): 192.1 (C=S), 167.5 (C=O), 165.4 (C=O), 153.9 (furan), 151.9 (furan), 133.2 (arom), 132.4 (CH=), 129.5 (arom), 129.0 (CH=), 128.3 (arom), 128.0 (arom), 127.5 (C-5), 125.6 (furan), 116.0 (CH=), 115.2 (furan), 60.1 (CH). IR (KBr): 1718 and 1696 (C=O), 1577 and 1559 (C=C), 1507 and 1346 (N-O) cm^−1^. LCMS (ESI+) *m/z* 417.0 (100.0%, [M+H]^+^). Anal. calc. for C_18_H_12_N_2_O_6_S_2_: C, 51.92%; H, 2.90%; N, 6.73%. Found: C, 52.10%; H, 3.10%; N, 6.90%.

2-((*Z*)-5-((*E*)-3-(5-Nitrofuran-2-yl)allylidene)-4-oxo-2-thioxothiazolidin-3-yl)-3-phenylpropanoic acid (**10b**)

Orange-red powder, yield 79%, MP 127–130 °C (AcOH:H_2_O). ^1^H NMR (400 MHz, DMSO-*d*_6_, δ): 13.48 (brs, 1H, COOH), 7.79 (d, *J* = 4.1 Hz, 1H, furan), 7.55 (d, *J* = 11.7 Hz, 1H, CH=), 7.40–7.32 (m, 2H, furan+ CH=+arom), 7.28–7.16 (m, 3H, CH=+arom), 7.14–7.02 (m, 3H, arom) 5.85 (s, 1H, CH), 3.48 (s, 2H, CH2). ^13^C NMR (100 MHz, DMSO-*d*_6_, δ): 194.2 (C=S), 176.0 (C=O), 169.1 (C=O), 154.6 (furan), 151.4 (furan), 133.4 (arom),132.9 (CH=), 129.2 (CH=), 128.8 (arom), 128.1 (arom), 126.6 (C-5), 126.0 (furan), 116.5 (arom), 115.9 (CH=), 115.0 (furan), 53.1 (CH), 33.0 (CH_2_). IR (KBr): 1715 and 1700 (C=O), 1577 and 1559 (C=C), 1507 and 1339 (N-O) cm^−1^. LCMS (ESI+) *m/z* 431.0 (100.0%, [M+H]^+^). Anal. calc. for C_19_H_14_N_2_O_6_S_2_: C, 53.02%; H, 3.28%; N, 6.51%. Found: C, 53.20%; H, 3.40%; N, 6.70%.

3-(4-Hydroxyphenyl)-2-((*Z*)-5-((*E*)-3-(5-nitrofuran-2-yl)allylidene)-4-oxo-2-thioxothiazolidin-3-yl)propanoic acid (**11b**)

Brown powder, yield 83%, MP >250 °C (AcOH). ^1^H NMR (400 MHz, DMSO-*d*_6_, δ): 13.35 (brs, 1H, COOH), 9.21 (s, 1H, OH), 7.80 (d, *J* = 4.1 Hz, 1H, furan), 7.56 (d, *J* = 11.9 Hz, 1H, CH=), 7.41–7.32 (m, 2H, furan+ CH=), 7.09 (dd, *J* = 15.0, 11.8 Hz, 1H, CH=), 6.90 (d, *J* = 7.9 Hz, 2H, arom.), 6.57 (d, *J* = 7.9 Hz, 2H, arom.), 5.75 (s, 1H, CH), 3.35 (s, 2H, CH_2_). ^13^C NMR (100 MHz, DMSO-*d*_6_, δ): 192.9 (C=S), 176.0 (C=O), 168.7 (C=O), 155.9 (arom), 154.0 (furan), 151.8 (furan), 131.9 (CH=), 129.8 (CH=), 129.1 (arom), 128.8 (arom), 127.5 (C-5), 126.3 (furan), 115.8 (arom), 115.2 (CH=), 115.0 (furan), 58.4 (CH), 32.2 (CH_2_). IR (KBr): 1735 and 1700 (C=O), 1584 and 1559 (C=C), 1507 and 1339 (N-O) cm^−1^. LCMS (ESI+) *m/z* 447.0 (100.0%, [M+H]^+^). Anal. calc. for C_19_H_14_N_2_O_7_S_2_: C, 51.12%; H, 3.16%; N, 6.27%. Found: C, 51.30%; H, 3.30%; N, 6.40%.

3-((*Z*)-5-((*E*)-3-(5-Nitrofuran-2-yl)allylidene)-4-oxo-2-thioxothiazolidin-3-yl)propanoic acid (**12b**)

Orange-brown powder, yield 83%, MP 242 with decomp. °C (AcOH). ^1^H NMR (400 MHz, DMSO-*d*_6_, δ): 12.50 (s, 1H, COOH), 7.79 (d, *J* = 4.0 Hz, 1H, furan), 7.57 (d, *J* = 11.7 Hz, 1H, CH=), 7.40–7.32 (m, 2H, furan+ CH=), 7.11 (dd, *J* = 15.2, 11.8 Hz, 1H, CH=), 4.19 (t, *J* = 7.8 Hz, 2H, CH2), 2.61 (t, *J* = 7.8 Hz, 2H, CH2). ^13^C NMR (100 MHz, DMSO-*d*_6_, δ): 192.4 (C=S), 171.7 (C=O), 165.9 (C=O), 154.1 (furan), 151.8 (furan), 130.9 (CH=), 128.5 (CH=), 127.7 (C-5), 127.5 (furan), 115.6 (CH=), 115.3 (furan), 40.4 (CH_2_), 30.7 (CH_2_). IR (KBr): 1700 and 1685 (C=O), 1577 and 1559 (C=C), 1507 and 1331 (N-O) cm^−1^. LCMS (ESI+) *m/z* 355.0 (100.0%, [M+H]^+^). Anal. calc. for C_13_H_10_N_2_O_6_S_2_: C, 44.06%; H, 2.84%; N, 7.91%. Found: C, 44.20%; H, 3.00%; N, 8.10%.

4-((*Z*)-5-((*E*)-3-(5-Nitrofuran-2-yl)allylidene)-4-oxo-2-thioxothiazolidin-3-yl)butanoic acid (**13b**)

Orange-brown powder, yield 83%, MP 205 with decomp. °C (AcOH). ^1^H NMR (400 MHz, DMSO-*d*_6_, δ): 12.11 (s, 1H, COOH), 7.80 (d, *J* = 4.0 Hz, 1H, furan), 7.56 (d, *J* = 11.7 Hz, 1H, CH=), 7.40–7.32 (m, 2H, furan+ CH=), 7.11 (dd, *J* = 15.2, 11.8 Hz, 1H, CH=), 4.04 (t, *J* = 6.9 Hz, 2H, CH_2_), 2.29 (t, *J* = 7.2 Hz, 2H, CH2), 1.87 (dd, *J* = 15.8, 8.8 Hz, 2H, CH_2_). ^13^C NMR (100 MHz, DMSO-*d*_6_, δ): 192.7 (C=S), 173.6 (C=O), 166.3 (C=O), 154.1 (furan), 151.7 (furan), 130.6 (CH=), 128.3 (CH=), 127.7 (C-5), 126.6 (furan), 115.5 (CH=), 115.3 (furan), 43.6 (CH_2_), 30.9 (CH_2_), 21.9 (CH_2_). IR (KBr): 1716 and 1696 (C=O), 1577 and 1559 (C=C), 1507 and 1338 (N-O) cm^−1^. LCMS (ESI+) *m/z* 369.0 (100.0%, [M+H]^+^). Anal. calc. for C_14_H_12_N_2_O_6_S_2_: C, 45.65%; H, 3.28%; N, 7.60%. Found: C, 45.80%; H, 3.40%; N, 7.80%.

6-((*Z*)-5-((*E*)-3-(5-nitrofuran-2-yl)allylidene)-4-oxo-2-thioxothiazolidin-3-yl)hexanoic acid (**14b**)

Brown powder, yield 84%, MP 179 with decomp. °C (AcOH). ^1^H NMR (400 MHz, DMSO-*d*_6_, δ): 11.98 (s, 1H, COOH), 7.78 (d, *J* = 3.9 Hz, 1H, furan), 7.56 (d, *J* = 11.8 Hz, 1H, CH=), 7.41–7.35 (m, 2H, furan+ CH=), 7.09 (dd, *J* = 15.4, 11.8 Hz, 1H, CH=), 3.97 (t, *J* = 7.5 Hz, 2H, CH_2_), 2.20 (t, *J* = 7.2 Hz, 2H, CH_2_), 1.62 (p, *J* = 7.5 Hz, 2H, CH_2_), 1.52 (p, *J* = 7.5 Hz, 2H, CH_2_), 1.29 (p, *J* = 7.8 Hz, 2H, CH_2_). ^13^C NMR (100 MHz, DMSO-*d*_6_, δ): 192.9 (C=S), 174.7 (C=O), 166.6 (C=O), 154.6 (furan), 152.3 (furan), 131.3 (CH=), 128.9 (CH=), 128.2 (C-5), 128.0 (furan), 116.1 (CH=), 115.7 (furan), 44.5 (CH_2_), 33.8 (CH_2_), 26.5 (CH_2_), 26.1 (CH_2_), 24.5 (CH_2_). IR (KBr): 1700 and 1685 (C=O), 1577 and 1559 (C=C), 1507 and 1339 (N-O) cm^−1^. LCMS (ESI+) *m/z* 397.0 (96.8%, [M+H]^+^). Anal. calc. for C_16_H_16_N_2_O_6_S_2_: C, 48.48%; H, 4.07%; N, 7.07%. Found: C, 48.60%; H, 4.20%; N, 7.20%.

11-((*Z*)-5-((*E*)-3-(5-nitrofuran-2-yl)allylidene)-4-oxo-2-thioxothiazolidin-3-yl)undecanoic acid (**15b**)

Brown powder, yield 88%, MP 153 with decomp. °C (AcOH). ^1^H NMR (400 MHz, DMSO-*d*_6_, δ): 11.96 (brs, 1H, COOH), 7.79 (d, *J* = 4.0 Hz, 1H, furan), 7.57 (d, *J* = 11.8 Hz, 1H, CH=), 7.40–7.32 (m, 2H, furan+ CH=), 7.10 (dd, *J* = 15.1, 11.7 Hz, 1H, CH=), 3.97 (t, *J* = 7.5 Hz, 2H, CH_2_), 2.18 (t, *J* = 7.3 Hz, 2H, CH_2_), 1.59 (d, *J* = 9.4 Hz, 2H, CH_2_), 1.47 (d, *J* = 7.2 Hz, 2H, CH_2_), 1.25 (d, *J* = 13.7 Hz, 10H, 6*CH_2_). ^13^C NMR (100 MHz, DMSO-*d*_6_, δ): 192.5 (C=S), 174.4 (C=O), 166.1 (C=O), 154.1 (furan), 151.7 (furan), 130.8 (CH=), 128.4 (CH=), 127.7 (C-5), 127.5 (furan), 115.6 (CH=), 115.3 (furan), 44.1 (CH_2_), 33.6 (CH_2_), 28.8 (CH_2_), 28.7 (CH_2_), 28.6 (CH_2_), 28.5 (CH_2_), 28.4 (CH_2_), 26.1 (CH_2_), 26.0 (CH_2_), 24.4 (CH_2_). IR (KBr): 1703 and 1696 (C=O), 1577 and 1559 (C=C), 1507 and 1348 (N-O) cm^−1^. LCMS (ESI+) *m/z* 467.0 (100.0%, [M+H]^+^). Anal. calc. for C_21_H_26_N_2_O_6_S_2_: C, 54.06%; H, 5.62%; N, 6.00%. Found: C, 54.20%; H, 5.80%; N, 6.20%.

### 4.2. NCI Anticancer Screening In Vitro

Primary anticancer assay was performed on a panel of approximately sixty human tumor cell lines derived from nine neoplastic diseases, in accordance with the protocol of the Drug Evaluation Branch, National Cancer Institute, Bethesda. Tested compounds were added to the culture at a single concentration (10^−2^ M) and incubated for 48 h. The cytotoxic and/or growth inhibitory effects of each tested compound were evaluated using sulforhodamine B (SRB) assay. Results were reported as the percentage growth of the treated cells compared to the untreated control cells. The most active selected compounds were tested in vitro against the full panel of human tumor cell lines at concentrations ranging from 10^−4^ to 10^−8^ M. Protocol of 48 h of drug exposure was followed. SRB assay was used to estimate cell viability and growth. Using absorbance measurements [time zero (*Tz*), control growth in the absence of drug (*C*), and test growth in the presence of drug (*Ti*)], the percentage growth was calculated for each drug concentration. Percentage growth inhibition was calculated as

$$\left(\frac{Ti-Tz}{C-Tz}\right)\times 100$$

for concentrations for which *Ti* ≥ *Tz*, and

$$\left(\frac{Ti-Tz}{Tz}\right)\times 100$$

for concentrations for which *Ti* < *Tz*.

Dose response parameters (GI_50_, TGI) were calculated for each compound. Growth inhibition of 50% (GI_50_) was calculated from [(*Ti* − *Tz*)/(*C* − *Tz*)] × 100 = 50, which is the drug concentration resulting in a 50% lower net protein increase in the treated cells (measured by SRB staining) as compared to the net protein increase seen in the control cells. The drug concentration resulting in total growth inhibition (TGI) was calculated from *Ti* = *Tz*. Values were calculated for each of these parameters if the level of activity was reached; however, if the effect was not reached or was excessive, the value for that parameter was expressed as more or less than the maximum or minimum concentration tested. The lowest values were obtained with the most sensitive cell lines. Compounds having GI_50_ values ≤ 100 μM were declared to be active.

### 4.3. Cell Culture

Human breast cancer (MDA-MB-231, MCF-7, HCC 1954 CRL-2338), human gastric cancer (AGS CRL-1739), human colorectal adenocarcinoma (HT-29 HTB-38, DLD-1 CCL-221, HCT 116 CCL-247), human neuroblastoma (SH SY5Y CRL-2266), and human leukemia (JURKAT E6-1 TIB-152, RMPI 8866, K-562 CCL-243) cell lines were originally obtained from the American Type Culture Collection (ATCC, Manassas, VA, USA). The cells were cultured in a cell culture medium: DMEM (Corning, Kennebunk, ME, USA): MDA-MB-231, MCF-7, K562, SH SY5Y and AGS, RPMI-1640 (ATCC, Manassas, VA, USA): HCC 1954, JURKAT, RPMI8866 and DLD-1, McCoy’s 5A (PAN-Biotech, Aidenbach, Germany): HT-29 HTB-38, HCT-116 CCL-247. The medium was supplemented with 10% fetal bovine serum—FBS (Gibco, Grand Island, NY, USA)—and 1% antimicrobial substances of penicillin-streptomycin (Corning, Kennebunk, ME, USA). Cells were cultured in the tissue culture dishes (Sarstedt, Numbrecht, Germany) and they were kept at 37 °C in an atmosphere containing 5% of CO_2_, with the humidity between 90 and 95% until they reached 85–90% confluence. To detach cells, 0.05% trypsin containing 0.02% EDTA (Corning, Kennebunk, ME, USA) and phosphate-buffered saline (PBS) without calcium and magnesium (Corning, Kennebunk, ME, USA) were used. Cells were counted using a Scepter 3.0 cell counter (Merc Milipore, Burlington, MA, USA) prior to seeding into plates. The 6-well cell culture plates used 5 × 10^5^ cells/well in 3 mL of growth medium, the 24-well cell culture plates were seeded with 5 × 10^4^ cells/well in 1 mL of medium, while the 96-well plates (all plates from Sarstedt, Numbrecht, Germany) used 1 × 10^4^ cells/well in 100 μL of medium. Cells that reached approximately 80% confluence were used for further analysis.

### 4.4. Cell Viability Assay

The selected cell lines were seeded into 96-well plates and cultured in accordance with the previously described methodology. Then, the cells were treated with all tested compounds and DOX at concentrations of 0.625, 1.25, 2.5, 5.0, 10.0, 20.0, and 40.0 μM for 24 h. A solution of 3-(4,5-dimethyl-2-thiazolyl)-2,5-diphenyl-2*H*-tetrazolium bromide (Sigma-Aldrich, St. Louis, MO, USA) in phosphate-buffered saline without calcium and magnesium (Corning, Kennebunk, ME, USA) at a concentration of 5 mg/mL was used to carry out MTT assay. After incubation, 20 μL of the prepared MTT solution was added, and then the cells were incubated at 37 °C for two hours. Afterwards, the cells were lysed using 100 μL of lysing buffer (comprising 25 mM HCl, 2% acetic acid, 3% DMF, and 5% SDS, with a pH of 4.7). The absorbance was determined at a wavelength of 570 nm using an Absorbance 96 microplate reader (Byonoy GmbH, Hamburg, Germany).

### 4.5. [^3^H]-Thymidine Incorporation Assay

Following the selection of cell lines for further study, the effects of all compounds on cell proliferation were evaluated on MCF-7 and MDA-MB-231 cell lines. Cells were seeded in 24-well plates and cultured as described above. Cells were treated with the compounds at concentrations 1, 5, and 10 μM, and 5, 15, and 30 μM for MCF-7 and MDA-MB-231 cell lines, respectively, for 24 h. Subsequently, 0.5 μCi [^3^H]-thymidine was added for 4 h at 37 °C. Then, plates with cells were placed on the ice, the liquid was aspirated, and cells were washed twice with 1mL of 0.05 M Tris-HCl buffer comprising 0.11 M NaCl and precipitated by adding 1 mL per well of 5% trichloroacetic. After precipitation, the cells were dissolved with 1 mL of 0.1 M NaOH with 1% SDS (Sigma-Aldrich, St. Louis, MO, USA) at room temperature (RT). The cell lysates were transferred into scintillation vials containing 2 mL of scintillation fluid (Ultima Gold™ XR, PerkinElmer, MA, USA), and cell-associated radioactivity was measured using a Scintillation Counter 1900 TR, TRI-CARB (Packard, Perkin Elmer, Inc., San Jose, CA, USA). The [^3^H]-thymidine uptake was expressed as a percentage of the control value.

### 4.6. Flow Cytometry Assessment of Annexin V Binding

To conduct induction of apoptosis on MCF-7 and MDA-MB-231 cell lines, three compounds with the lowest IC_50_ values were selected (**2b**, **12b**, **14b**). The concentration of these compounds depended on the cell line. For MCF-7 cell line concentrations of 1 and 5 μM were used, while for MDA-MB-231 cell line concentrations were 5 and 10 μM. For both cell lines doxorubicin was used as a reference compound in 1 and 5 μM concentrations. This study was evaluated using a flow cytometer (BD FACSCanto II, Becton Dickinson Biosciences Systems, San Jose, CA, USA) and Annexin V binding Apoptosis Detection Kit II (BD Biosciences, San Diego, CA, USA). Cells were seeded in 6-well culture plates cultured in accordance with the previously described methodology. The medium was removed from wells and then cells were washed with PBS. A cell suspension was prepared in the binding buffer provided in the kit at a concentration of 1 × 10^6^ cells/mL. A total of 100 µL of cell suspension was collected from each sample and transferred to the tubes. Afterwards, 5 µL of Annexin V-FITC and 4 µL of propidium iodide (PI) were added to each tube and then the tubes were incubated in the dark for 15 min at room temperature. After incubation, the suspension was supplemented to 300 µL with a binding buffer and immediately read in a BD FACSCanto II flow cytometer (Becton Dickinson Biosciences, San Jose, CA, USA), where 10,000 events per sample were measured. Results were analyzed using FACSDiva Software Version 6.1.3. (BD Biosciences Systems, San Jose, CA, USA).

### 4.7. Mitochondrial Membrane Potential (ΔΨ_m_) Analysis

The mitochondrial membrane potential (ΔΨ_m_) was analyzed by using the JC-1 MitoScreen kit (BD Biosciences, San Diego, CA, USA). The tested compounds and reference drug were used in 1 and 5 µM concentrations on the MCF-7 cell line and 5 and 10 µM on the MDA-MB-231 cell line. After 24 h of incubation with the tested compounds, the assay was performed as described in the literature [4]. The assay was performed using a flow cytometer (BD FACSCanto II, Becton Dickinson Biosciences Systems, San Jose, CA, USA), and results were obtained using FACSDiva software (version 6.1.3, BD Biosciences Systems, San Jose, CA, USA).

### 4.8. Caspase-9 Activity Assessment

To determine the effects of the novel compounds on the caspase-9 activity, the FLICA Caspase-9 Assay Kit was applied (ImmunoChemistry Technologies, Bloomington, MN, USA). MCF-7 and MDA-MB-231 cells were seeded on 6-well plates (5 × 10^5^ cells per well) and incubated for 24 h. Next, they were treated with tested compounds **2b**, **12b**, and **14b** in a concentration of 1 and 5 µM for the MCF-7 cell line and 5 and 10 µM for the MDA-MB-231 cell line and the reference drug, doxorubicin hydrochloride, in a concentration of 1 and 5 µM for both lines for 24 h. Then, cells were harvested, washed with PBS, and resuspended in an Apoptosis Wash Buffer to a final concentration of 5 × 10^5^ cells/mL. The following step was to prepare 290 μL of cell suspension and transfer it into the tubes. Afterwards, 10 μL of FLICA solution was introduced to the cell suspension and incubated at 37 °C for half an hour. Next, using 2 mL of Apoptosis Wash Buffer, cells were washed two times, centrifuged, and resuspended in 300 µL of the buffer. Prepared samples were evaluated by a BD FACSCanto II flow cytometer (Becton Dickinson Biosciences, San Jose, CA, USA). For each sample, 1 × 10^4^ events were analyzed. The obtained results were investigated by applying FACSDiva software Version 6.1.3 (BD Biosciences Systems, San Jose, CA, USA).

### 4.9. Determination of Cytochrome C and Caspase-3 Concentrations

Using a SimpleStep ELISA kit (Abcam, Cambridge, UK), we determined the levels of cytochrome C and caspase-3 in cells’ lysates with **2b**, **12b**, and **14b** (1 and 5 μM for the MCF-7 cell line and 5 and 10 μM for the MDA-MB-231) and doxorubicin in concentrations of 1 and 5 μM for both lines after 24 h incubation. Assays were performed according to the manufacturer’s protocols.

### 4.10. Bax Concentration Assessment

An anti-Bax FITC-conjugated antibody was applied to evaluate the effect of tested compounds on the concentration of Bax protein in this experiment. First, MCF-7 and MDA-MB-231 cells were seeded into 6-well plates at 5 × 10^5^ cells per well. Test compounds **2b**, **12b**, and **14b** were then added at concentrations of 1 and 5 µM and 5 and 10 µM for MCF-7 and MDA-MB-231, respectively. The concentration of doxorubicin as a reference compound was 1 and 5 µM for both lines. After 24 h of incubation, cells were harvested, centrifuged, washed with 1 mL of PBS, and then fixed with 4% paraformaldehyde for 15 min at RT. After removal of the paraformaldehyde, cells were washed with PBS, then resuspended in 0.5 mL of cold 90% methanol, and frozen at −20 °C. After overnight incubation, the methanol was discarded, the cells were washed three times with PBS, and then the antibody in stain buffer was added according to the protocol (1 µL of antibody per 100 µL of buffer). The samples were incubated in the dark at RT, then centrifuged and resuspended in 0.3 mL of stain buffer. The prepared samples were read immediately using the BD FACS Canto II flow cytometer (Becton Dickinson Biosciences, San Jose, CA, USA).

### 4.11. Reactive Oxygen Species Assessment

The influences of **2b**, **12b**, **14b**, and doxorubicin (1 and 5 µM and 5 and 10 µM for MCF-7 and MDA-MB-231 cell lines, respectively) on the generation of reactive oxygen species (ROS) were analyzed. Cells were seeded in 6-well culture plates at 1 × 10^5^ cells per well in 1 mL of medium and then treated with compounds for 1 h and then washed with PBS. Double staining was conducted with 20 µM 2′,7′-dichlorodihydrofluorescein diacetate (H_2_DCFDA) reagent (Thermo Fisher Scientific, Eugene, OR, USA), which is colorless, and is cleaved by intracellular esterases and oxidized to the highly fluorescent 2′,7′-dichlorofluorescein (DCF), and 10 µg/mL of the fluorescent dye 4′,6-diamidino-2-phenylindole (DAPI) (Sigma-Aldrich, St. Louis, MO, USA), which visualizes the nuclear DNA of cells. A Nikon ECLIPSE Ti fluorescence microscope (Nikon, Tokyo, Japan) was used to obtain results which were analyzed using ImageJ software 1.54k (Bethesda, MD, USA). The fluorescence intensity of the DCF reagent was evaluated relative to the control. The number of analyzed cells was above 100.

## 5. Conclusions

A series of 4-thiazolidinone hybrids with a 5-nitrofuranpropenylidene moiety were designed and synthesized as potential anticancer agents. The use of the 5-(5-nitrofuran-2-yl)allylidene moiety as a bioisosteric to the 5-(2-chloro-3-(4-nitrophenyl))allylidene (Ciminalum) pharmacophore was proposed. In accordance with obtained MTT assay results, the three most active compounds, **2b**, **12b**, and **14b**, were selected from fifteen and subjected to a series of subsequent biological studies. The study demonstrated the compounds’ effect on the intrinsic apoptotic pathway by observing increased concentrations of proapoptotic proteins such as Bax and cytochrome C. The activation of the mitochondrial apoptotic pathway cascade happened as a consequence of the significant increase in generation of reactive oxygen species by the tested compounds. Consequently, in addition to the increased concentrations of these proapoptotic proteins, a decrease in mitochondrial membrane potential and activation of caspases-9 and -3 were observed as well, which ultimately led to apoptosis in the MCF-7 and MDA-MB-231 cell lines. The results of DNA biosynthesis assay suggested the influence of compounds **2b**, **12b**, and **14b** on the structure of the cell nucleus; however, further research is required for more significant understanding of the mechanism of this activity. Additionally, checking the safety profile of the tested compounds and their metabolic stability would be valid given such high cytotoxic activity before including tested compounds in in vivo tests.

Displayed data provide a new advancement in regard to the medicinal chemistry of 4-thiazolidinone-bearing hybrid molecules as anticancer agents.

## Figures and Tables

**Table 1:** Obtained IC_50_ concentrations (μM) based on results of MTT assay after 24 h of treatment. The number of repetitions for each compound and for each line is 3.

Comp./ Cell Lines	1b	2b	3b	4b	5b	6b	7b	8b	9b	10b	11b	12b	13b	14b	15b	DOX
**Breast cancer**																
**MCF-7**	>40	11.32	7.74	5.87	3.32	6.39	7.13	2.38	14.1	9.23	33.13	3.01	1.63	0.85	4.05	0.73
**MDA-MB-231**	>40	25.36	18.51	17.46	9.12	10.35	16.7	8.34	36.72	22.9	>40	12.91	8.73	6.61	>40	7.53
**HCC-1954**	25.31	12.54	17.57	7.07	6.42	4.18	6.28	4.9	20.57	19.13	>40	4.86	3.12	3.3	6.23	3.14
**Gastric cancer**																
**AGS**	36.23	12.05	6.53	6.54	2.65	3.99	5.55	3.38	12.93	8.57	30.56	3.65	2.1	2.57	1.71	1.09
**Colorectal cancer**																
**DLD-1**	28.77	9.98	8.03	7.61	3.86	4.58	7.67	3.68	12.33	9.29	20.28	3.31	3.18	1.8	1.76	2.83
**HCT-116**	>40	38.99	14.65	7.89	4.21	17.55	7.98	4.15	11.69	13.48	36.09	3.68	3.97	2.11	10.53	16.76
**HT-29**	22.31	24.47	30.4	13.23	7.74	8.01	13.61	8.04	19.99	28.63	>40	8.56	4.39	4.52	9.61	0.73
**Neuroblastoma cell line**																
**SH SY5Y**	>40	30.63	28.54	16.32	9.37	8.39	15.55	6.6	20.11	18.89	>40	13.13	8.41	4.11	3.76	39.92
**Leukemia**																
**K562**	>40	>40	25.80	16.12	16.14	36.18	18.51	7.35	37.56	25.5	>40	7.15	3.57	1.53	15.54	0.43
**Jurkat**	1.76	4.78	14.15	5.85	3.73	13.94	7.72	3.19	2.51	6.7	17.09	1.77	0.5	0.28	1.41	0.72
**RPMI 8866**	>40	28.99	35.18	19.57	13.52	15.23	16.81	14.15	5.54	16.46	27.76	7.78	3.94	3.13	10.95	0.38

**Table 2:** Obtained IC_50_ concentrations (μM) based on results of [^3^H]-thymidine incorporation assay after 24 h of treatment. The number of repetitions for each compound and for each line is 3.

Comp./Cell Lines	1b	2b	3b	4b	5b	6b	7b	8b	9b	10b	11b	12b	13b	14b	15b	DOX
**Breast Cancer**																
**MCF-7**	>10	6.61	7.79	6.13	1.93	5.81	6.69	2.21	7.98	7.87	>10	6.66	3.97	2.6	3.13	1.78
**MDA-MB-231**	>30	5.56	2.65	3.66	6.01	2.01	2.19	1.84	7.53	5.99	>30	1.82	1.77	1.6	>30	0.36

## Data Availability

Data are contained within the article and Supplementary Materials.

## Supplementary Materials

The following supporting information can be downloaded at https://www.mdpi.com/article/10.3390/ph18111598/s1, Figures S1–S63: The spectral data of compounds **15a**, **1b**–**15b**; Table S1: NCI-60 anticancer screening data of compounds **4**–**6b**, **9b** and **12**–**14b** at 10 μM concentration on numerous cancer cell lines; Table S2: NCI-60 assessmnet of GI_50_/TGI/LC_50_ for compounds **4**–**6b**, **9b** and **12**–**14b** effects on individual tumor cell lines.

## Abbreviations

The following abbreviations are used in this manuscript:

ATP	Adenosine triphopshate
DAPI	4′,6-Diamidino-2-phenylindole
DCF	2′,7′-Dichlorofluorescein
DOX	Doxorubicin hydrochloride
DTP	Developmental Therapeutic Program
ELISA	Enzyme-linked immunosorbent assay
FBS	Fetal bovine serum
FDA	The Food and Drug Administration
GI_50_	Molar concentration of the compound that inhibits 50% net cell growth
IC_50_	Half maximal inhibitory concentration
LC_50_	Molar concentration of the compound that inhibits 50% net cell growth
MTT	(3-(4,5-Dimethylthiazol-2-yl)-2,5-diphenyltetrazolium bromide) assay
MP	Melting point
NCI	National Cancer Institute
PBS	Phosphate-buffered saline
PI	Propidium iodide
ROS	Reactive oxygen species
SAR	Structure-activity relationship analysis
SDS	Sodium dodecyl sulfate
SRB	Sulforhodamine B
TGI	Total growth inhibition
