# Supplementary material for: Synthesis and Anticancer Activity Evaluation of New 5-((5-Nitrofuran-2-yl)allylidene)-2-thioxo-4-thiazolidinones
Source: Pharmaceuticals (Basel). 2025 Oct 22;18(11):1598. doi: 10.3390/ph18111598 (PMC12655351; doi:10.3390/ph18111598)
Supplement: Supplementary file 1 [file pharmaceuticals-18-01598-s001.zip › pharmaceuticals-3867168-supplementary.pdf]

Supplementary information

Supplementary data: The spectral data of compounds **15a, 1b-15b**, NCI-60 anticancer screening data of compounds **4-6b**, **9b** and **12-14b**.

Table of Contents

Copies of ^{1}H, ^{13}C, NMR, IR and LC-MS spectra of compounds **15a, 1b-15b**..........Figures S1-S63

NCI-60 anticancer screening data of compounds **4-6b**, **9b** and **12-14b** at 10 μM concentration on numerous cancer cell lines..........Table S1

NCI-60 assessmnet of GI_{50}/TGI/LC_{50} for compounds **4-6b**, **9b** and **12-14b** effects on individual tumor cell lines.......... Table S2

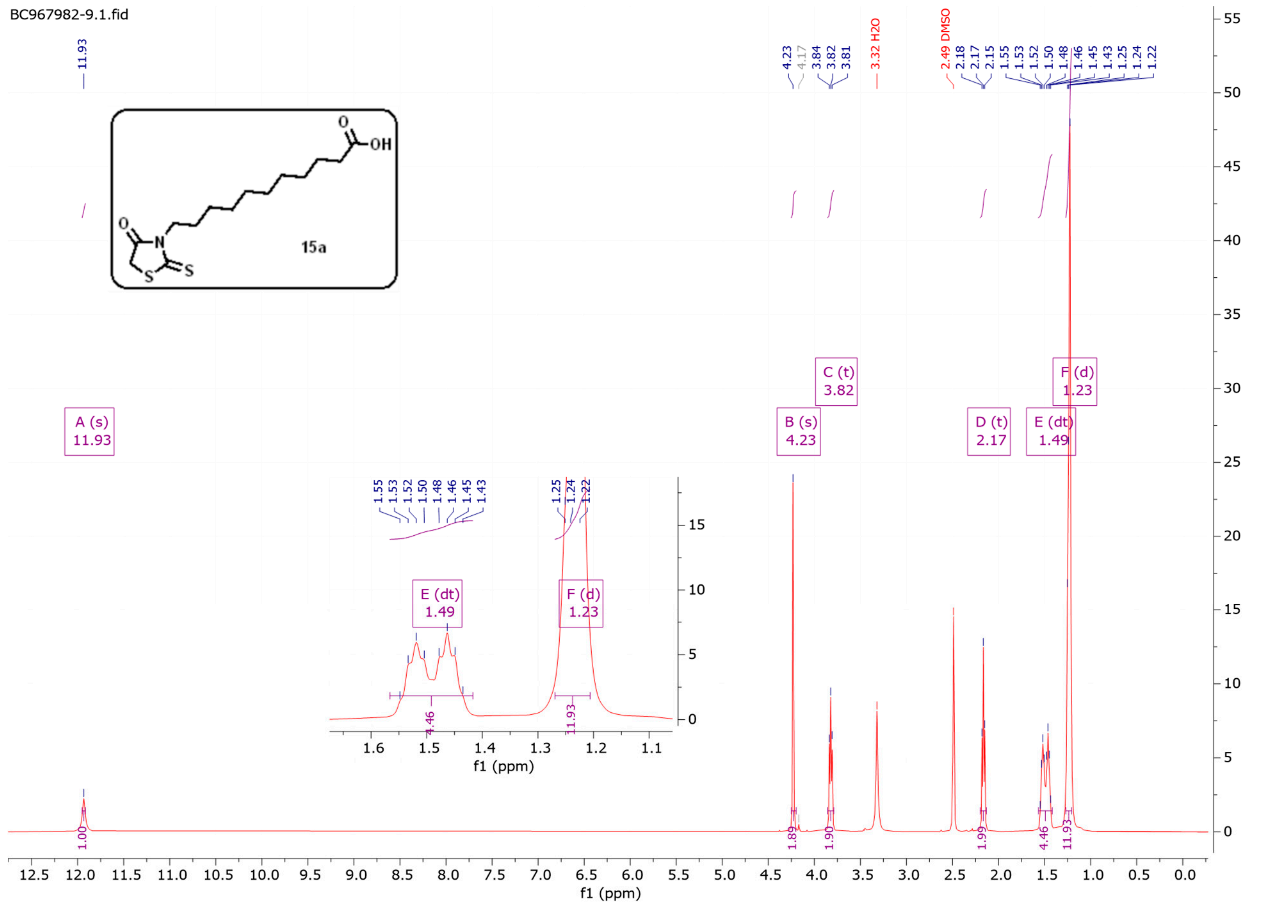

Figure S1. ^{1}H NMR spectrum of compound **15a**.

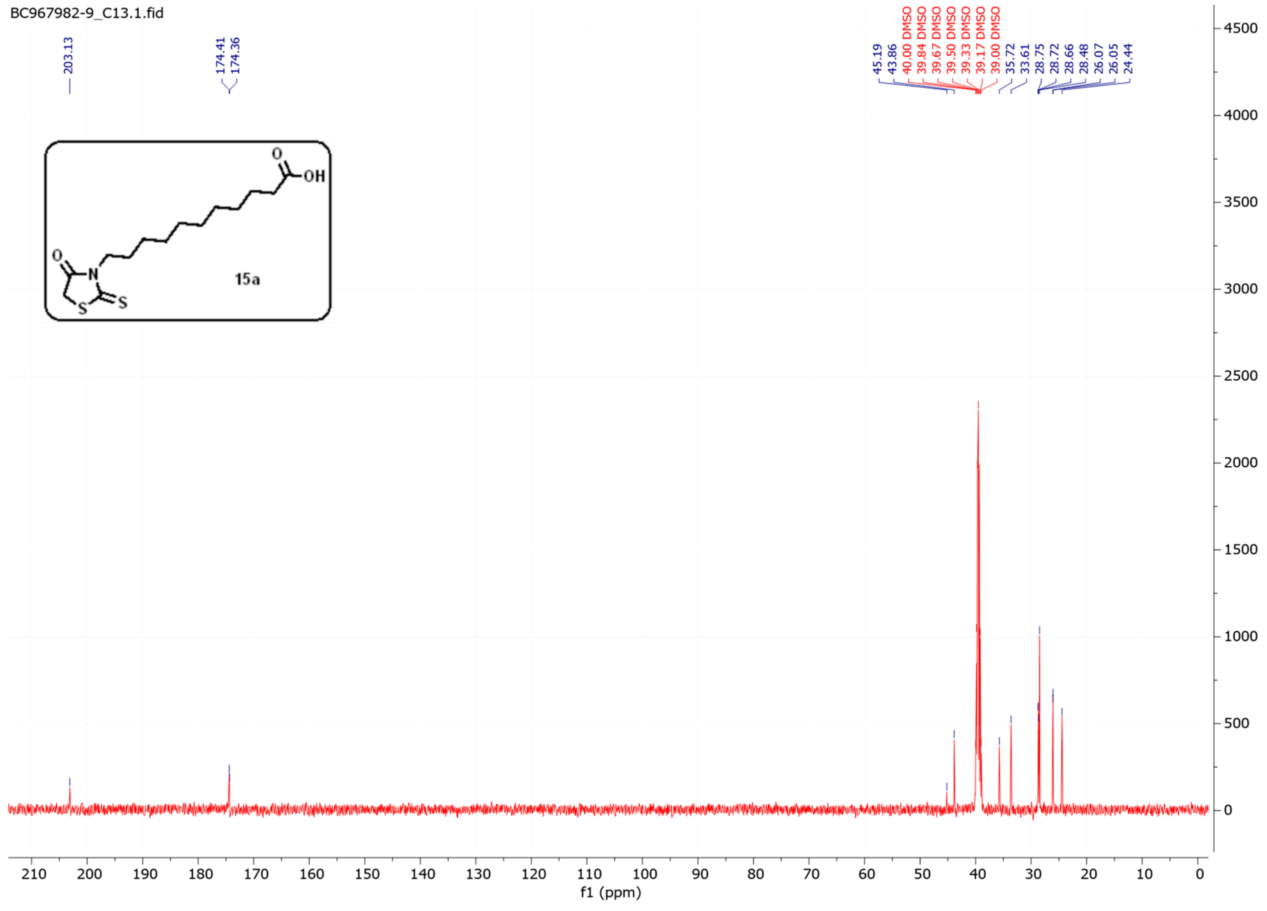

Figure S2. ^{13}C NMR spectrum of compound **15a**

#	RT	DAD1A	DAD1B	MSD1	MSD2	ELSD	MSD1 ions	MSD1 rt	MSD2 ions	MSD2 rt	Info
1	1.403	100.0%	100.0%	86.4%	100.0%	100.0%	300.0(60),340.0(25),318.0(15)	1.409	316.2(95),338.2(5)	1.412	
2	1.546	---	---	13.6%	---	---	485.4(74),501.2(26)	1.554	---	---	

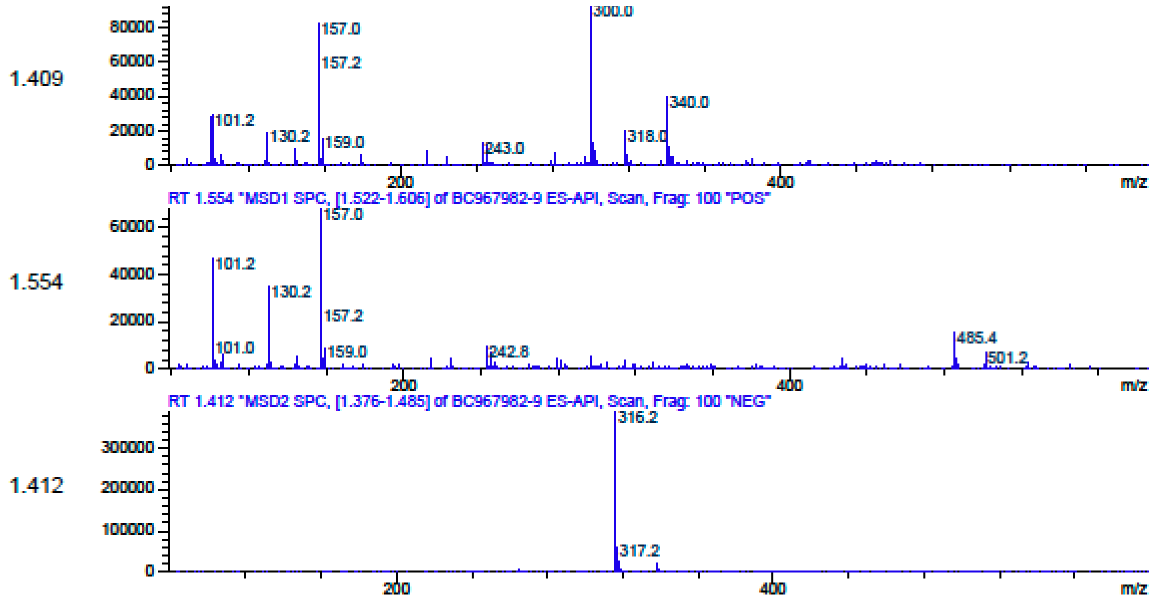

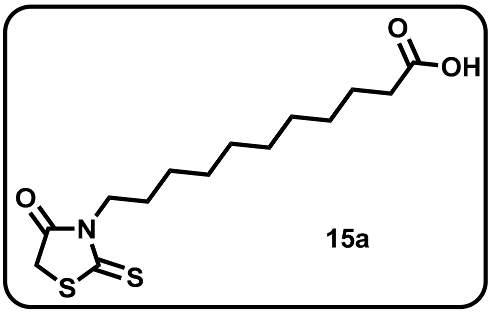

Molecular Weight: 317,46

Figure S3. LC-MS spectrum of compound **15a**.

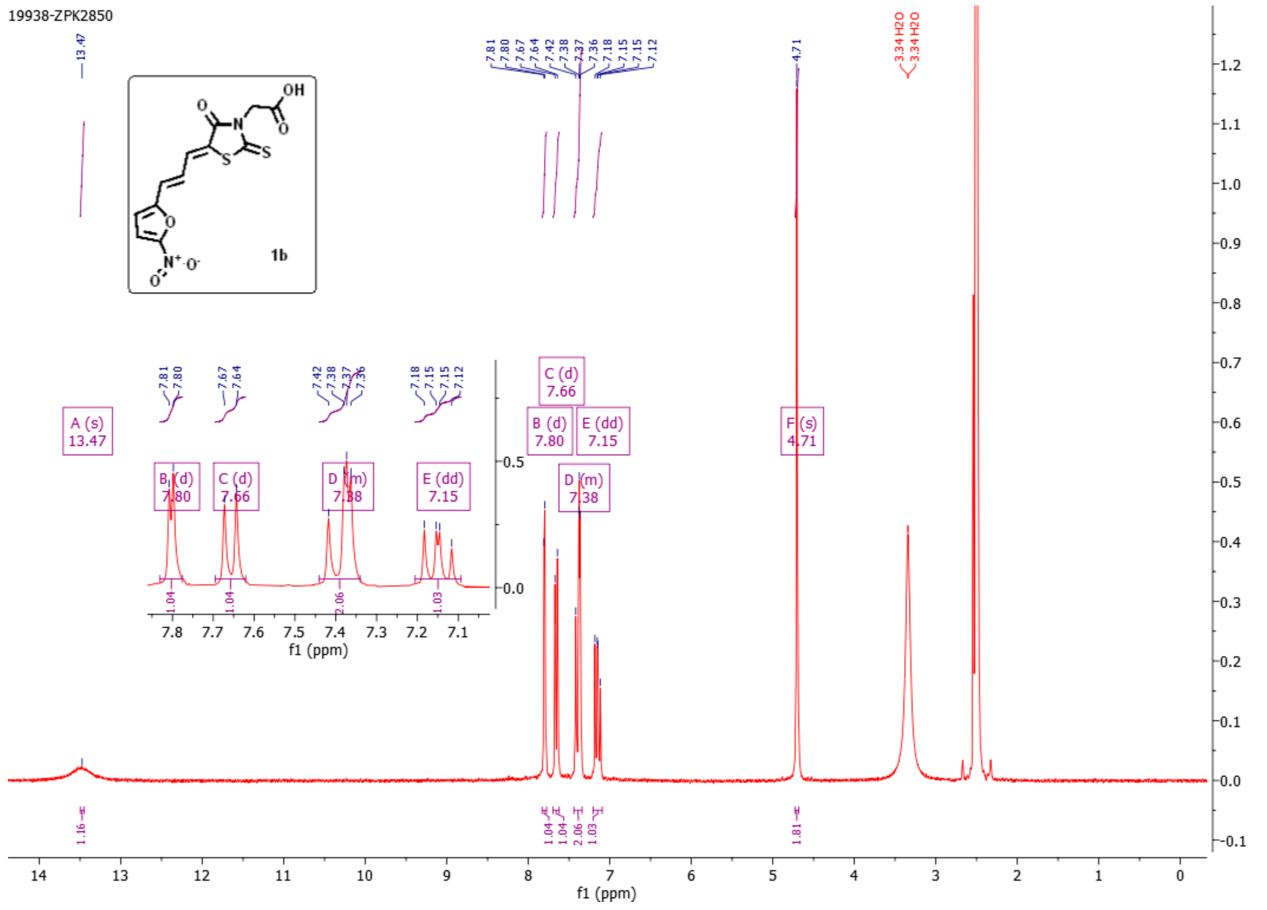

Figure S4. ^{1}H NMR spectrum of compound **1b**.

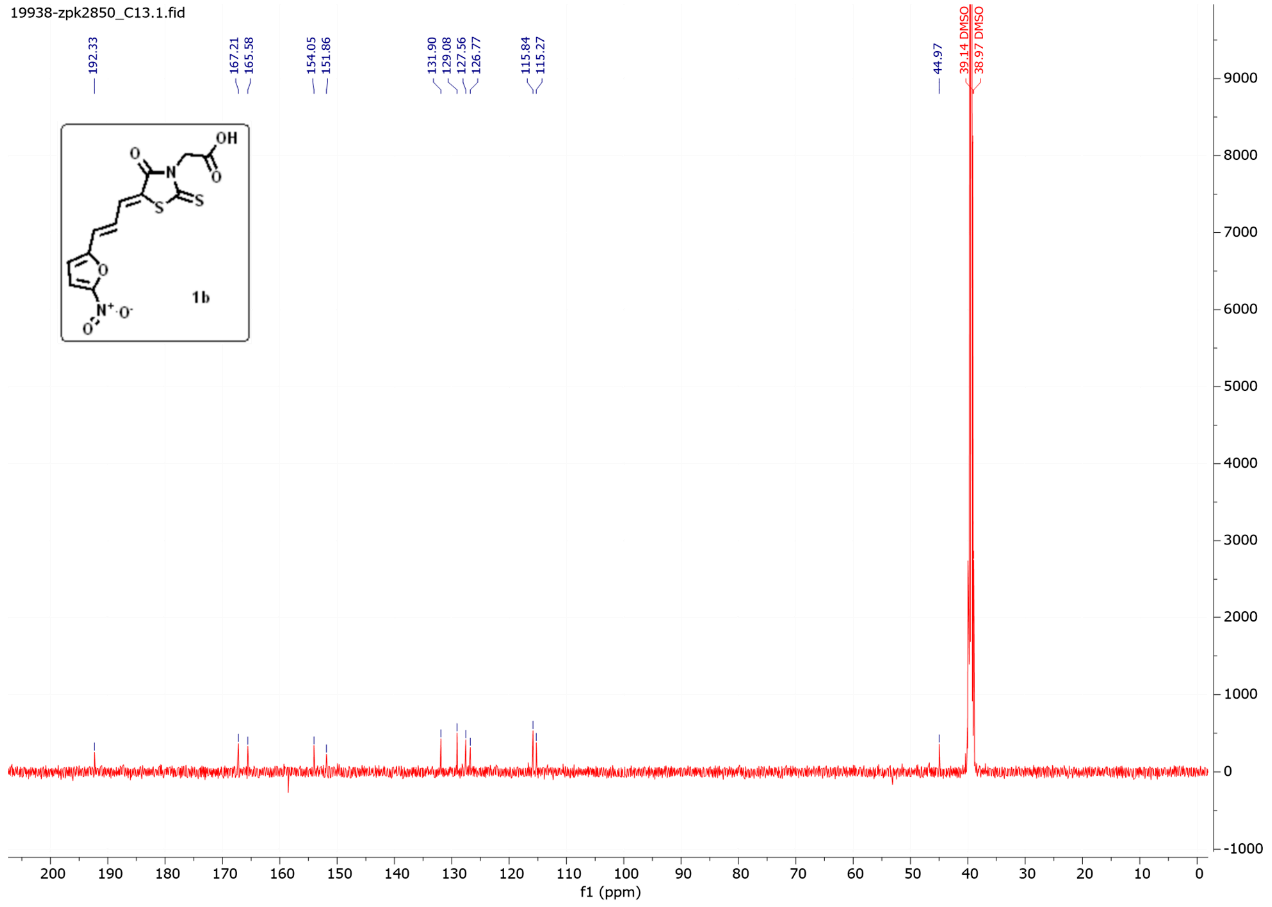

Figure S5. ^{13}C NMR spectrum of compound **1b**

#	RT	DAD1A	DAD1B	MSD1	MSD2	ELSD	MSD1 ions	MSD1 rt	MSD2 ions	MSD2 rt	Info
1	1.081	1.3%	1.6%	—	1.2%	—	—	—	207.8(100)	1.086	
2	1.268	98.7%	98.4%	100.0%	98.8%	89.4%	341.0(79),363.0(16),322.8(4)	1.275	240.0(32),297.0(25),264.8(16)	1.276	
3	1.823	—	—	—	—	10.6%	—	—	—	—	

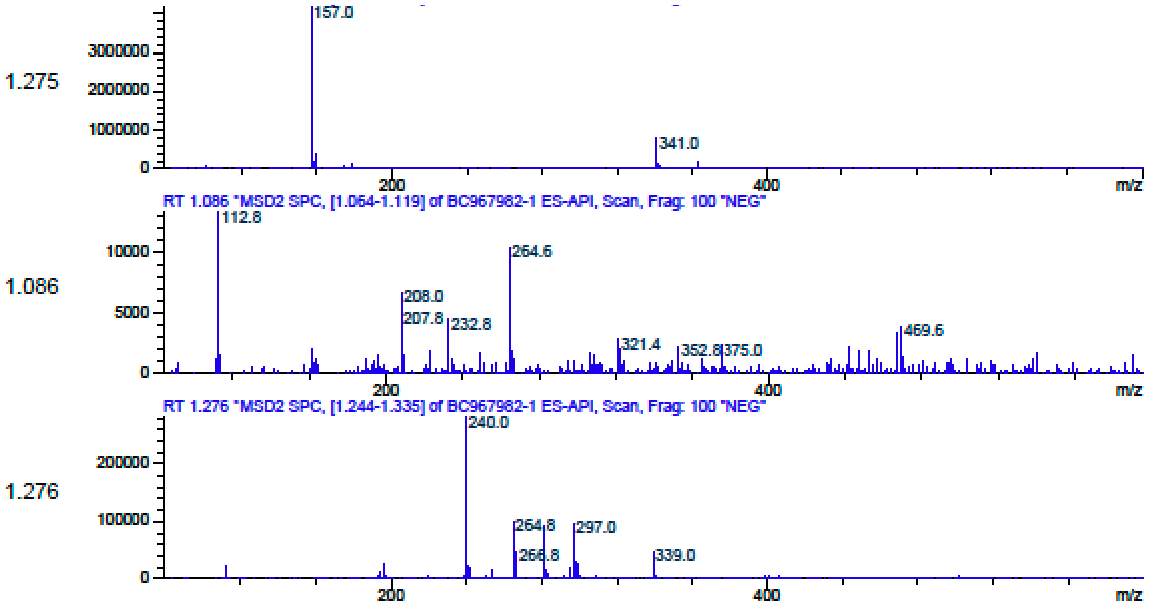

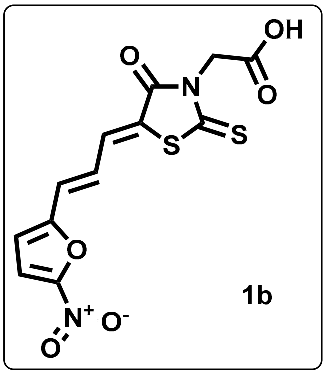

Molecular Weight: 340,32

Figure S6. LC-MS spectrum of compound **1b**.

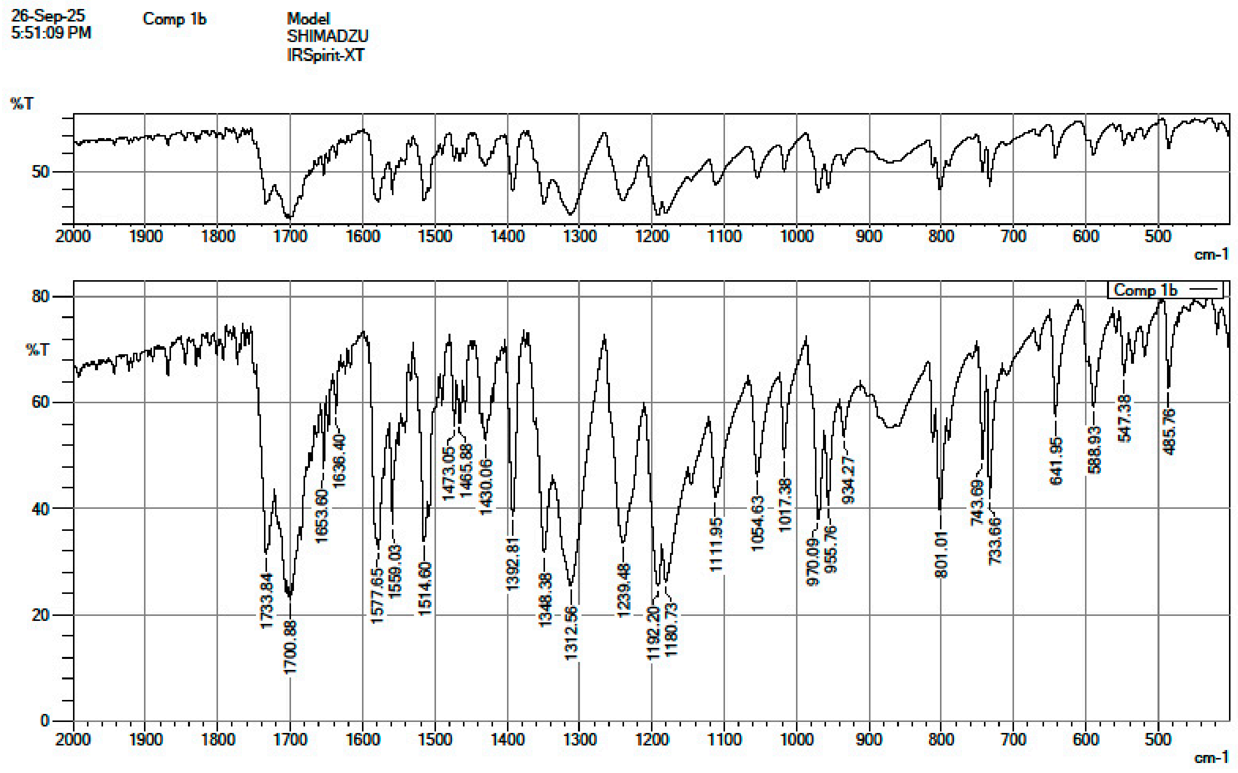

Figure S7. IR spectrum of compound **1b**.

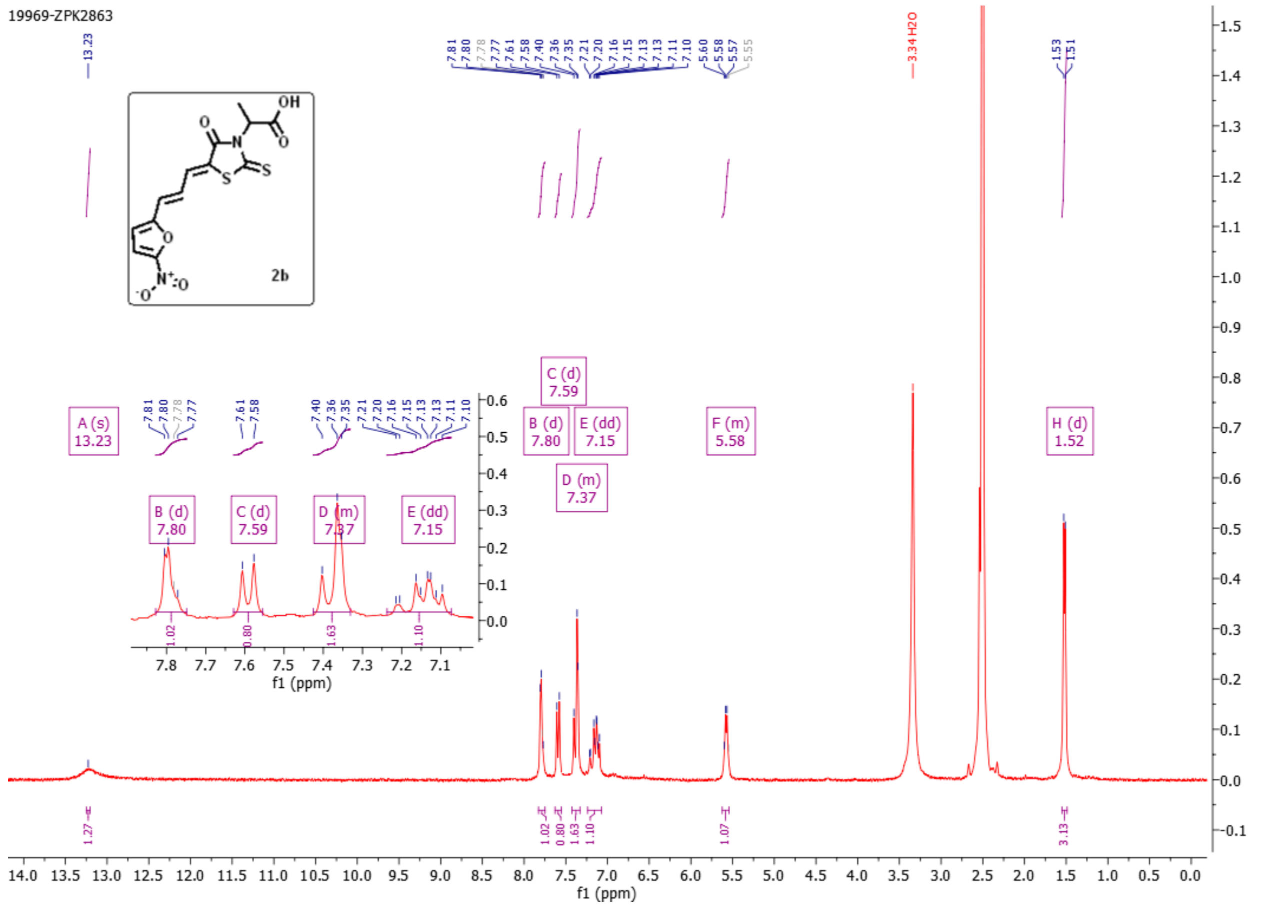

Figure S8. ^{1}H NMR spectrum of compound **2b**.

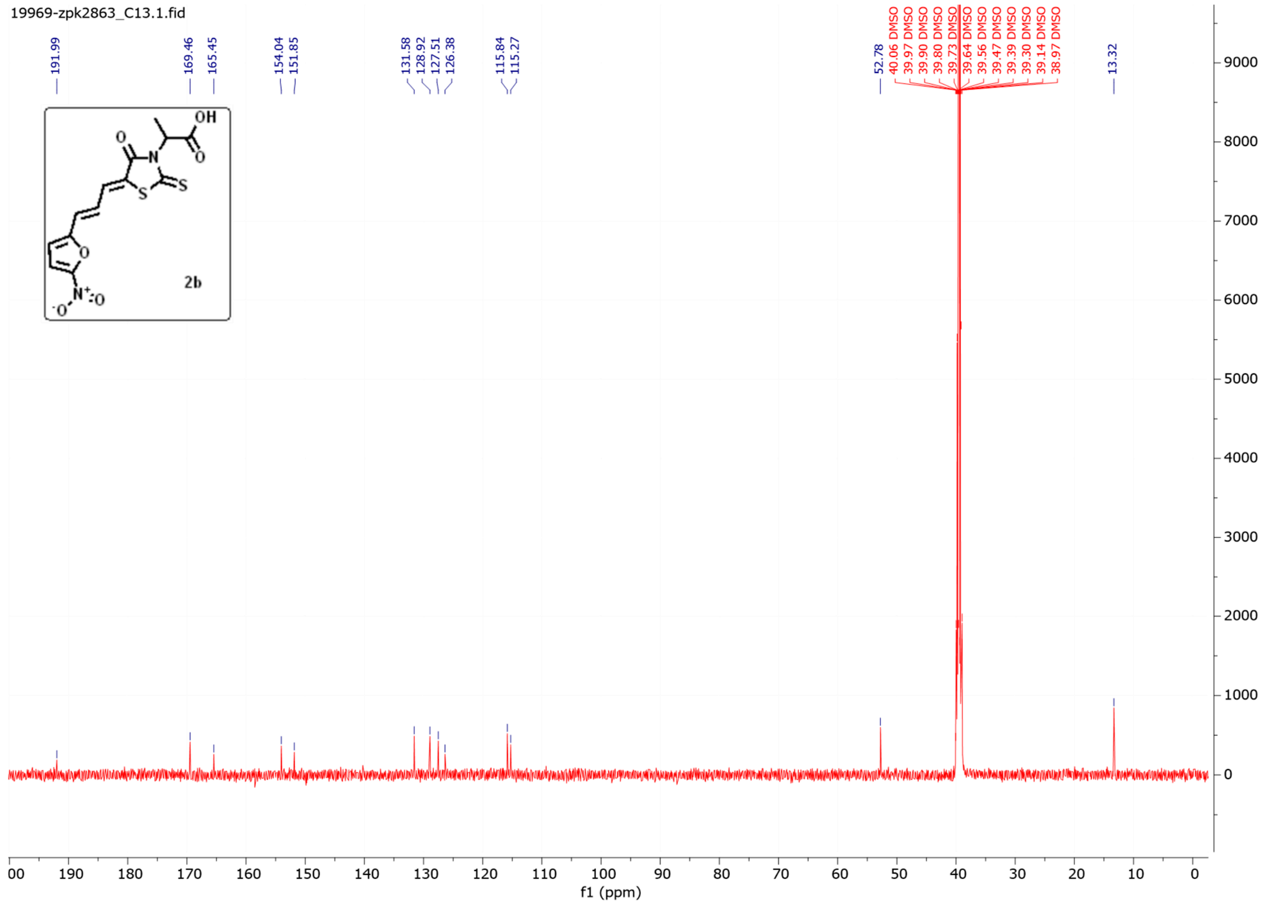

Figure S9. ^{13}C NMR spectrum of compound **2b**

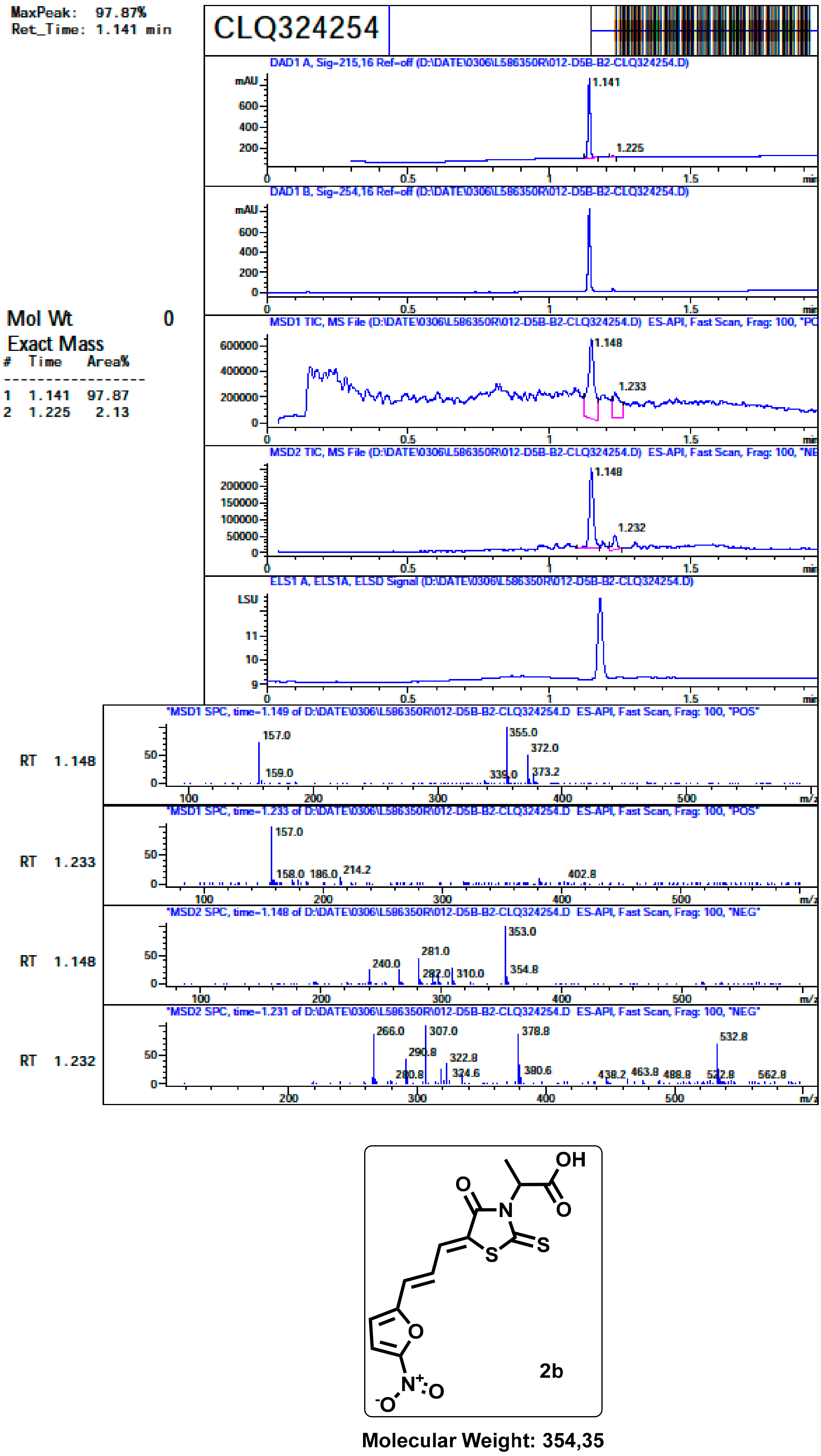

Figure S10. LC-MS spectrum of compound **2b**

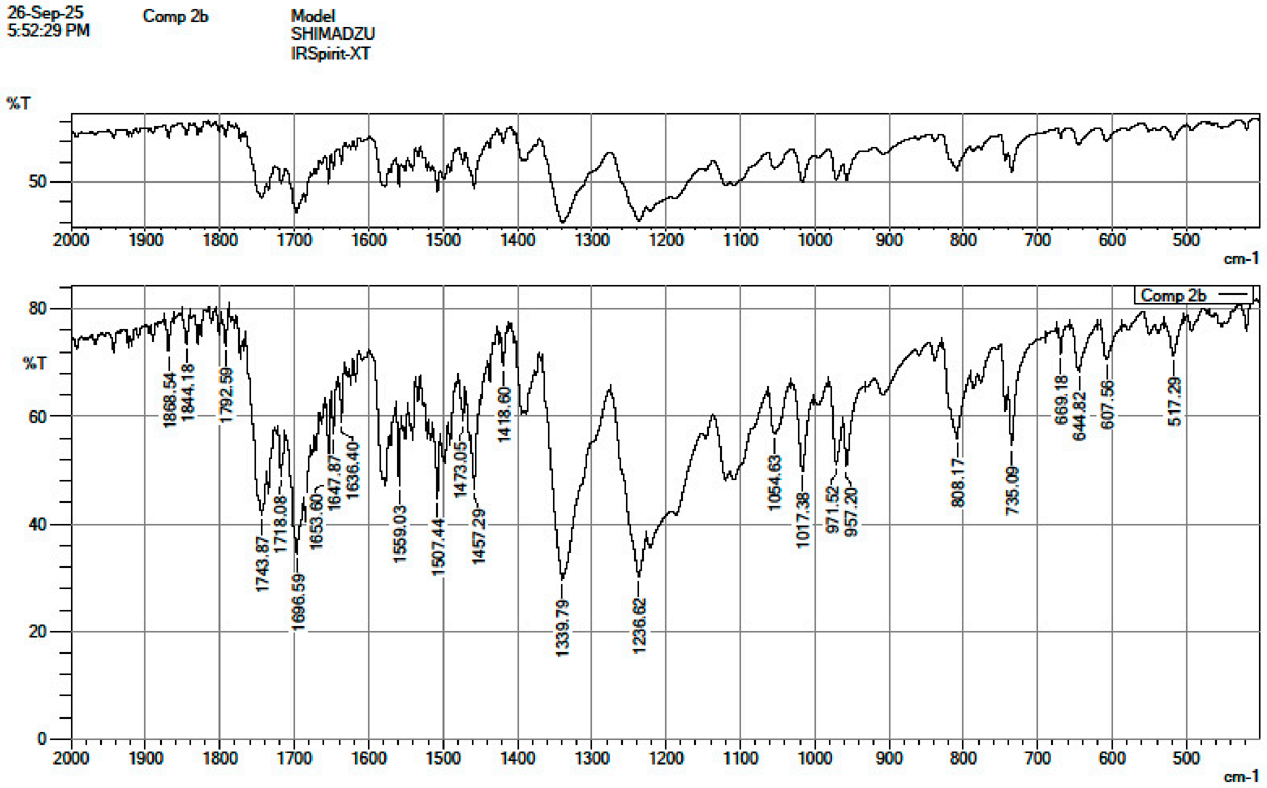

Figure S11. IR spectrum of compound **2b**

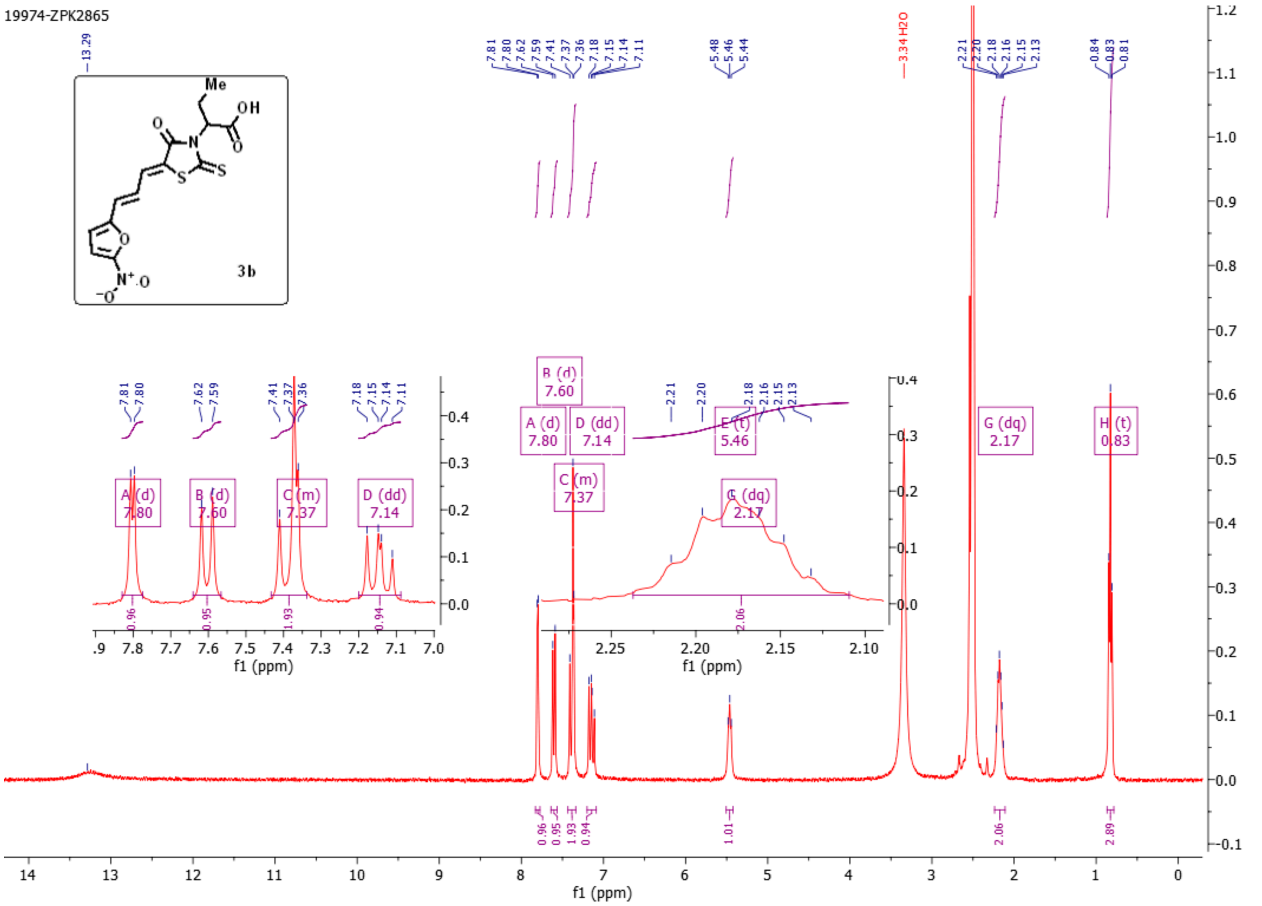

Figure S12. ^{1}H NMR spectrum of compound **3b**.

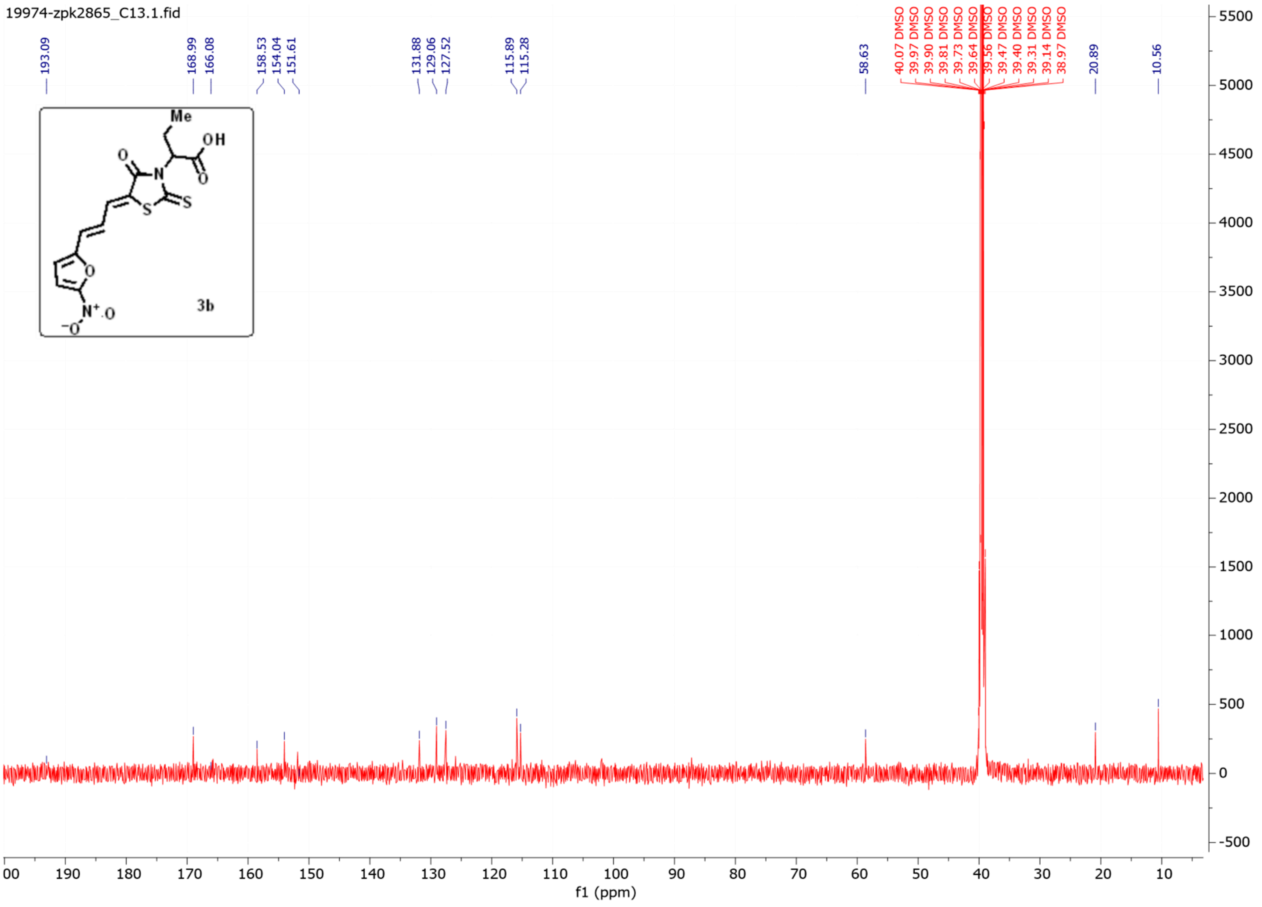

Figure S13. ^{13}C NMR spectrum of compound **3b**

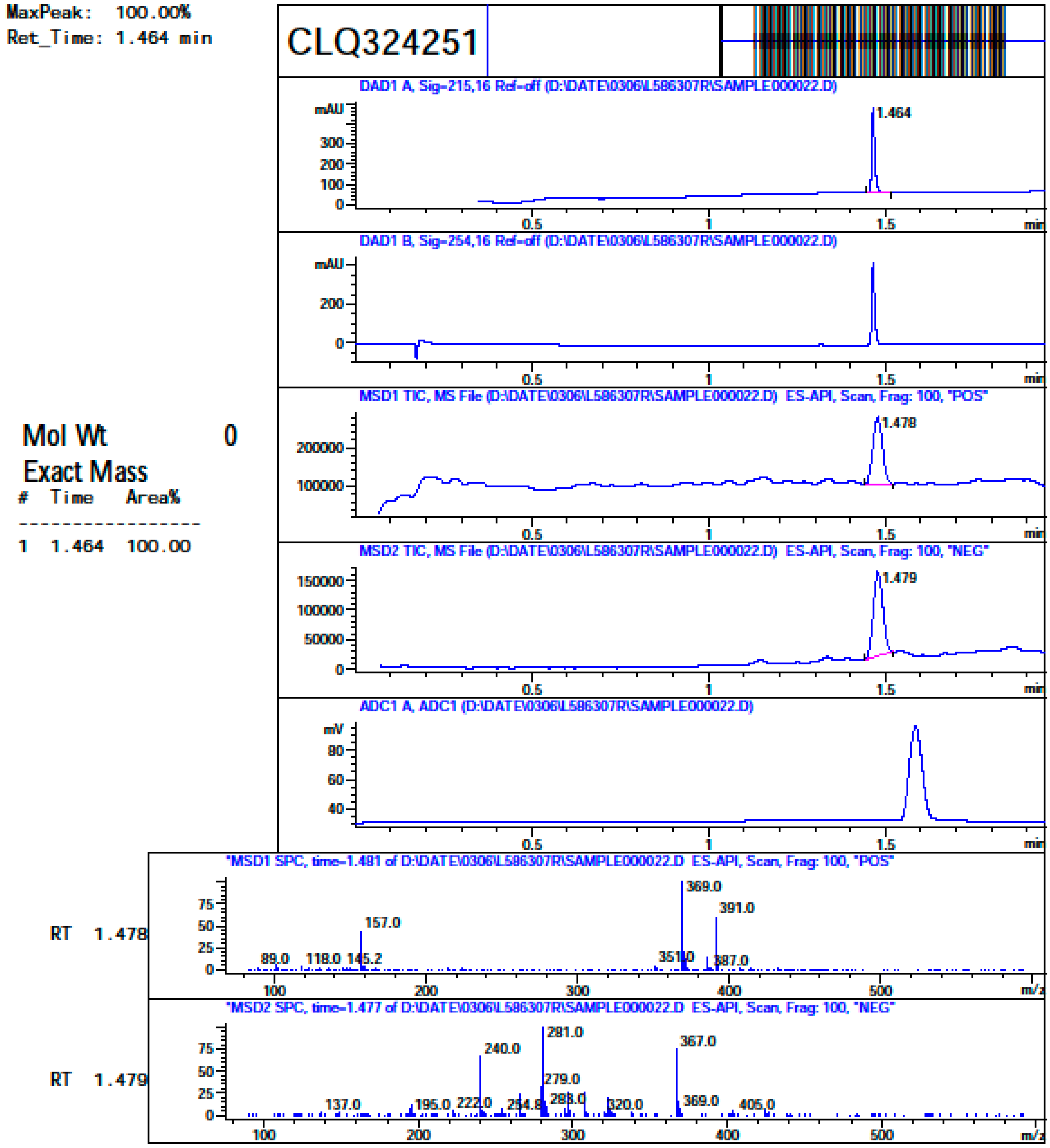

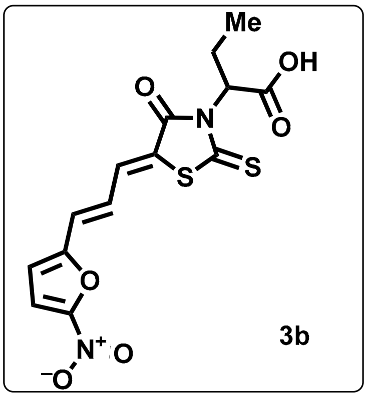

Molecular Weight: 368,38

Figure S14. LC-MS spectrum of compound **3b**

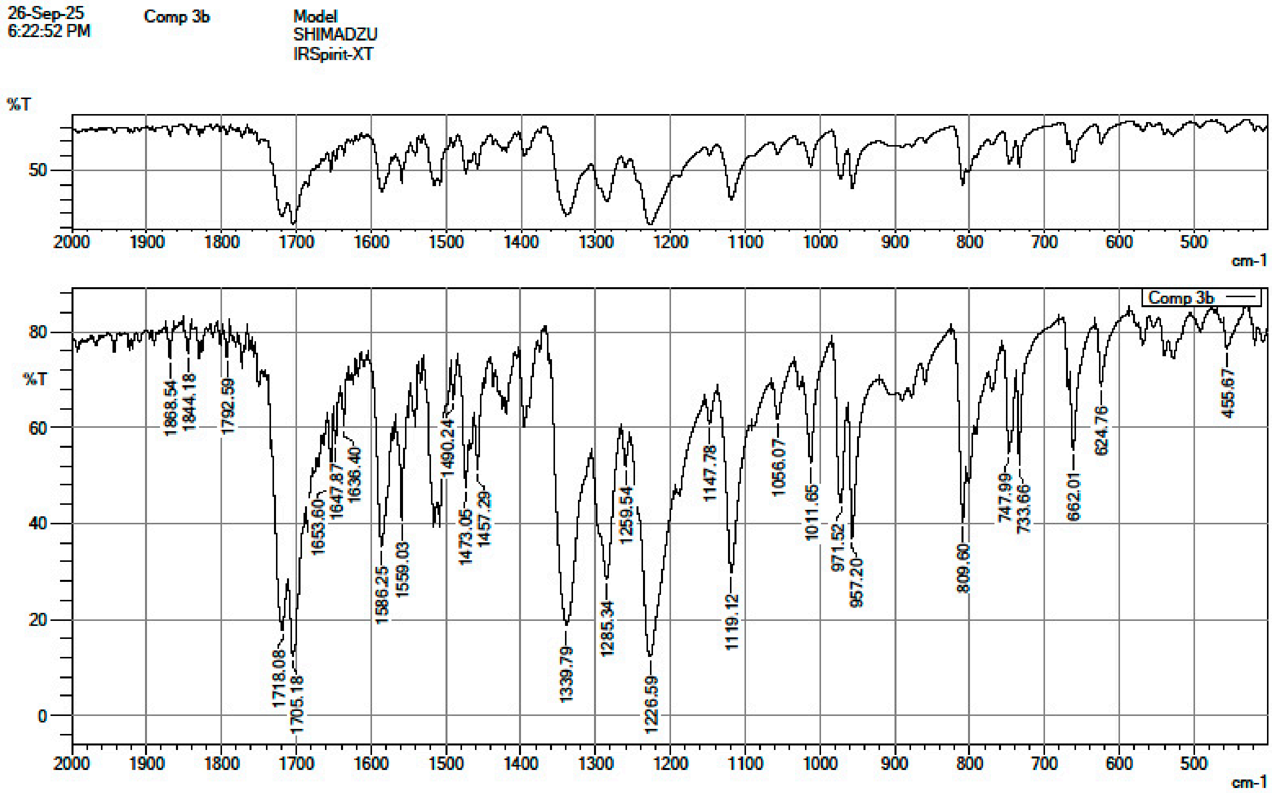

Figure S15. IR spectrum of compound **3b**

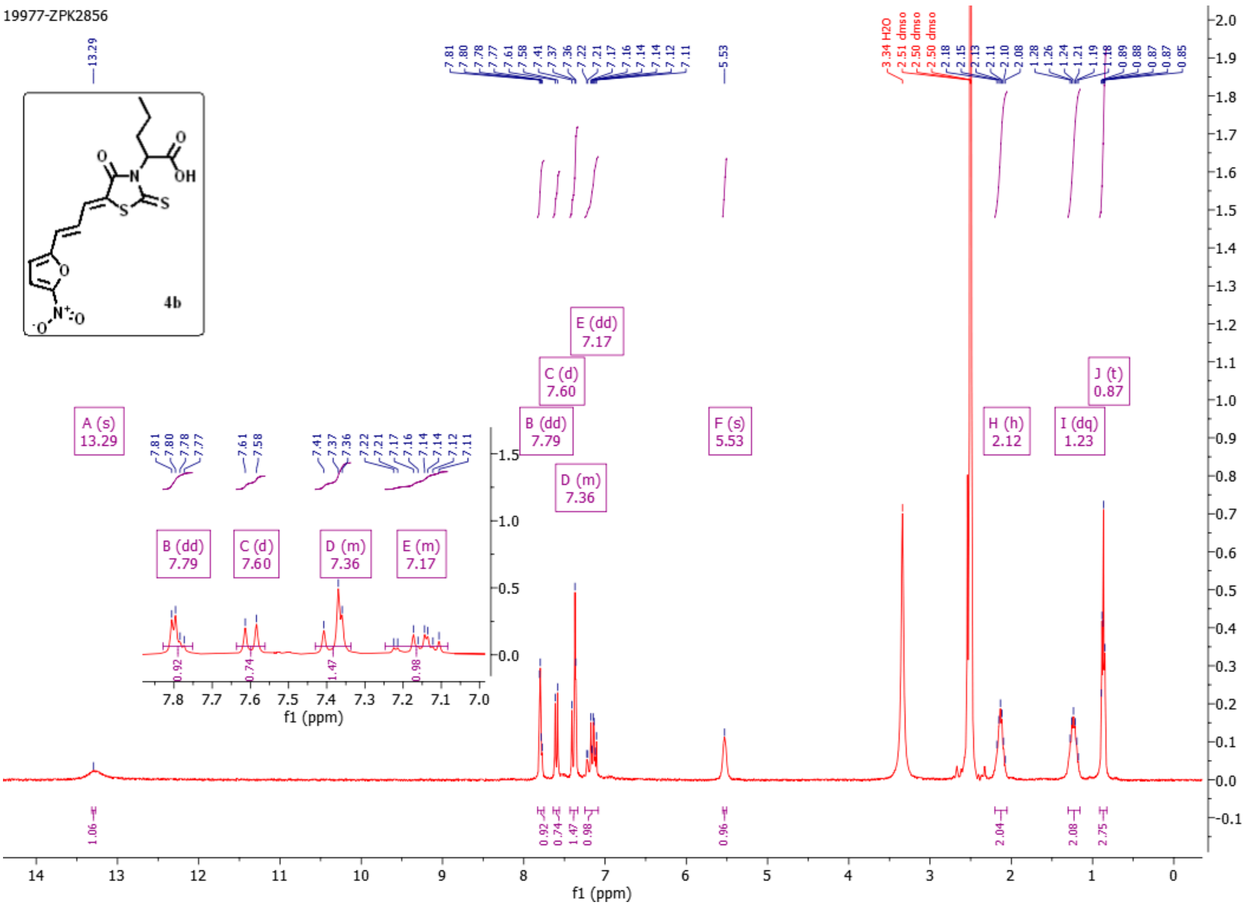

Figure S16. ^{1}H NMR spectrum of compound **4b**.

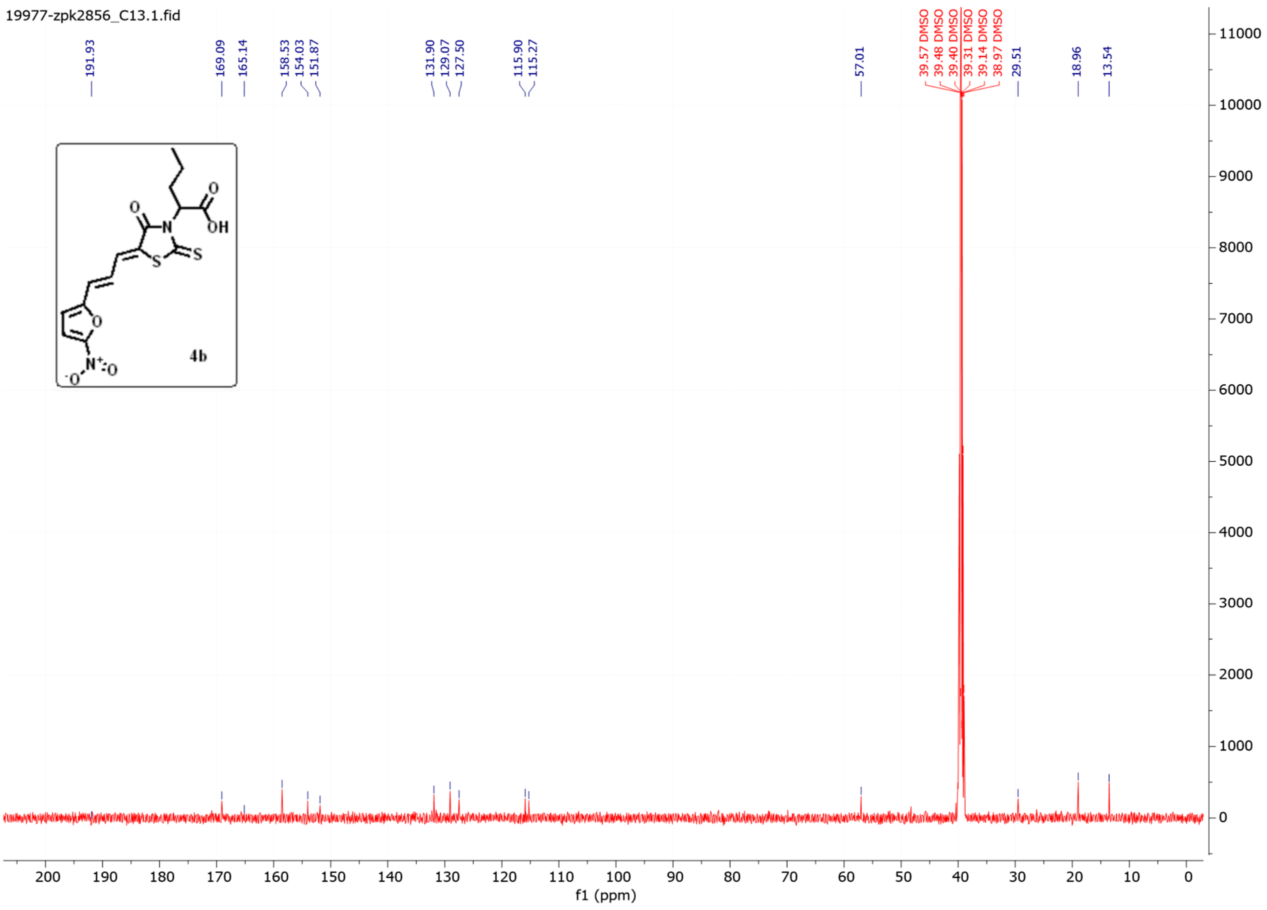

Figure S17. ^{13}C NMR spectrum of compound **4b**

#	RT	DAD1A	DAD1B	MSD1	MSD2	ELSD	MSD1 ions	MSD1 rt	MSD2 ions	MSD2 rt	Info
1	1.482	100.0%	100.0%	100.0%	100.0%	100.0%	383.0(71),405.0(14),365.0(13)	1.490	381.0(24),281.0(21),240.0(16)	1.490	

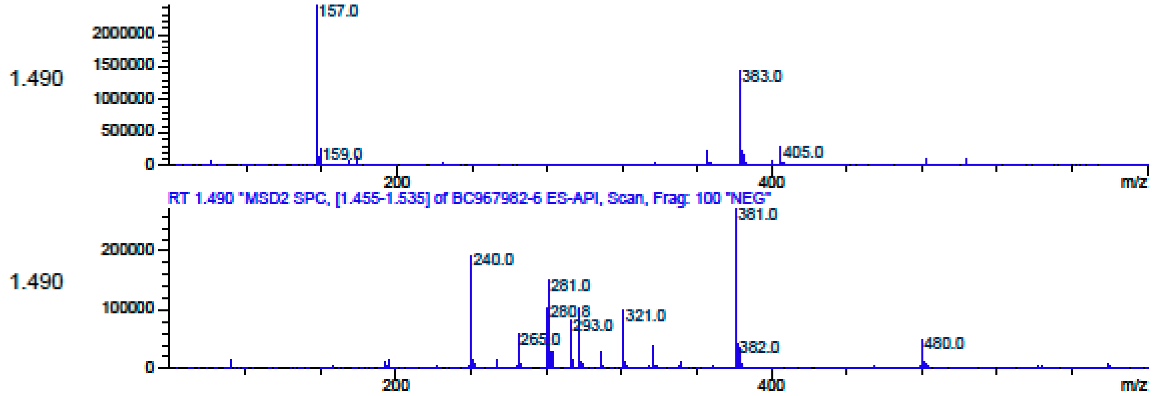

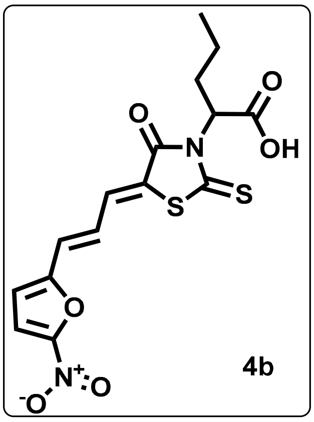

Molecular Weight: 382,41

Figure S18. LC-MS spectrum of compound **4b**

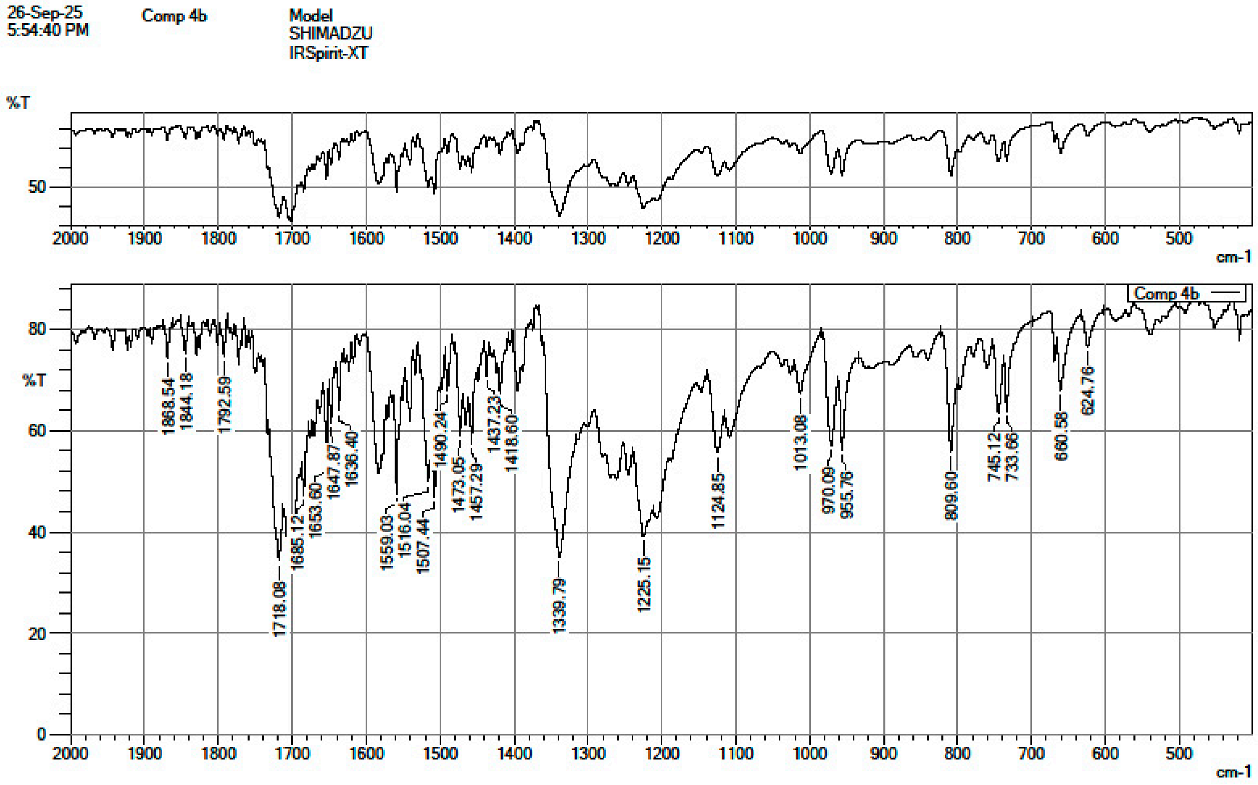

Figure S19. IR spectrum of compound **4b**

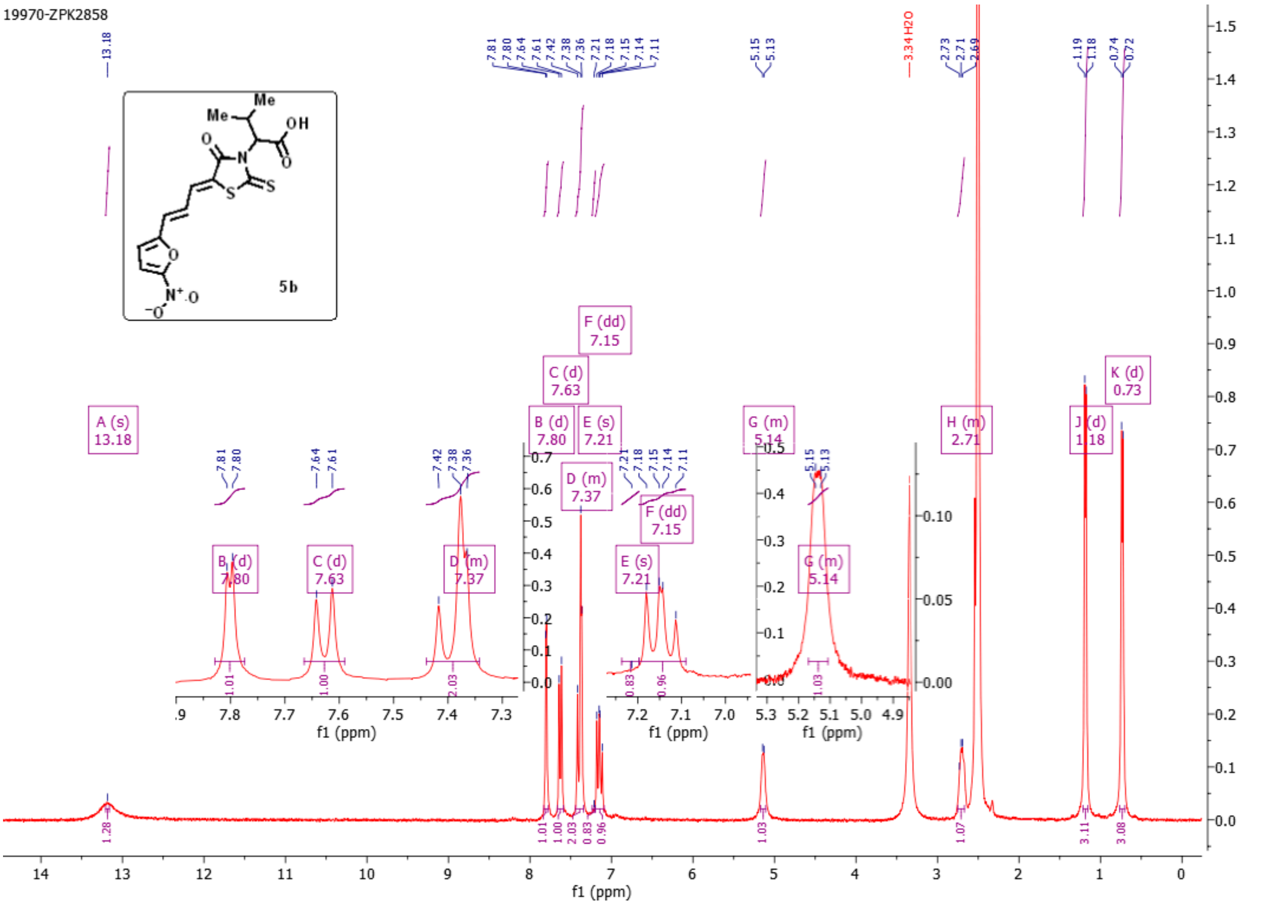

Figure S20. ^{1}H NMR spectrum of compound **5b**.

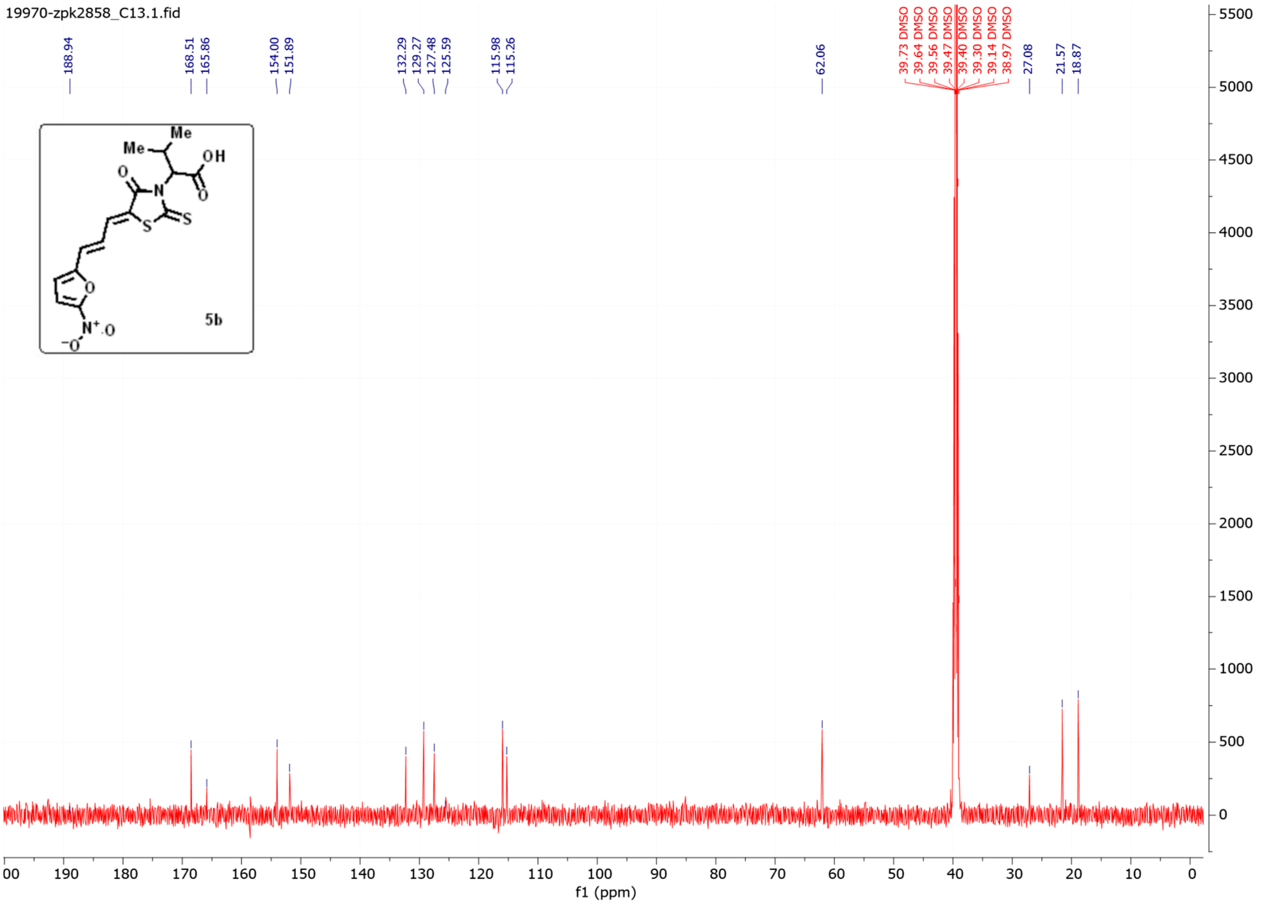

Figure S21. ^{13}C NMR spectrum of compound **5b**

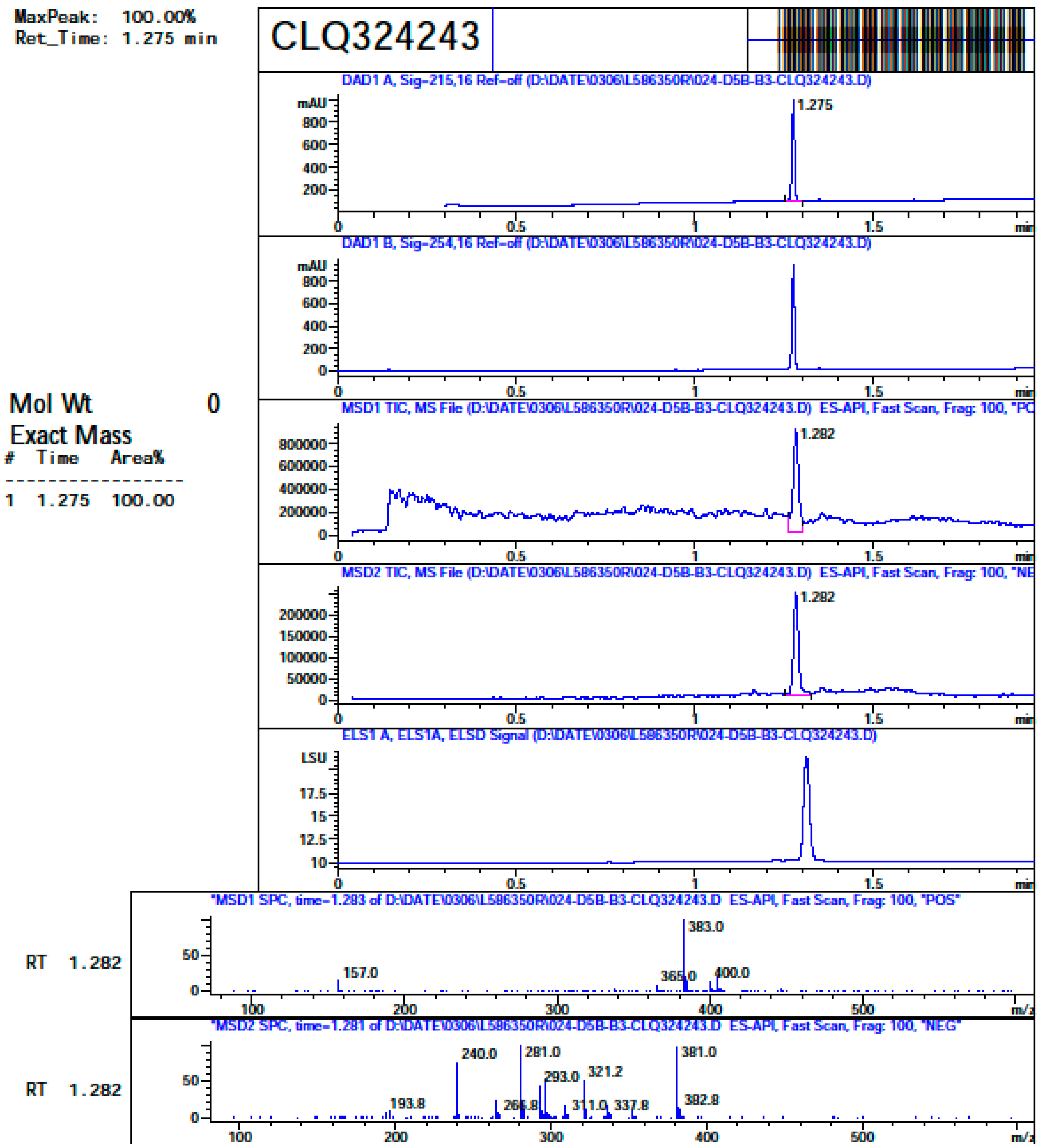

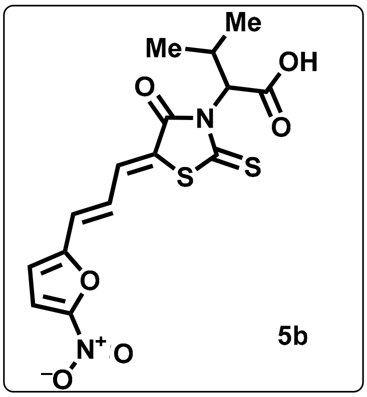

Molecular Weight: 382,41

Figure S22. LC-MS spectrum of compound **5b**

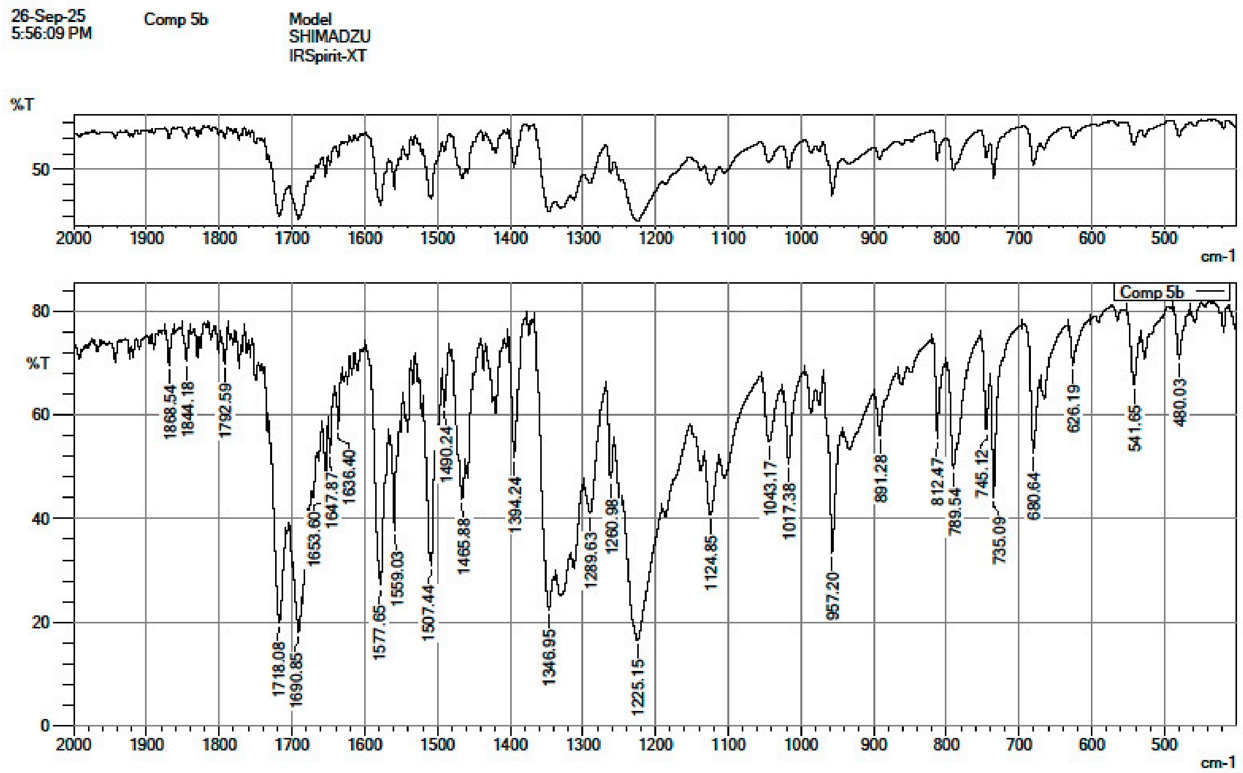

Figure S23. IR spectrum of compound **5b**

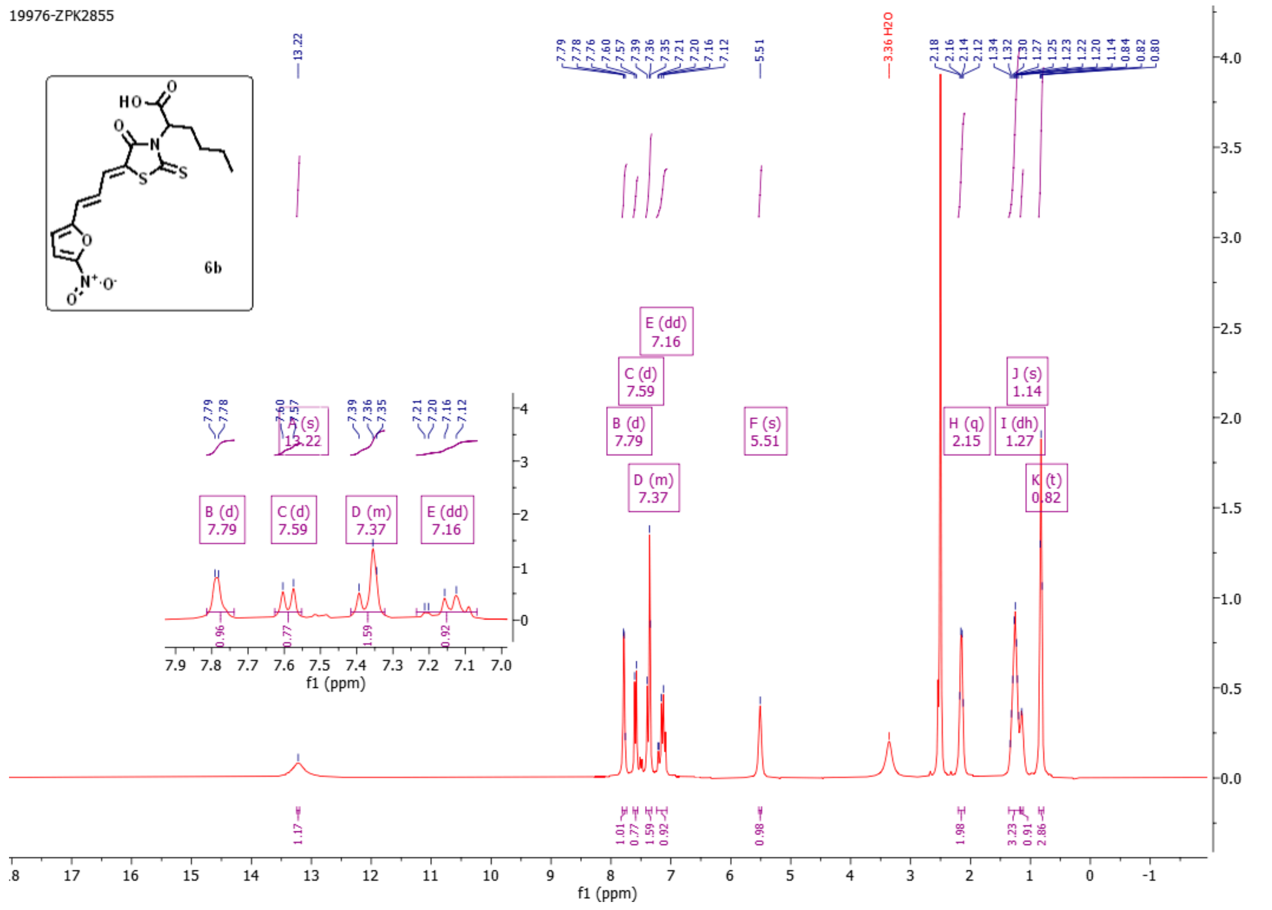

Figure S24. ^{1}H NMR spectrum of compound **6b**.

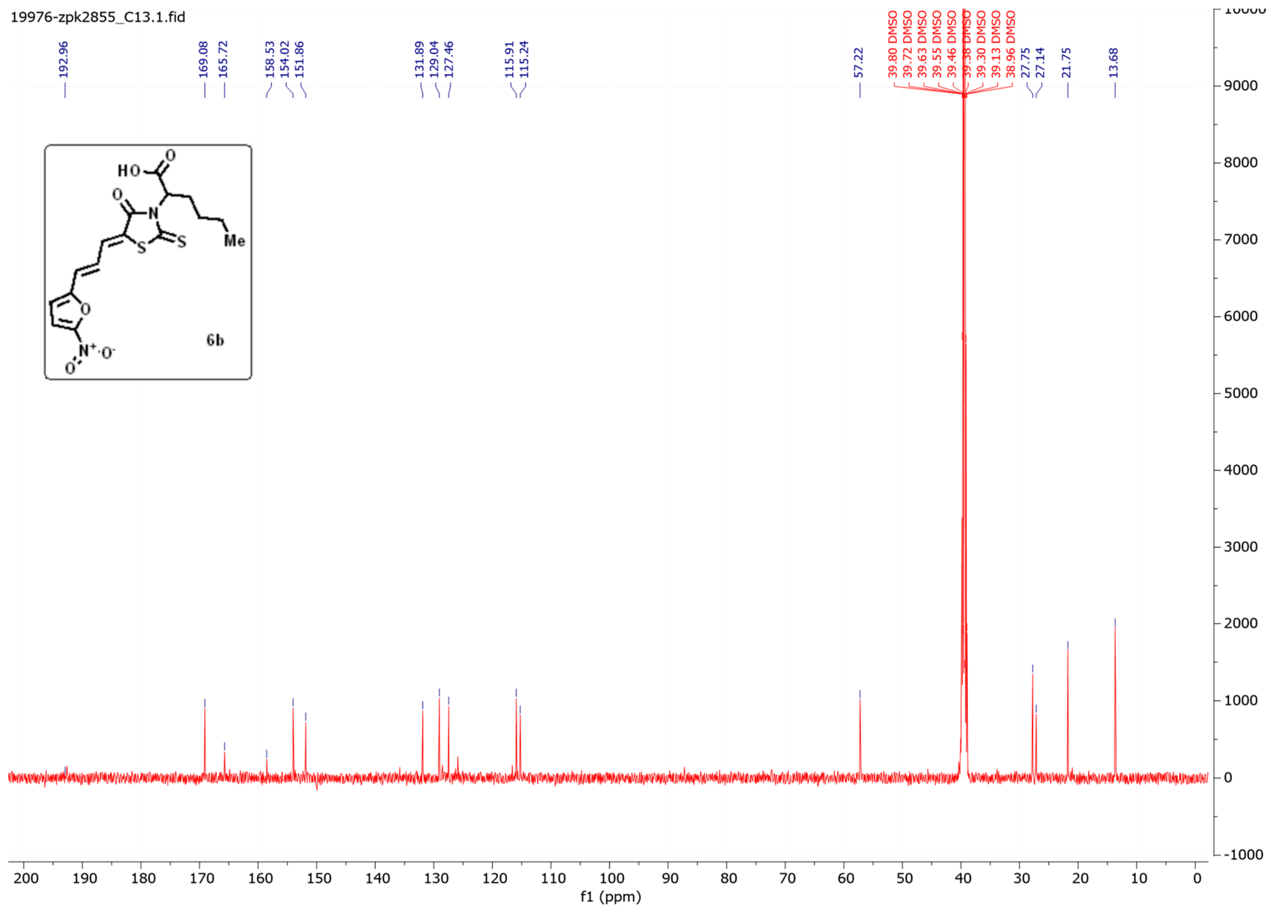

Figure S25. ^{13}C NMR spectrum of compound **6b**

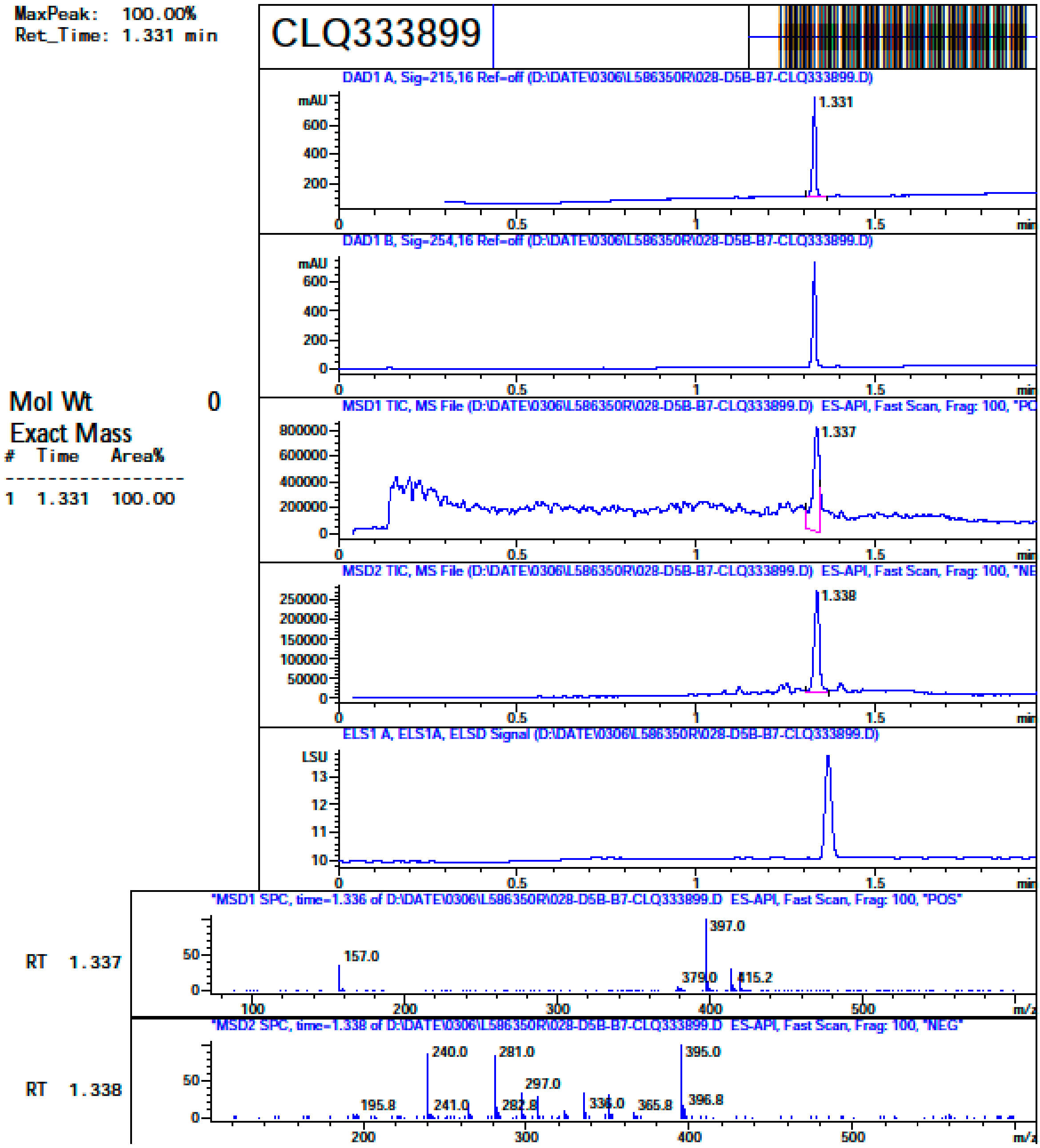

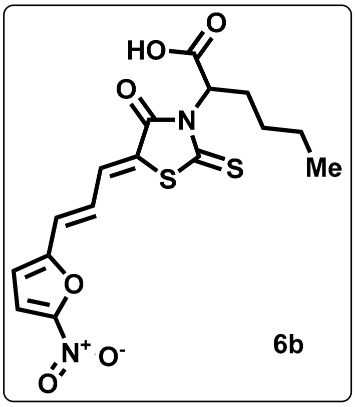

6b

Molecular Weight: 396,43

Figure S26. LC-MS spectrum of compound **6b**.

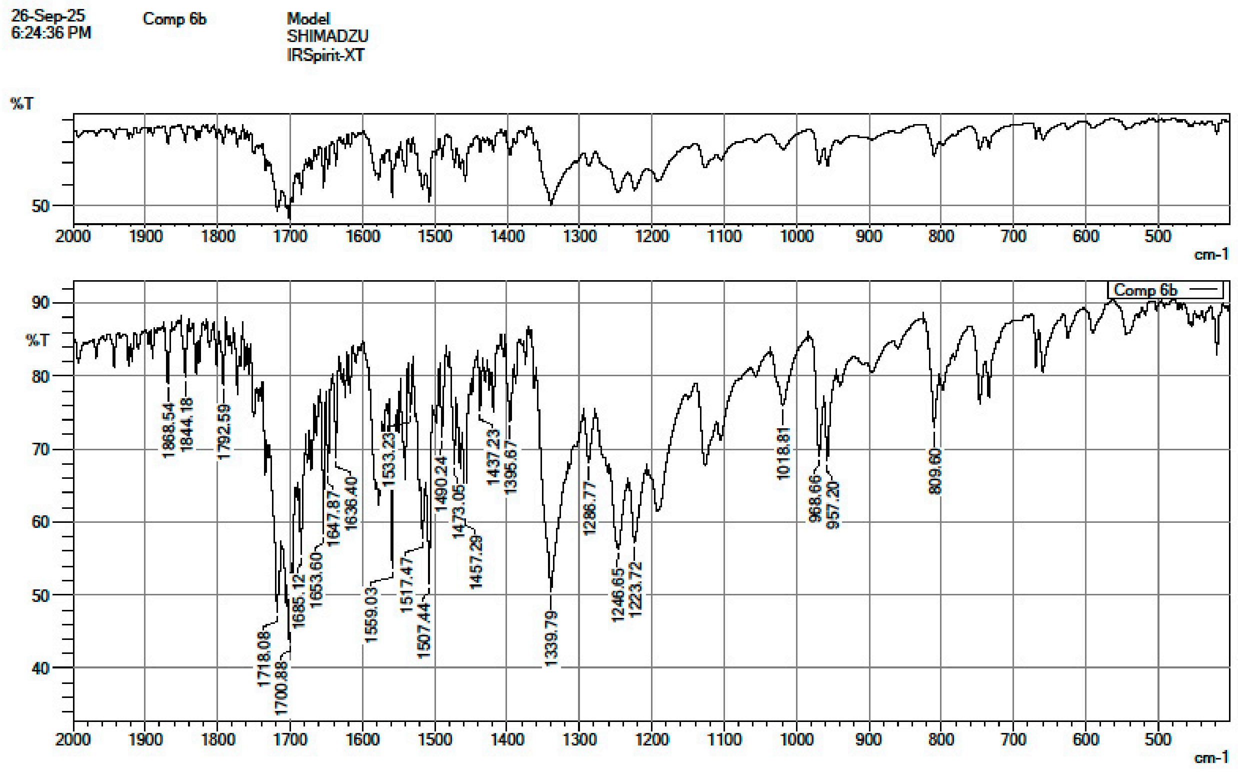

Figure S27. IR spectrum of compound **6b**

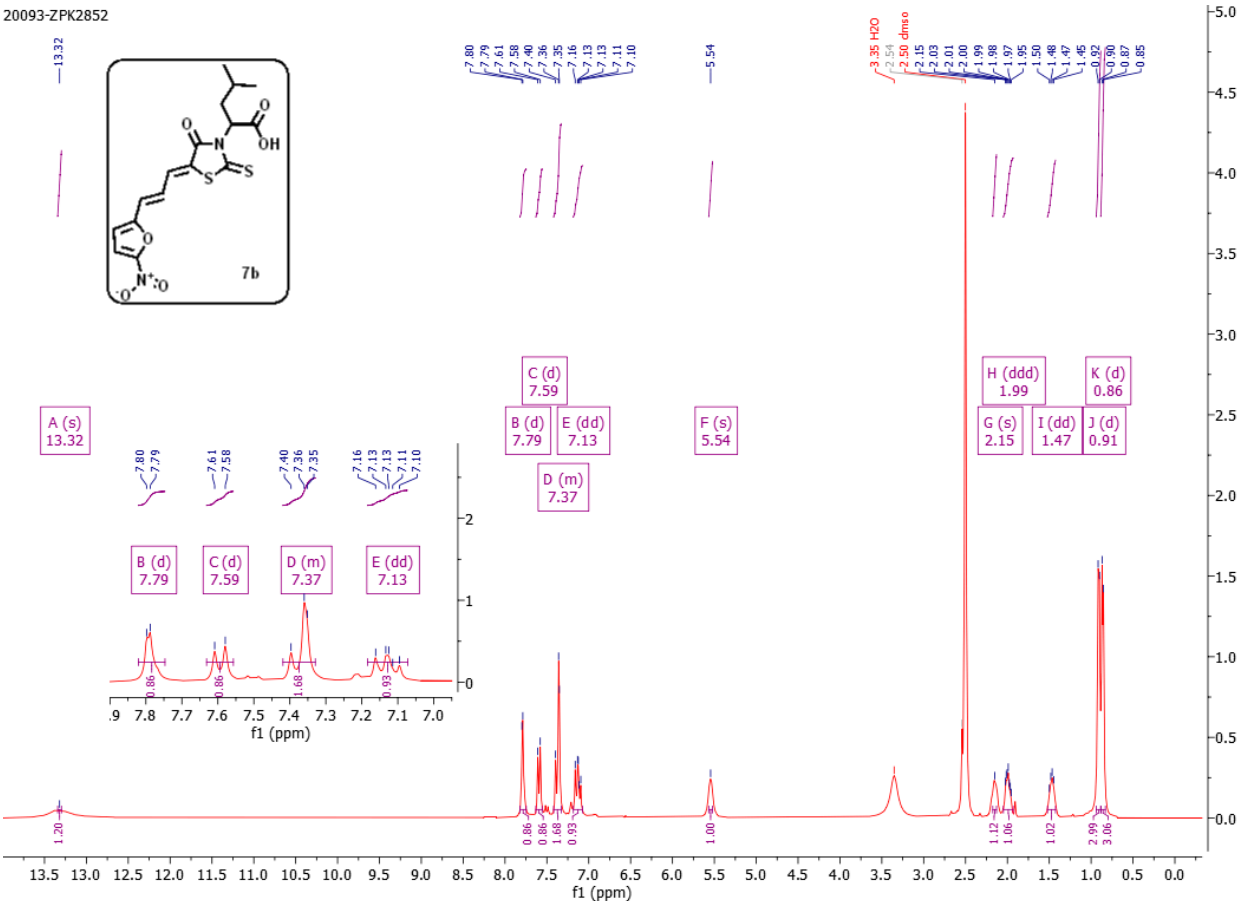

Figure S28. ^{1}H NMR spectrum of compound **7b**.

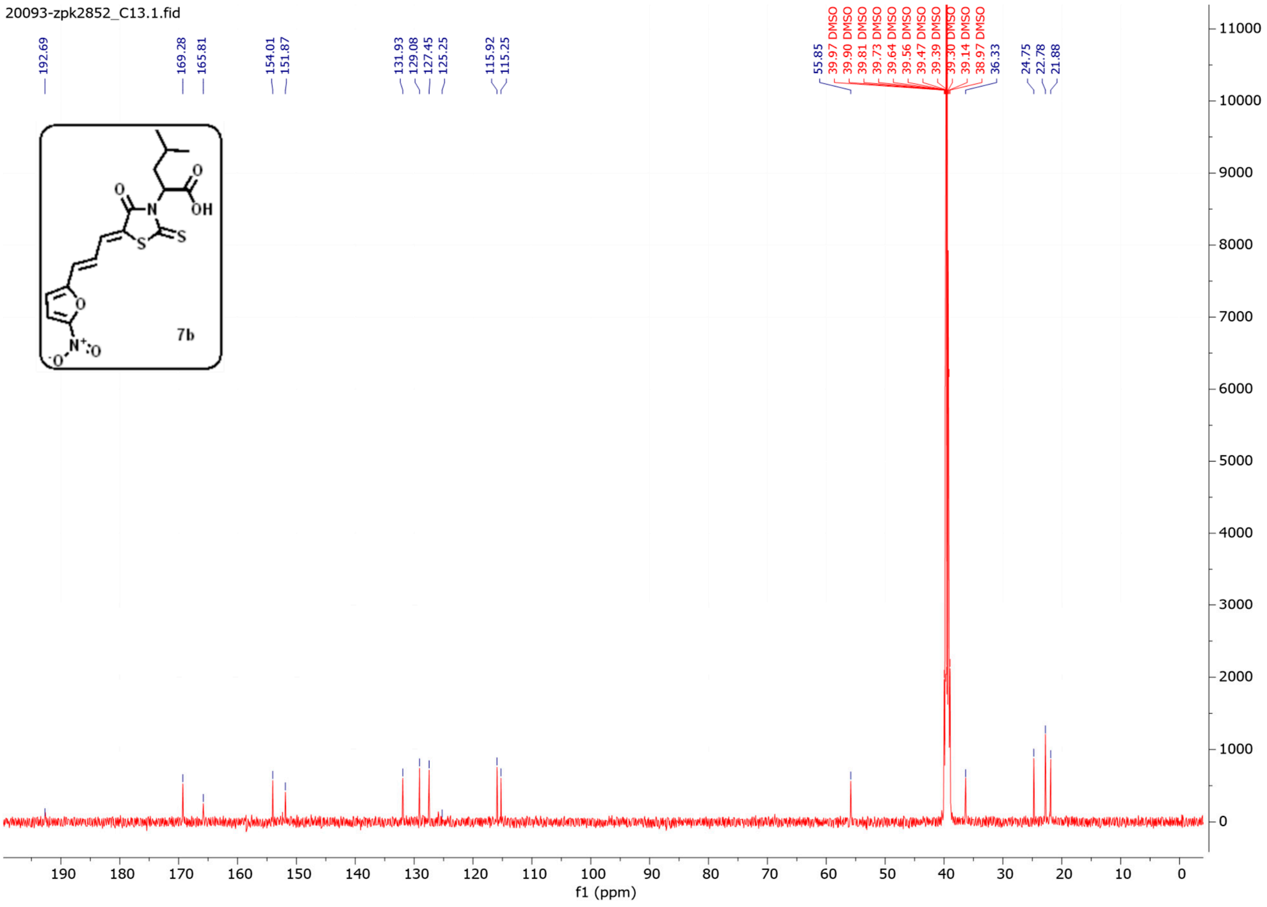

Figure S29. ^{13}C NMR spectrum of compound **7b**

#	RT	DAD1A	DAD1B	MSD1	MSD2	ELSD	MSD1 ions	MSD1 rt	MSD2 ions	MSD2 rt	Info
1	1.533	98.7%	98.5%	95.1%	92.9%	100.0%	397.0(62),419.0(18),414.0(12)	1.540	281.0(25),240.0(20),395.0(14)	1.540	
2	1.600	1.3%	1.5%	4.9%	7.1%	—	423.0(82),445.0(18)	1.607	266.0(32),307.0(31),421.0(26)	1.607	

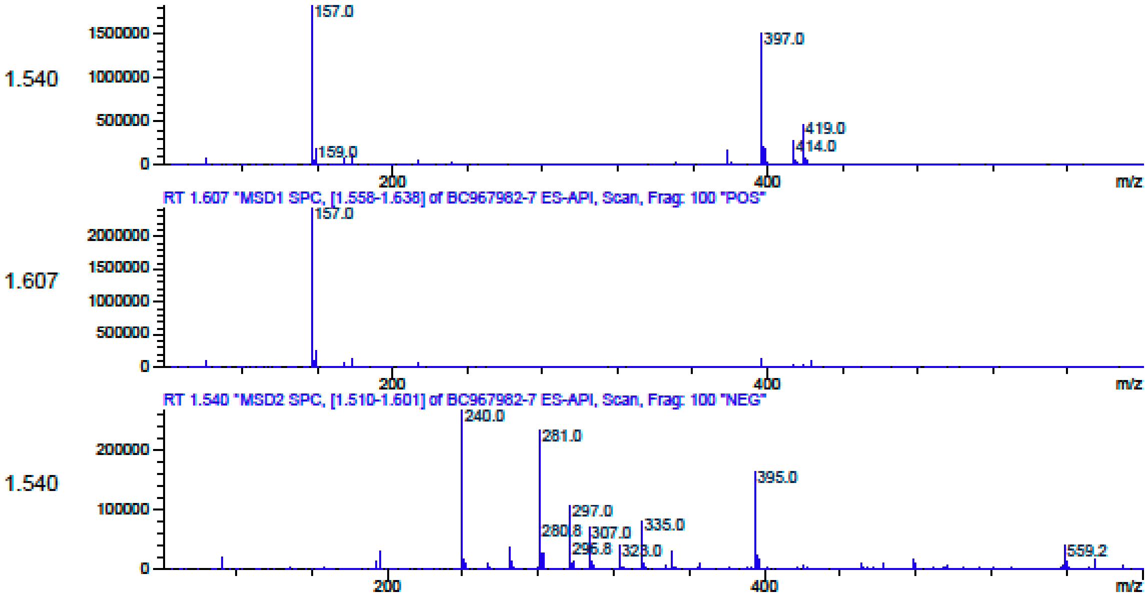

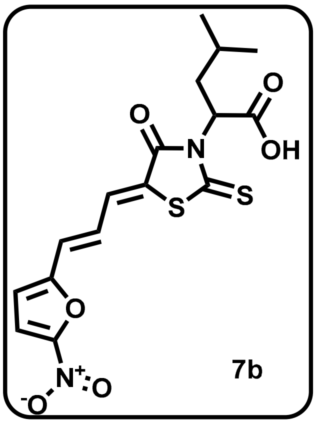

Molecular Weight: 396,43

Figure S30. LC-MS spectrum of compound **7b**

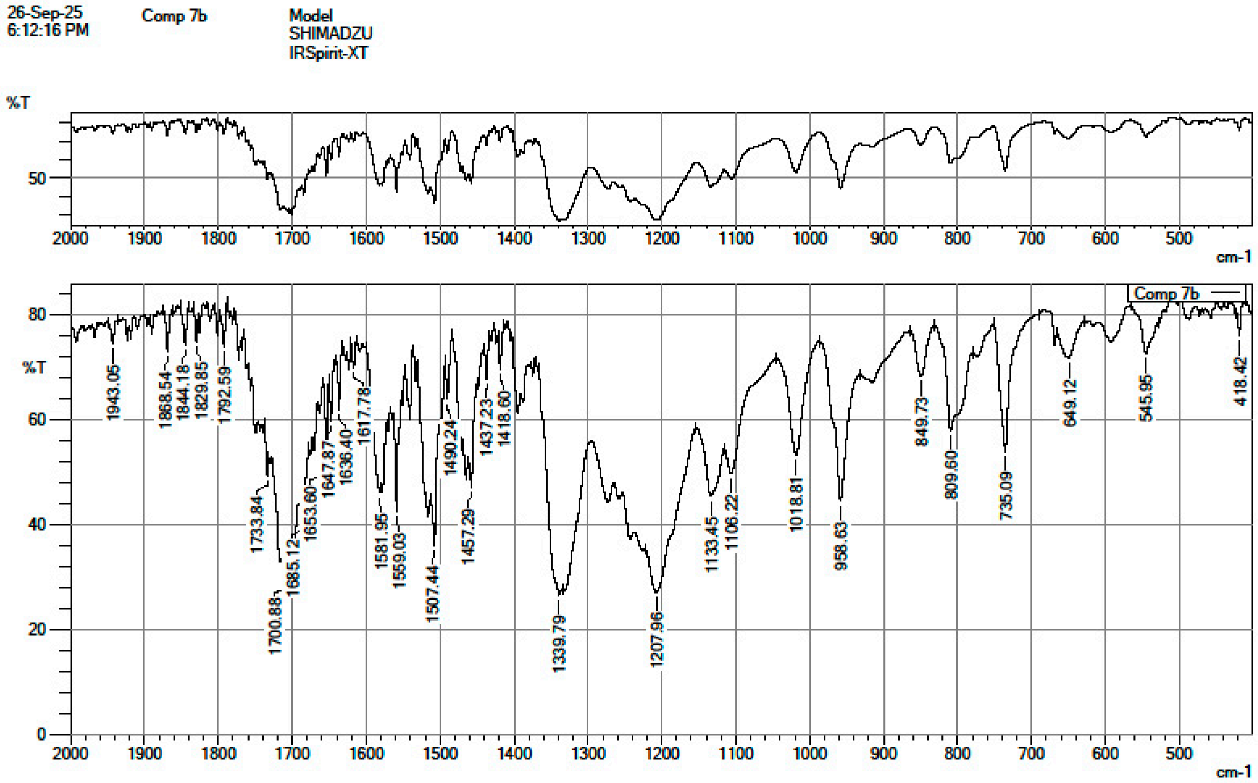

Figure S31. IR spectrum of compound **7b**

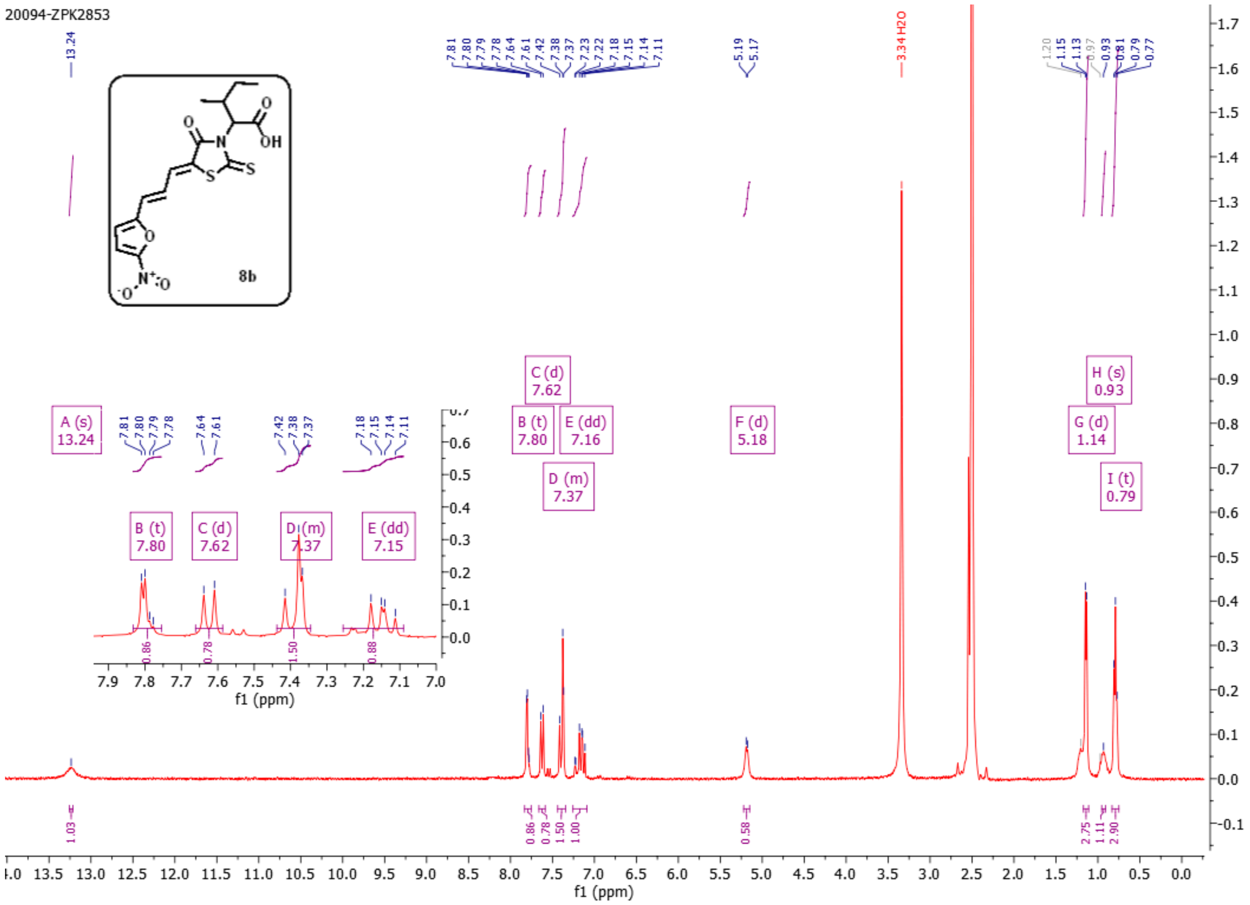

Figure S32. ^{1}H NMR spectrum of compound **8b**.

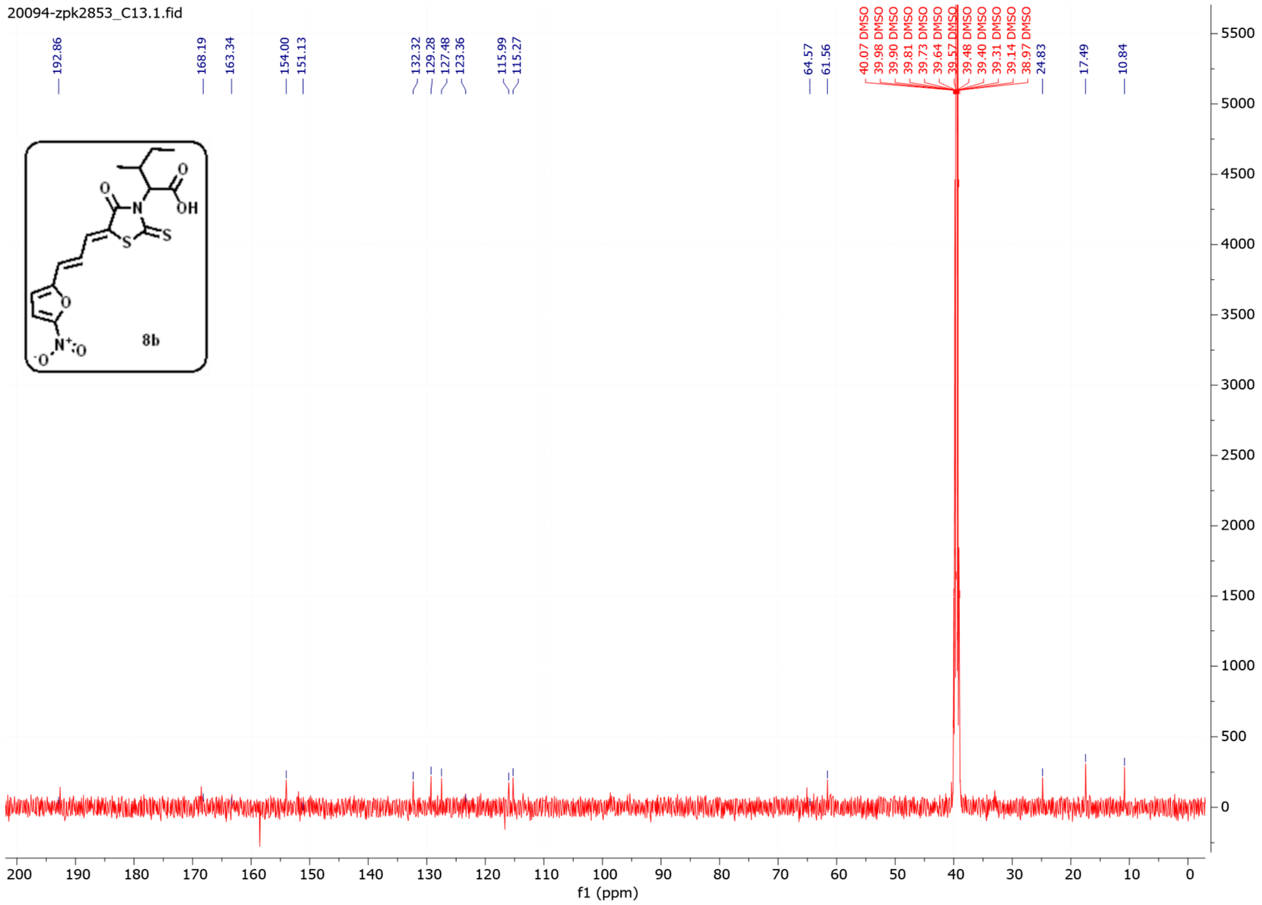

Figure S33. ^{13}C NMR spectrum of compound **8b**

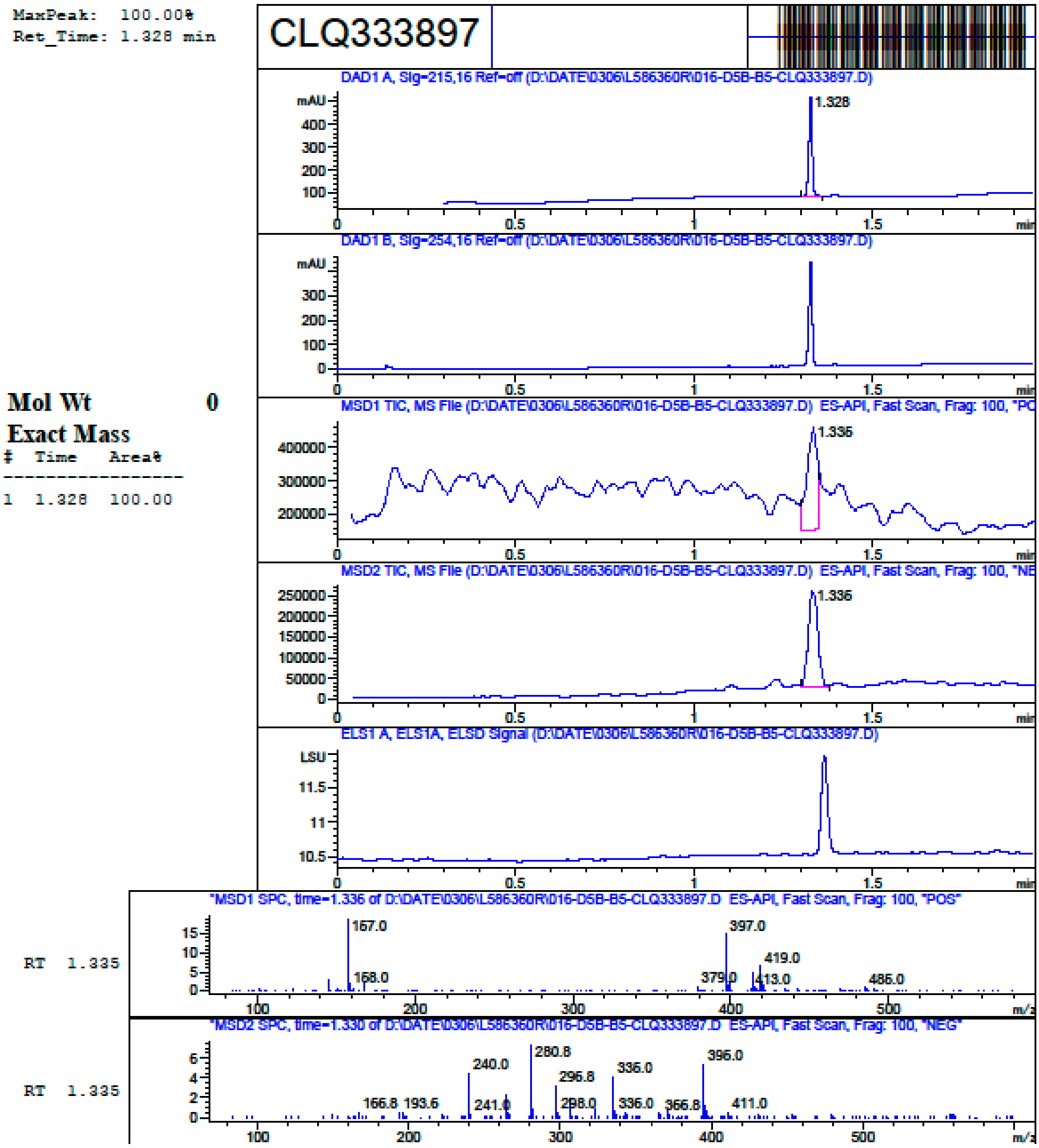

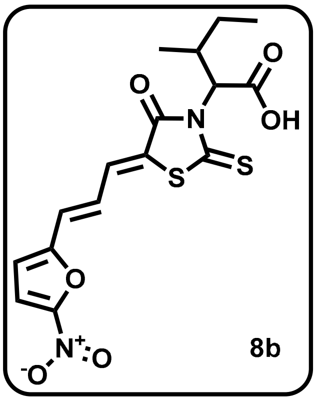

Molecular Weight: 396,43

Figure S34. LC-MS spectrum of compound **8b**

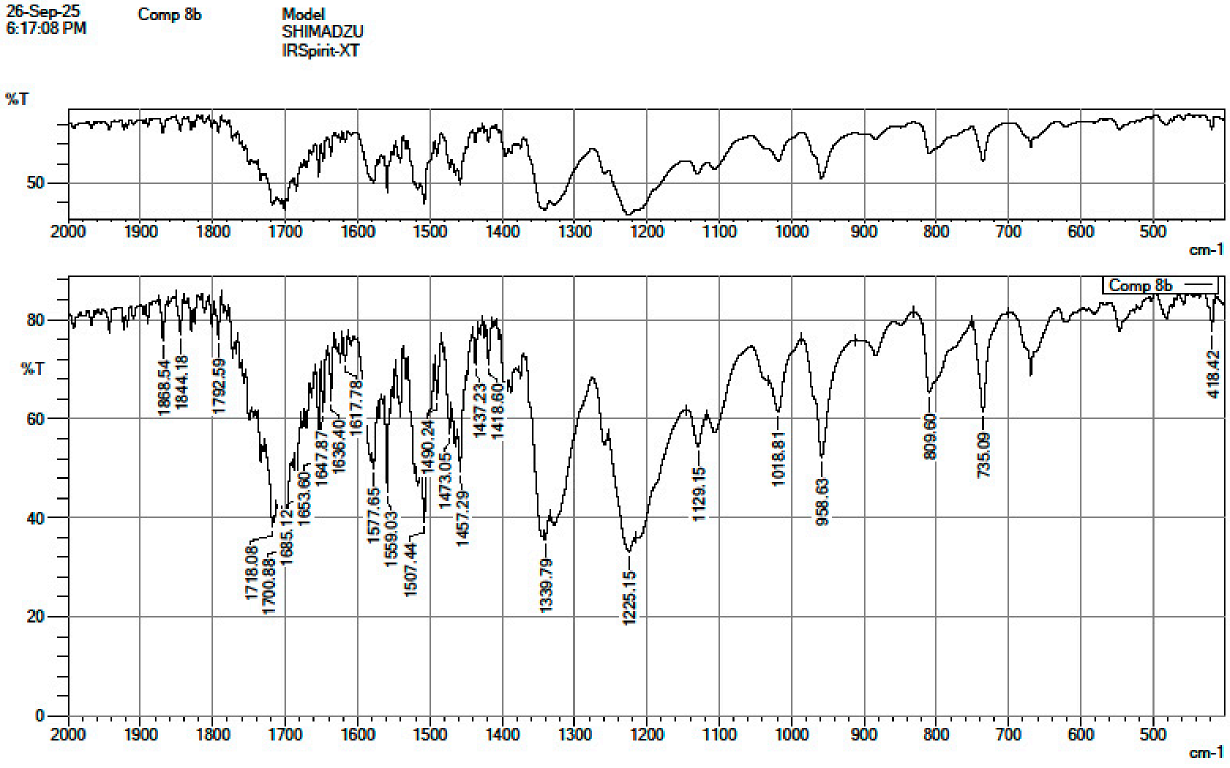

Figure S35. IR spectrum of compound **8b**

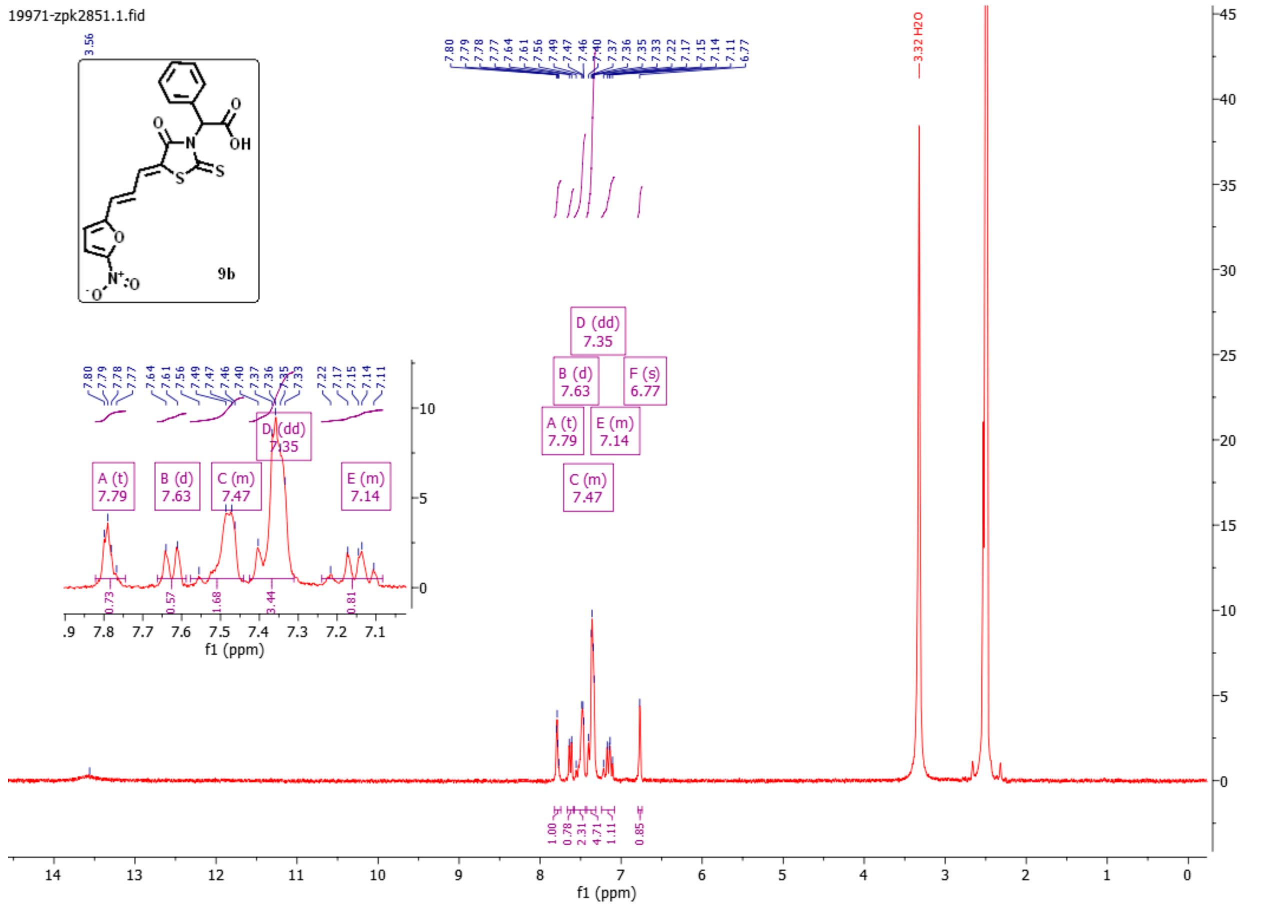

Figure S36. ^{1}H NMR spectrum of compound **9b**.

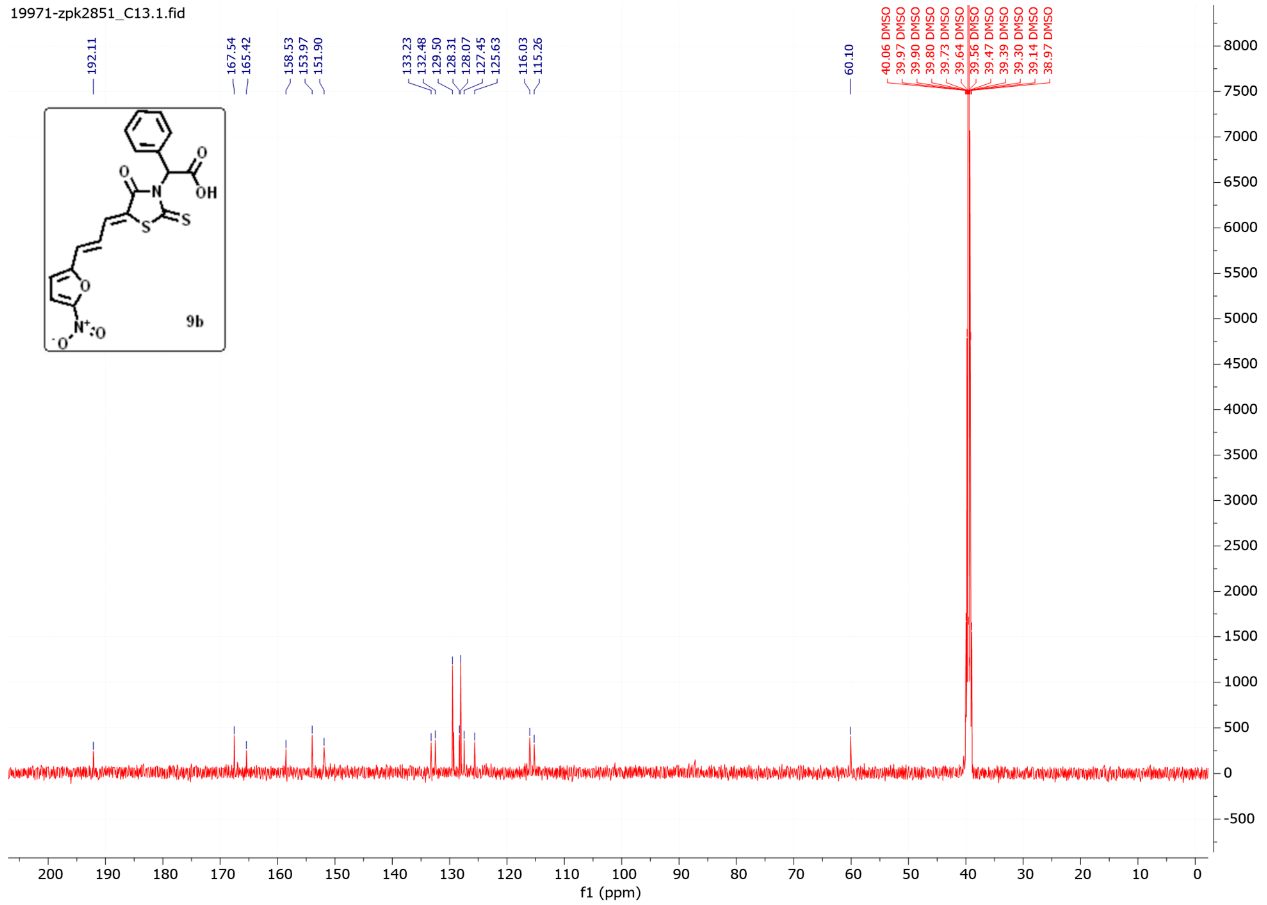

Figure S37. ^{13}C NMR spectrum of compound **9b**

#	RT	DAD1A	DAD1B	MSD1	MSD2	ELSD	MSD1 ions	MSD1 rt	MSD2 ions	MSD2 rt	Info
1	1.460	—	—	35.8%	—	—	434.0(55),418.0(26),439.0(16)	1.466	—	—	
2	1.471	100.0%	100.0%	64.2%	100.0%	100.0%	417.0(80),419.0(12),399.0(8)	1.477	281.0(46),240.0(32),296.8(16)	1.479	

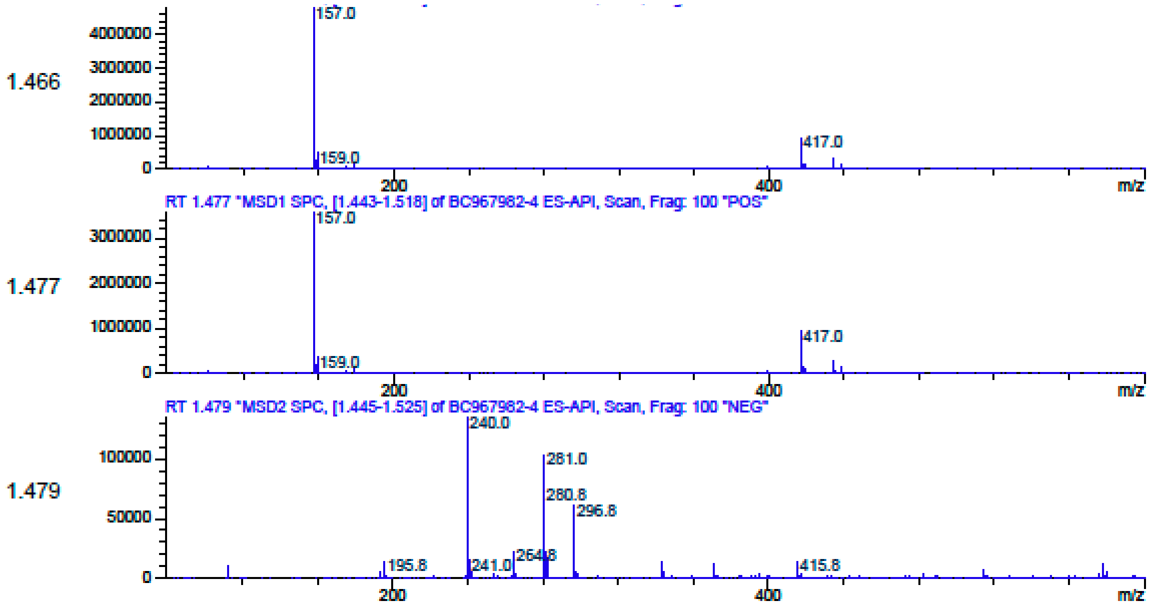

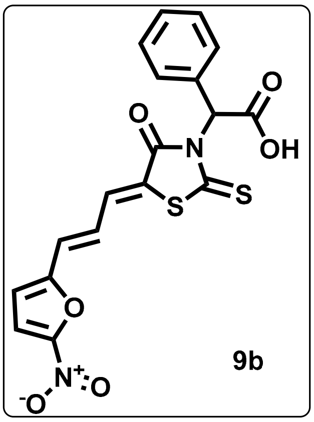

Molecular Weight: 416,42

Figure S38. LC-MS spectrum of compound **9b**

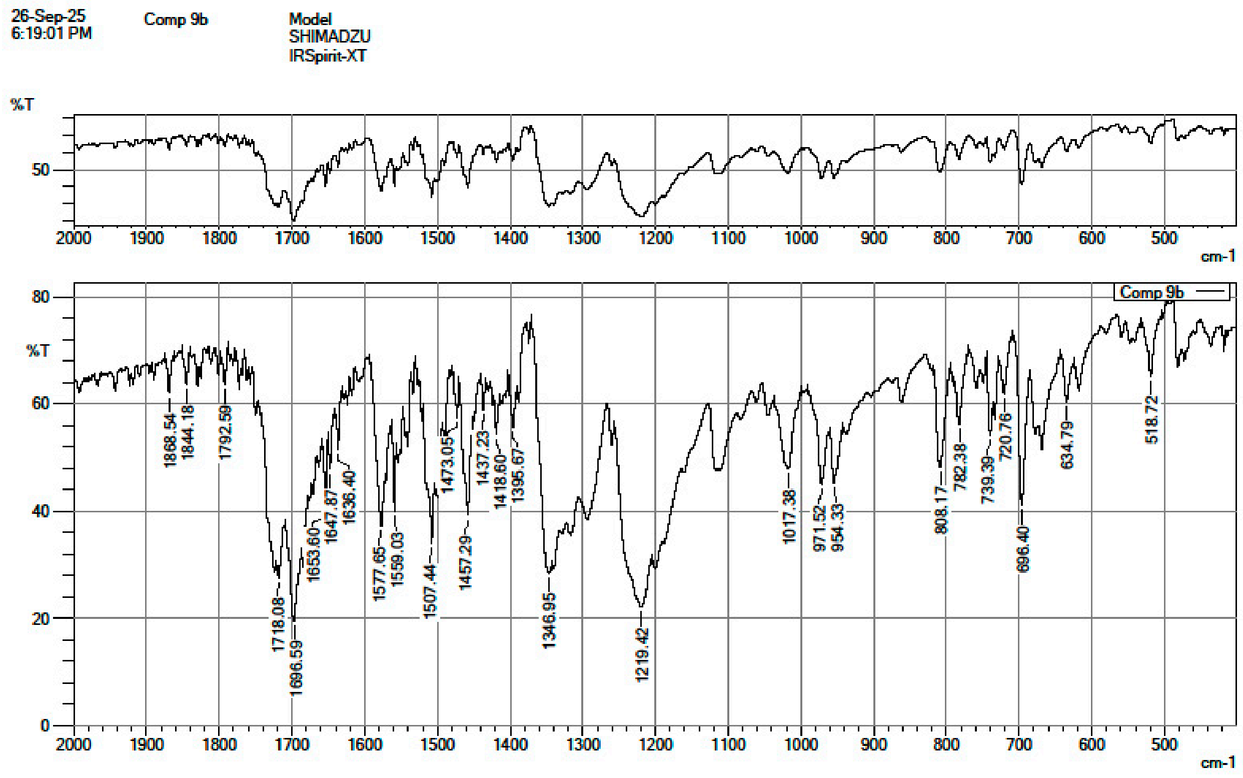

Figure S39. IR spectrum of compound **9b**

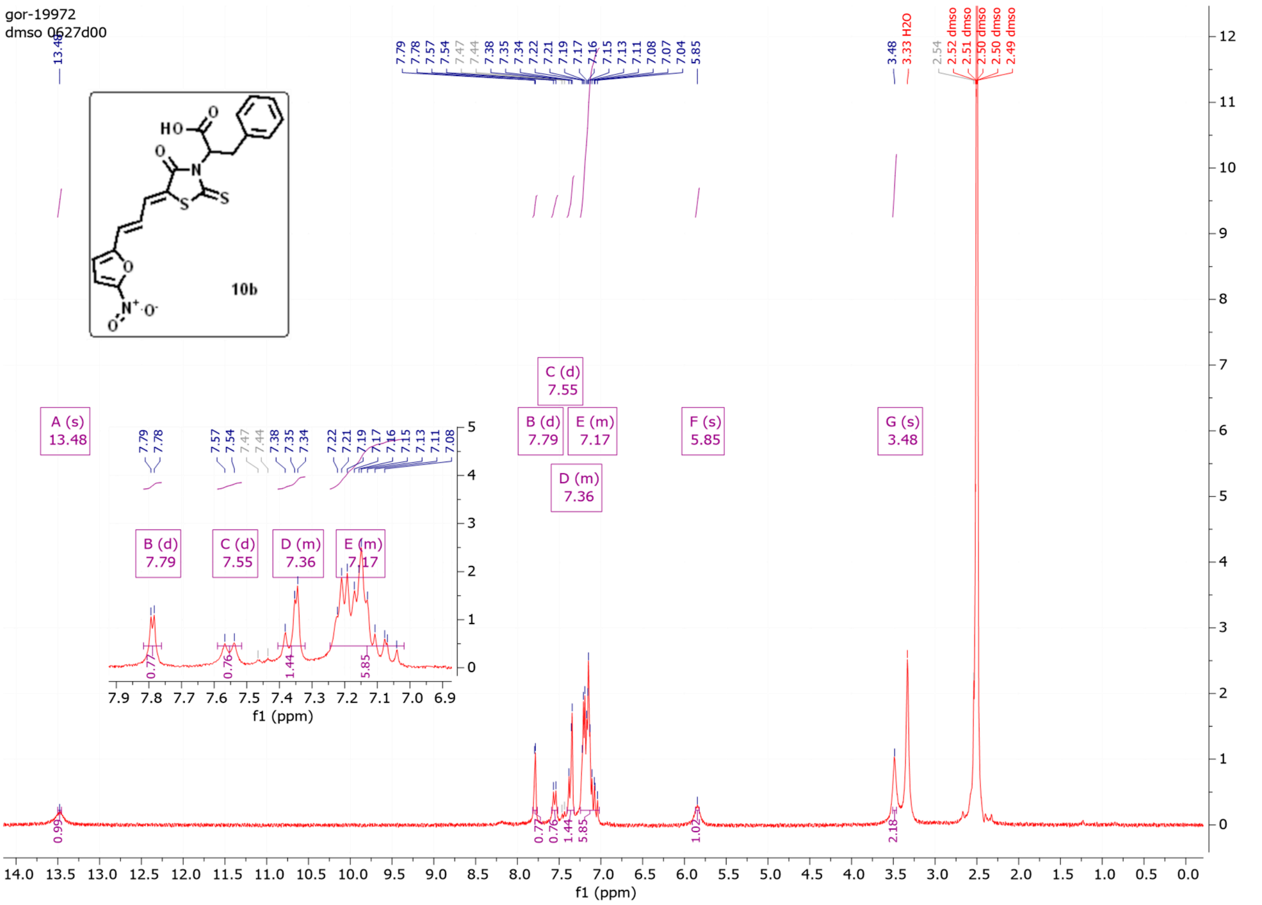

Figure S40. ^{1}H NMR spectrum of compound **10b**

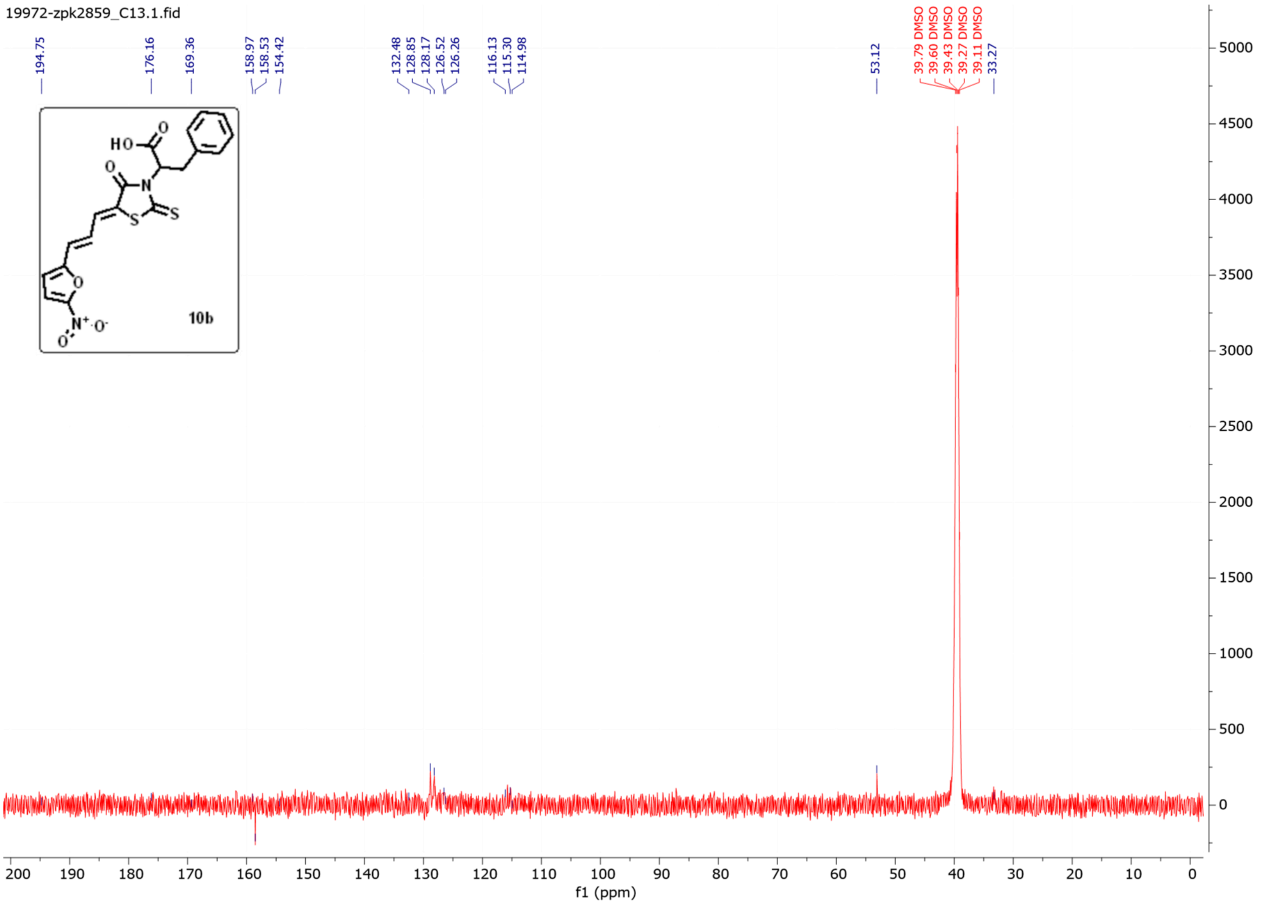

Figure S41. ^{13}C NMR spectrum of compound **10b**

#	RT	DAD1A	DAD1B	MSD1	MSD2	ELSD	MSD1 ions	MSD1 rt	MSD2 ions	MSD2 rt	Info
1	1.436	—	—	30.2%	13.3%	—	562.2(77),441.2(23)	1.442	516.2(73),439.2(27)	1.442	
2	1.479	100.0%	100.0%	69.8%	86.7%	100.0%	431.0(80),453.0(13),413.0(7)	1.487	280.8(28),239.8(24),296.8(21)	1.488	

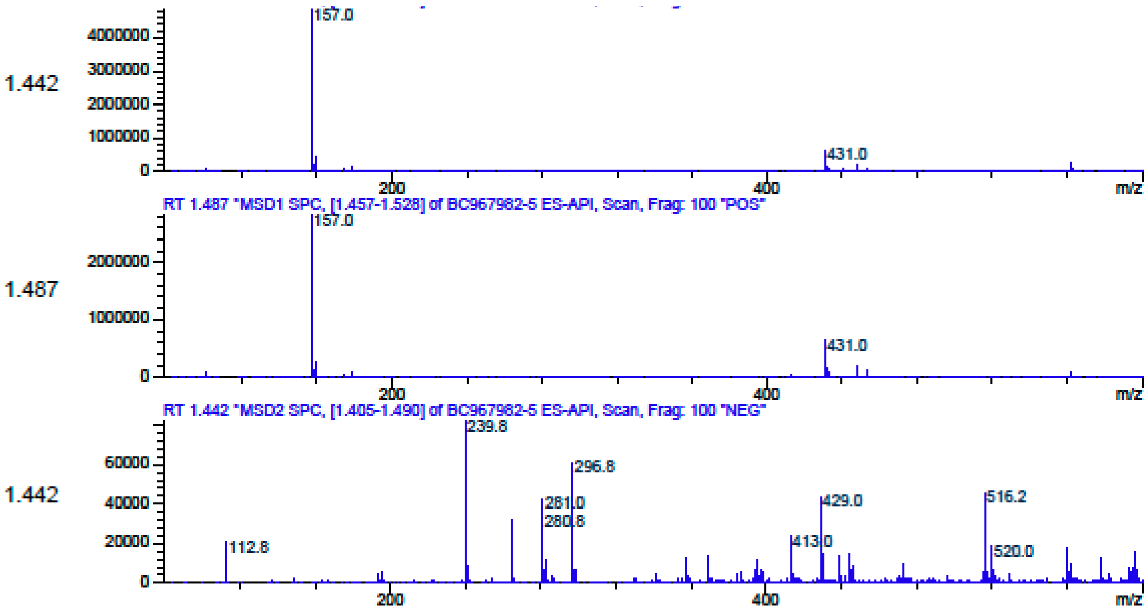

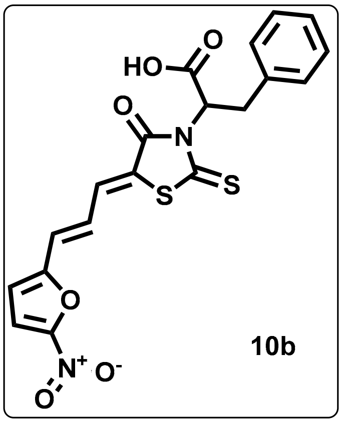

Molecular Weight: 430,45

Figure S42. LC-MS spectrum of compound **10b**

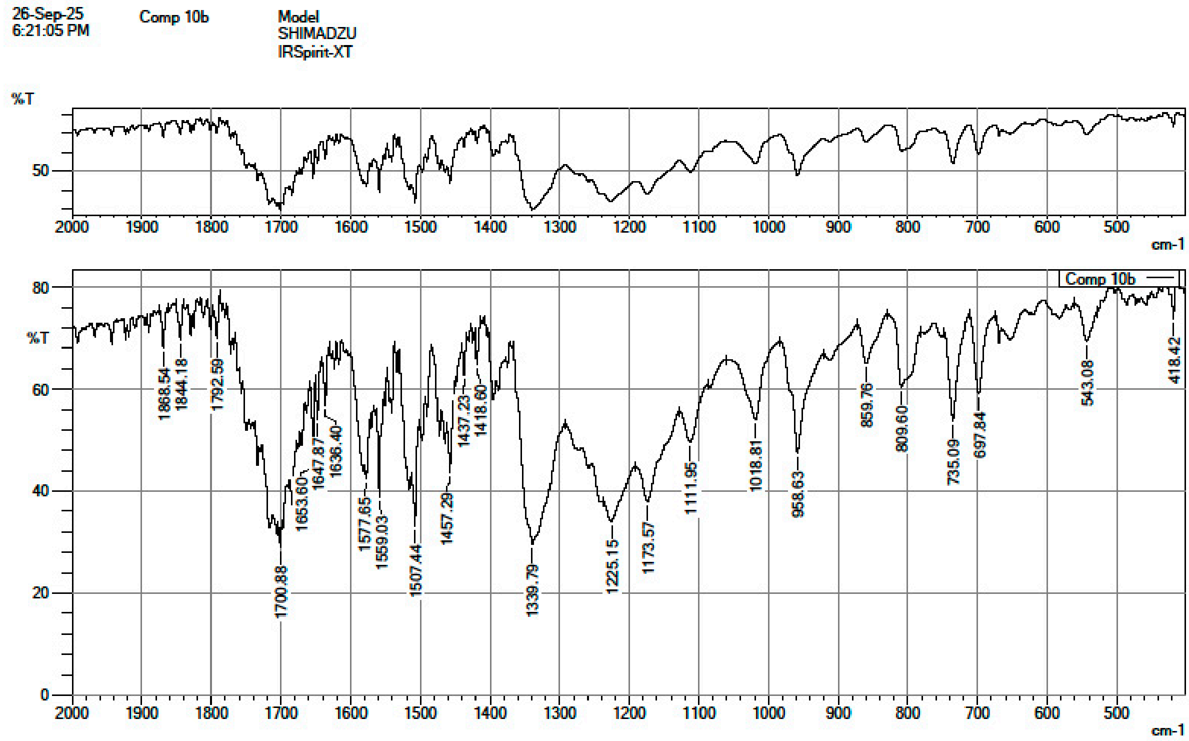

Figure S43. IR spectrum of compound **10b**

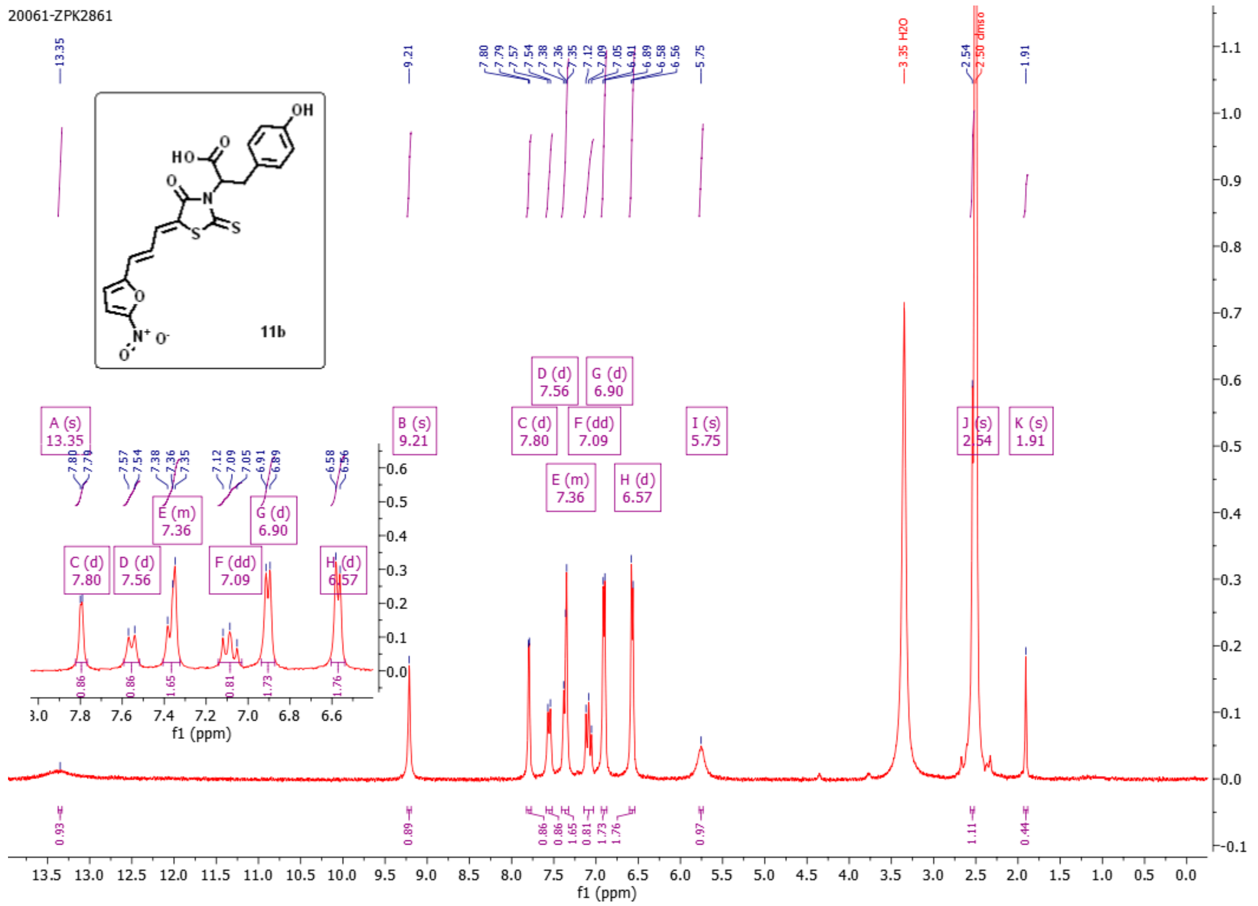

Figure S44. ^{1}H NMR spectrum of compound **11b**.

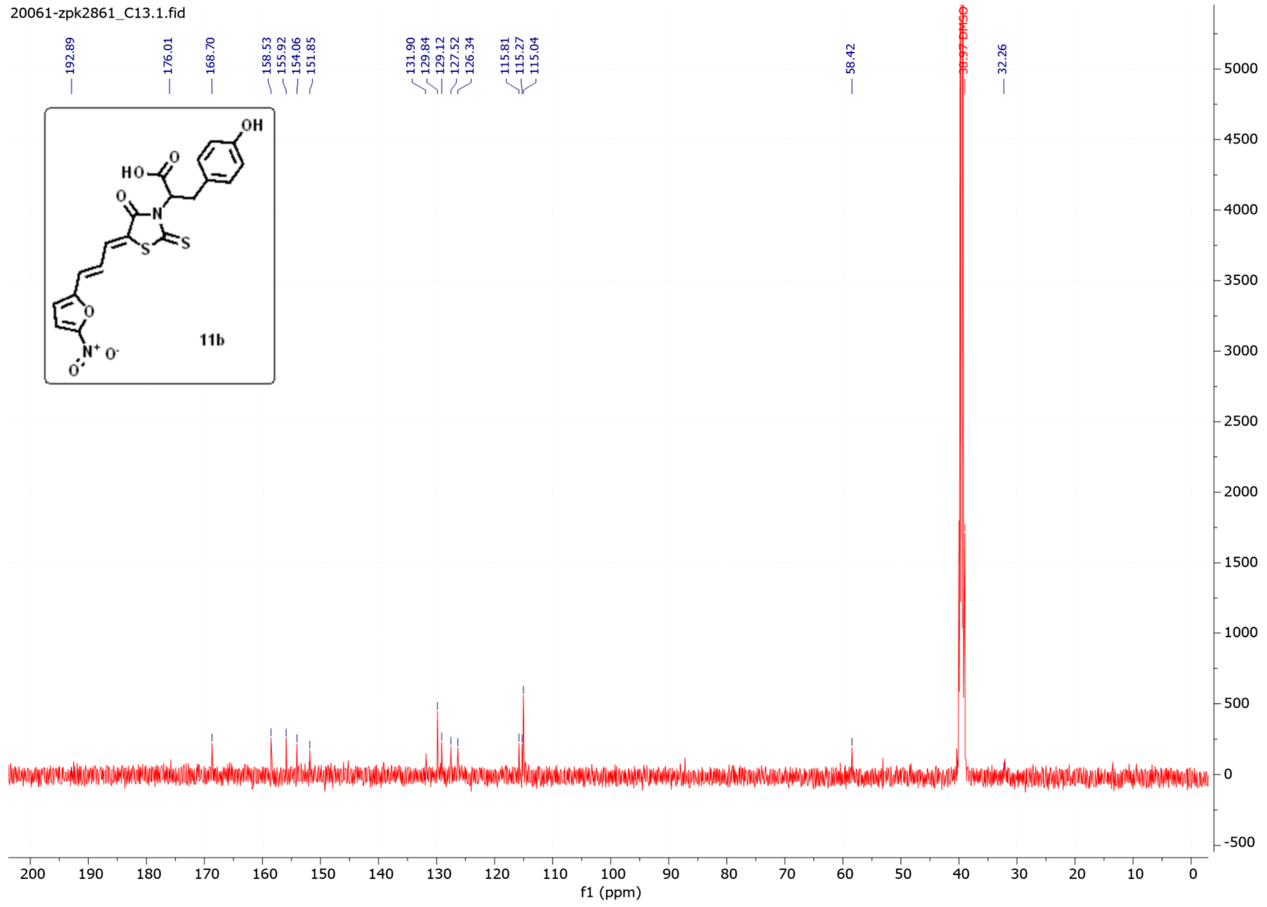

Figure S45. ^{13}C NMR spectrum of compound **11b**

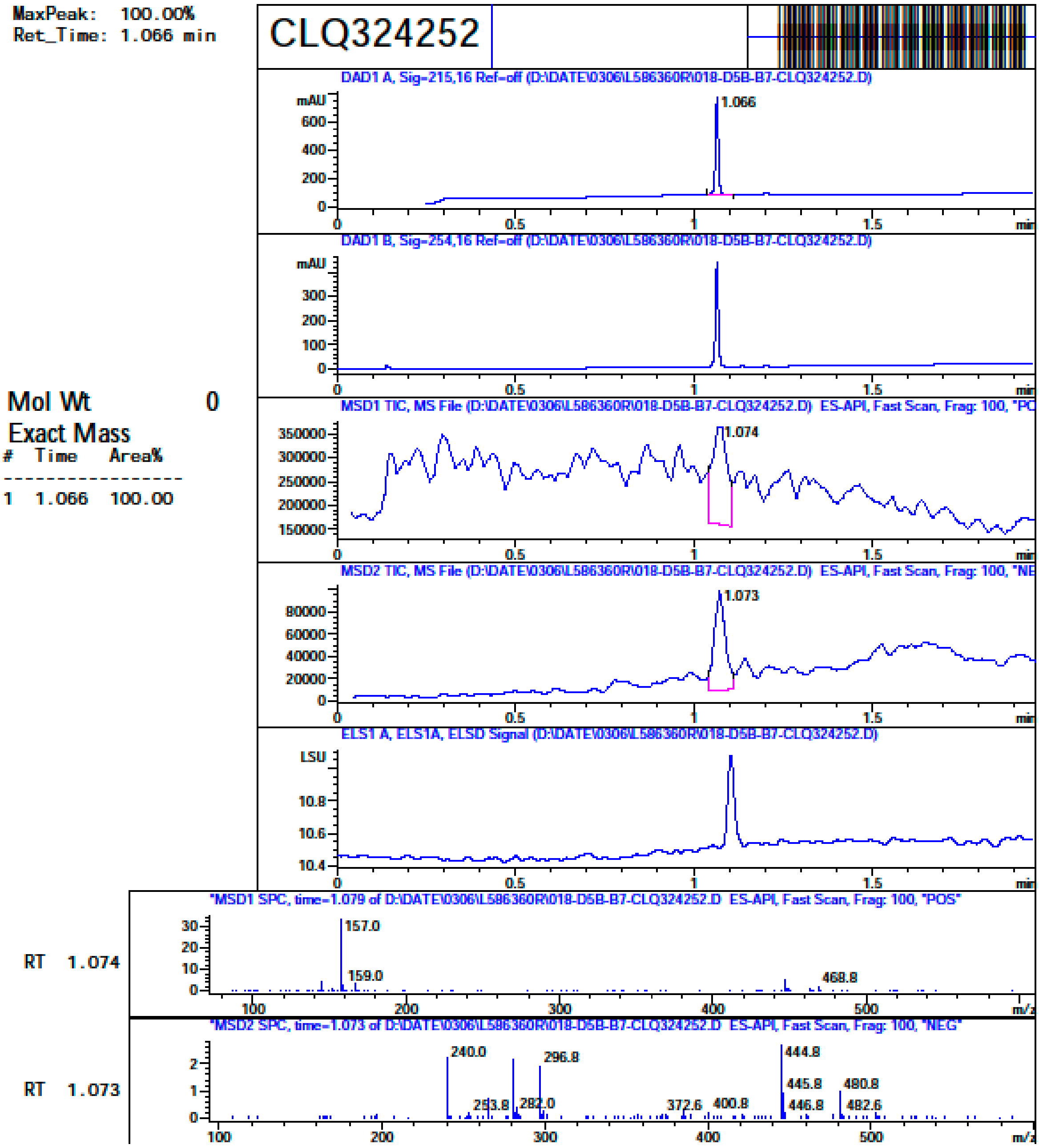

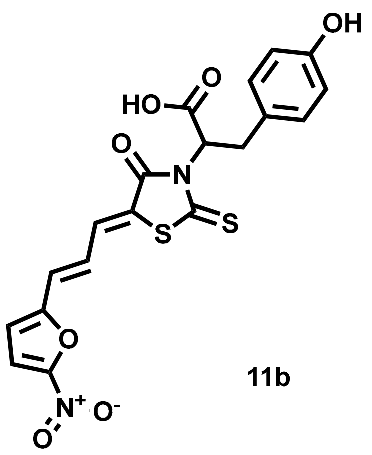

11b

Molecular Weight: 446,45

Figure S46. LC-MS spectrum of compound **11b**

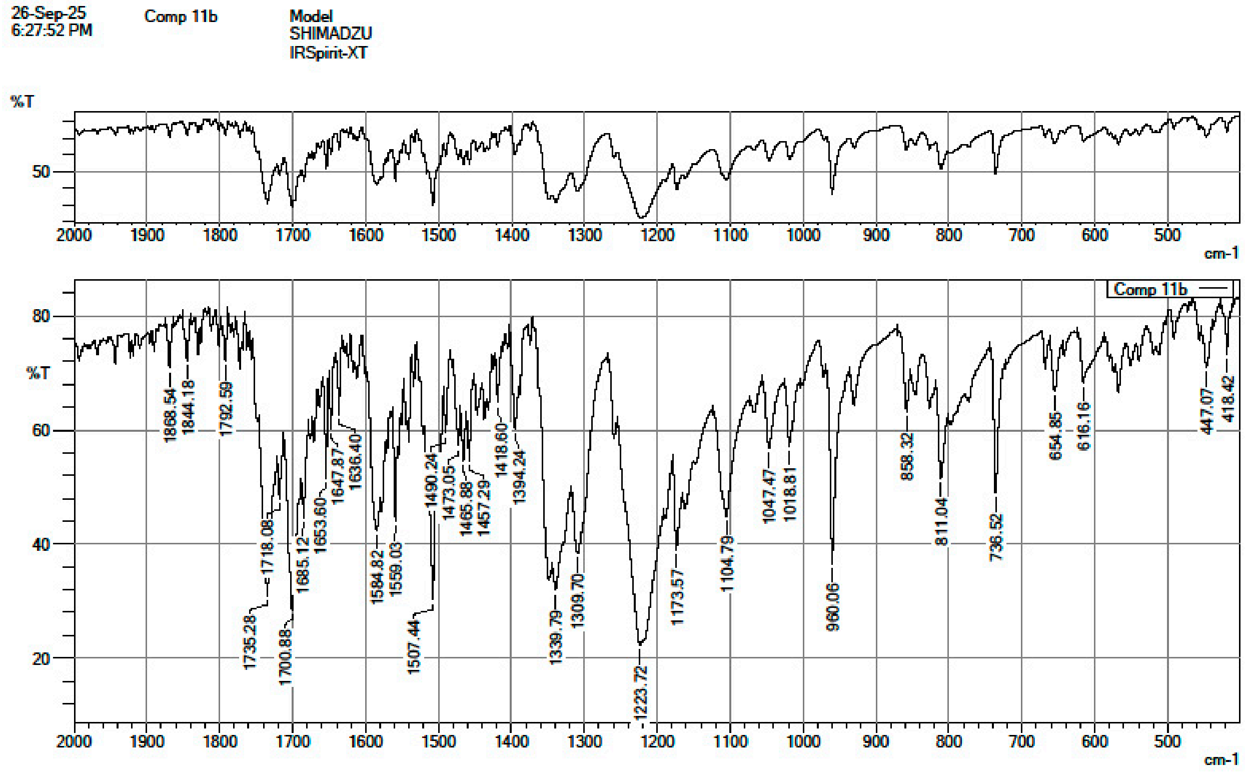

Figure S47. IR spectrum of compound **11b**

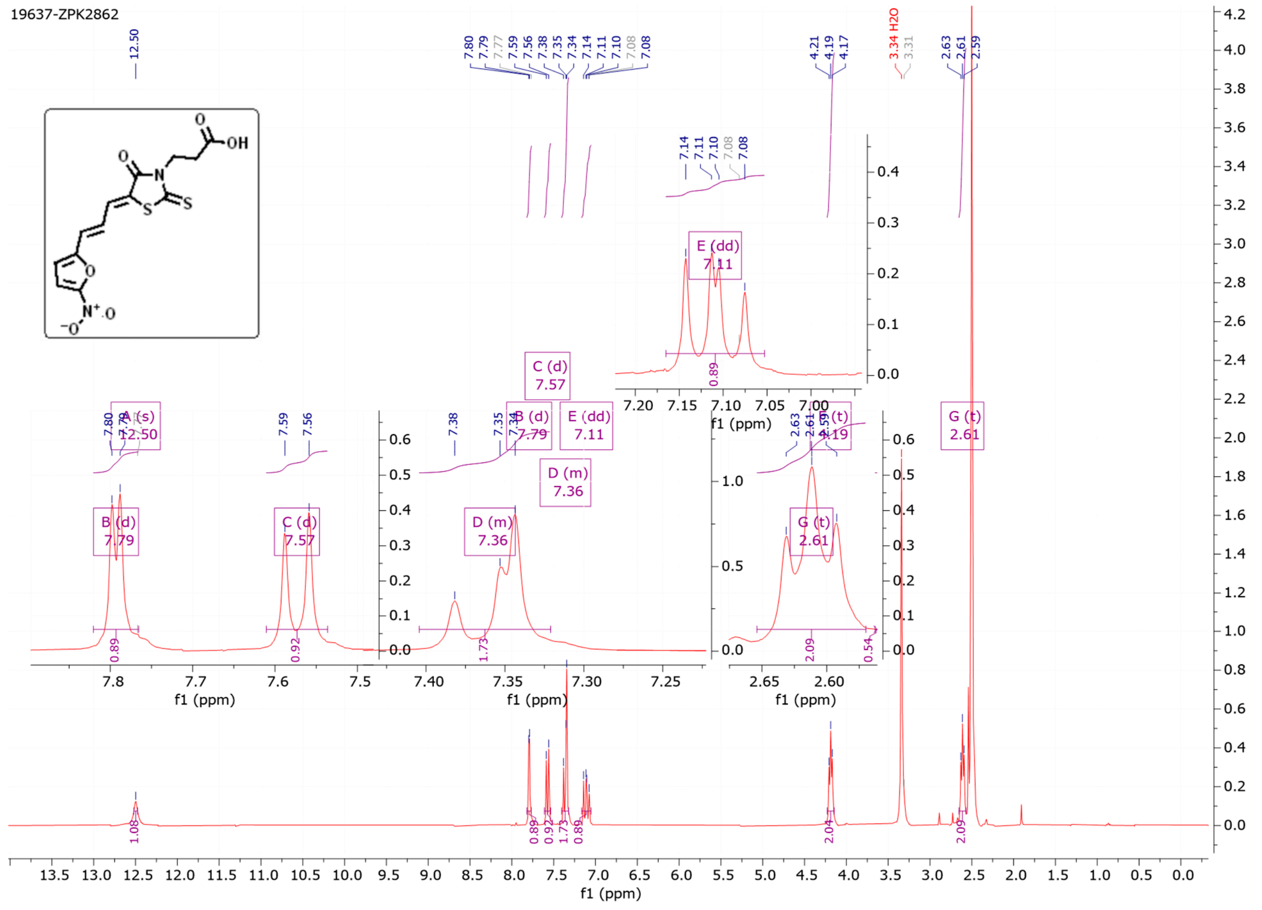

Figure S48. ^{1}H NMR spectrum of compound **12b**

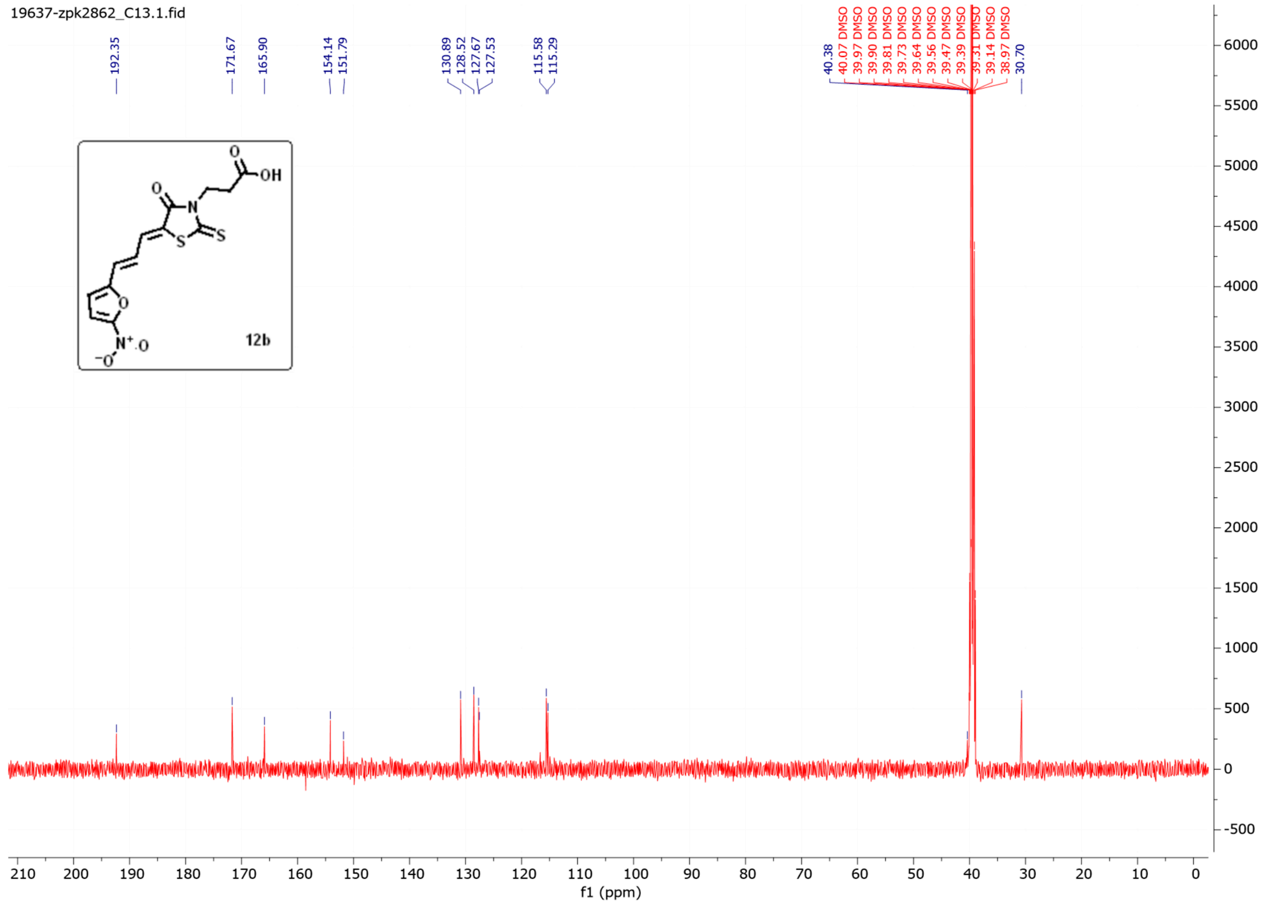

Figure S49. ^{13}C NMR spectrum of compound **12b**

MaxPeak: 100.00%
Ret_Time: 1.096 min

Mol Wt 0
Exact Mass

#	Time	Area%
1	1.096	100.00

RT 1.103

RT 1.103

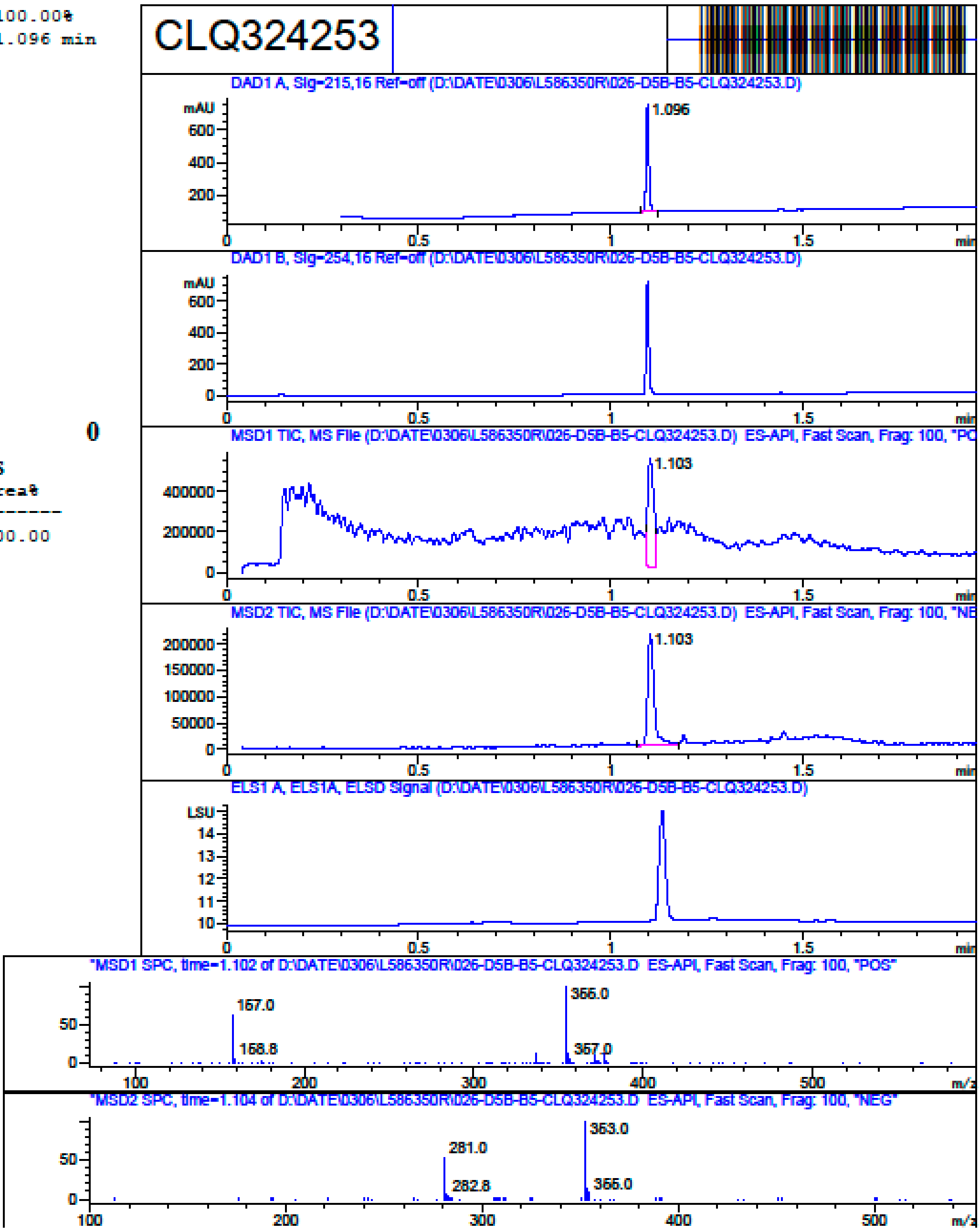

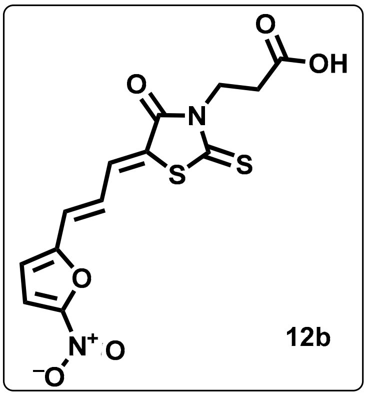

Molecular Weight: 354,35

Figure S50. LC-MS spectrum of compound **12b**

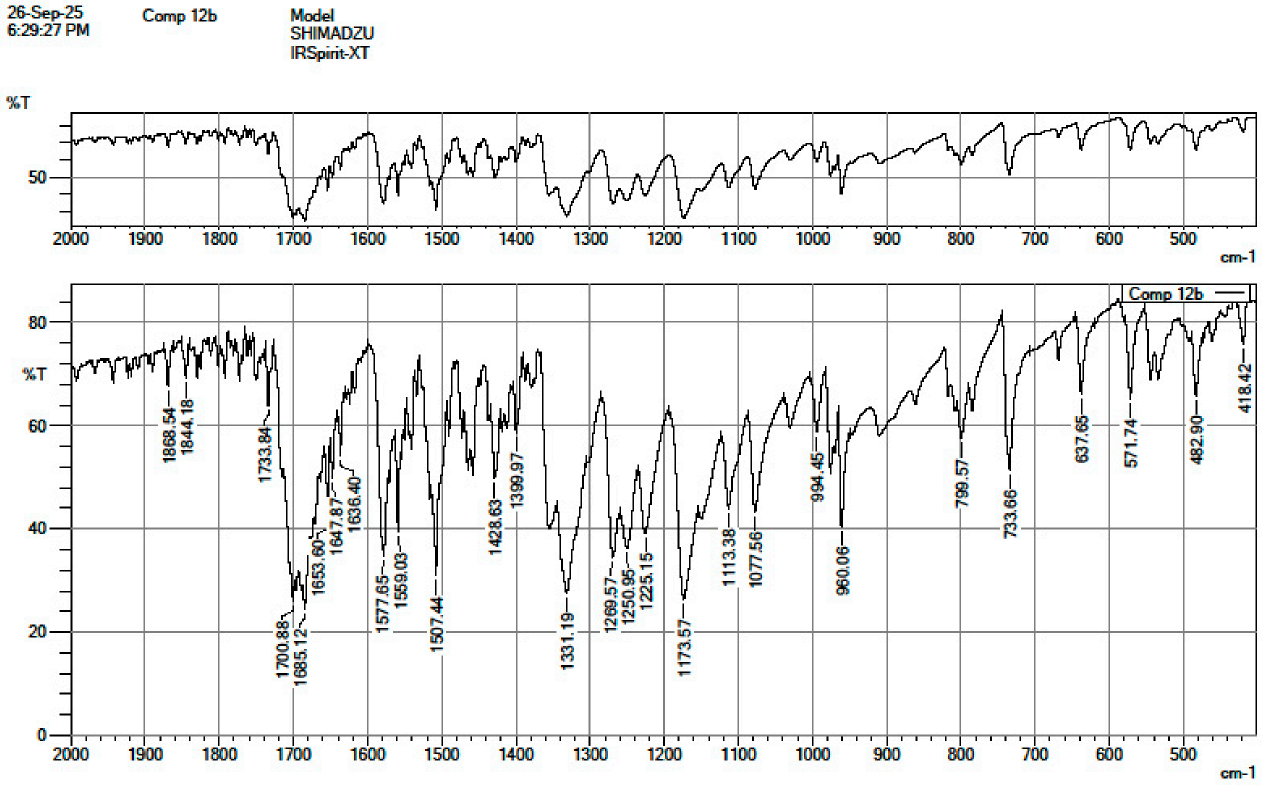

Figure S51. IR spectrum of compound **12b**

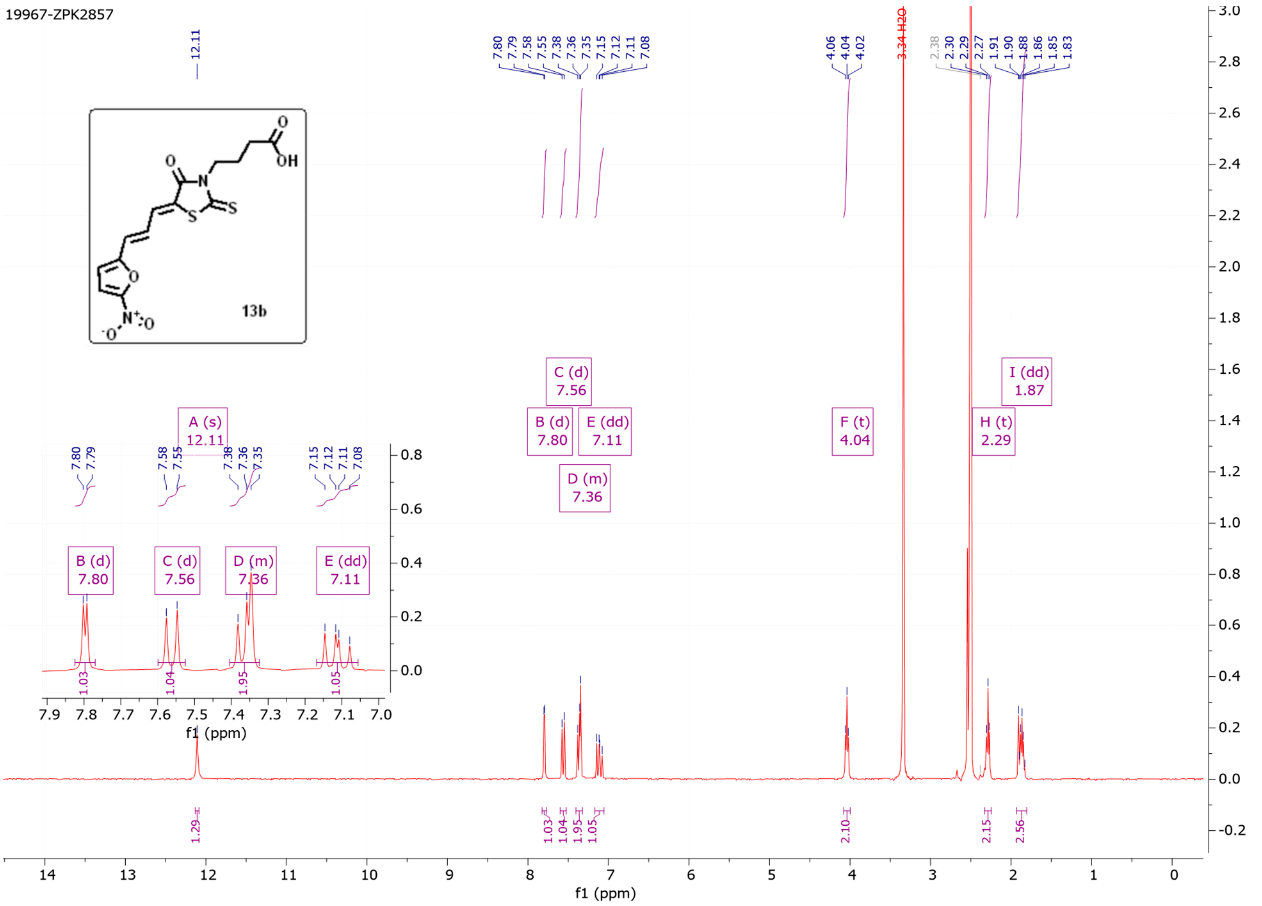

Figure S52. ^{1}H NMR spectrum of compound **13b**

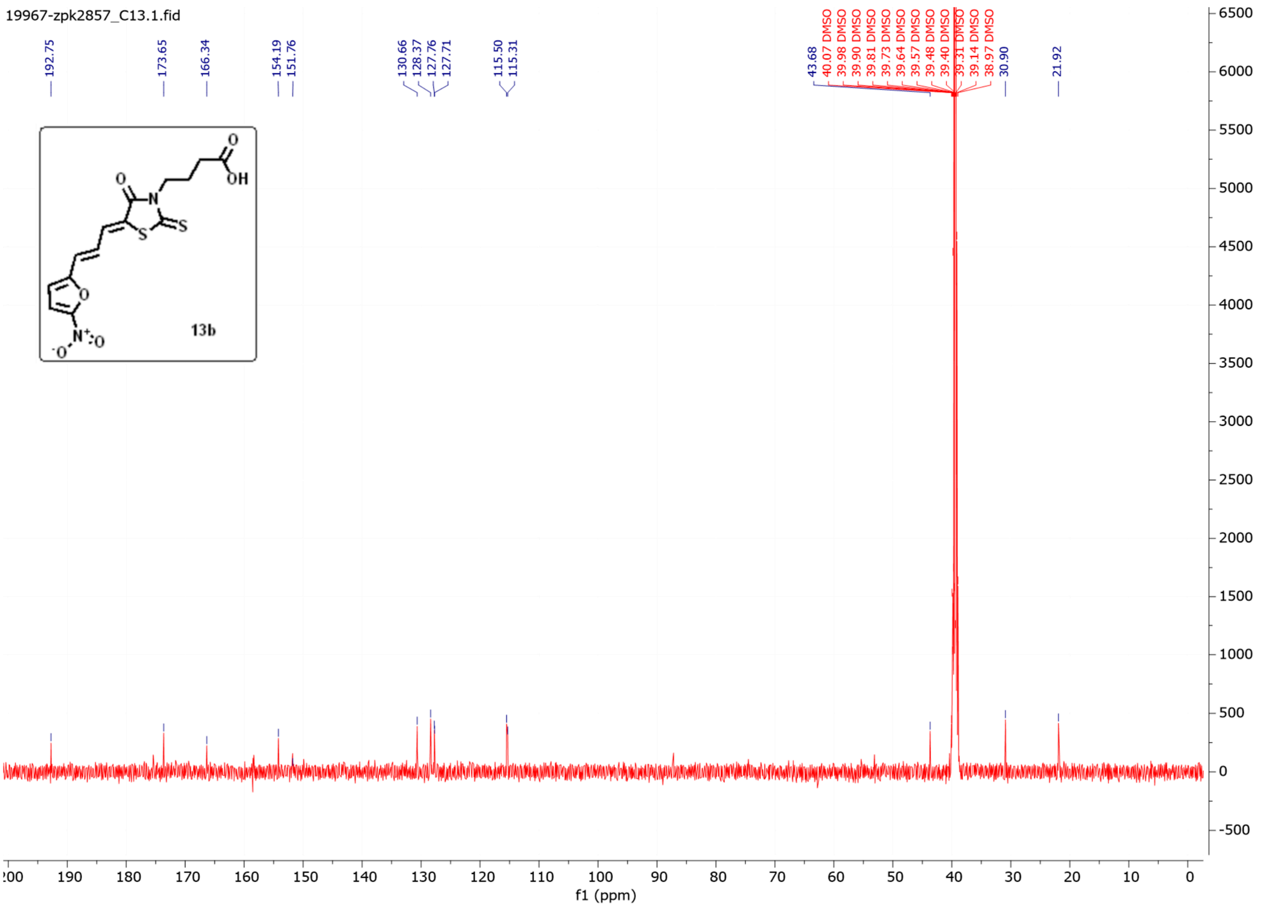

Figure S53. ^{13}C NMR spectrum of compound **13b**

#	RT	DAD1A	DAD1B	MSD1	MSD2	ELSD	MSD1 ions	MSD1 rt	MSD2 ions	MSD2 rt	Info
1	1.337	100.0%	100.0%	100.0%	100.0%	100.0%	369.0(89),351.0(11)	1.344	367.0(75),281.0(17),307.0(5)	1.345	

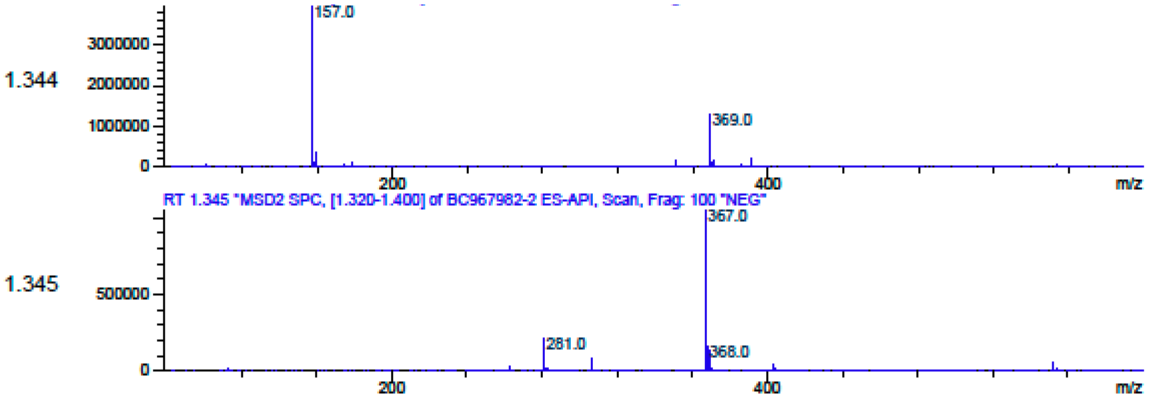

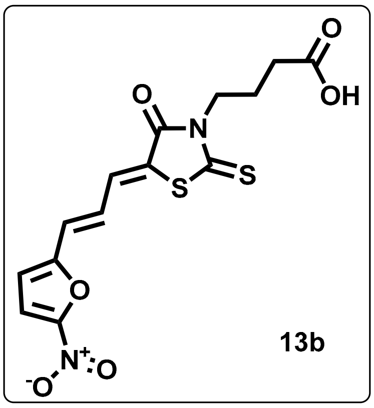

Molecular Weight: 368,38

Figure S54. LC-MS spectrum of compound **13b**

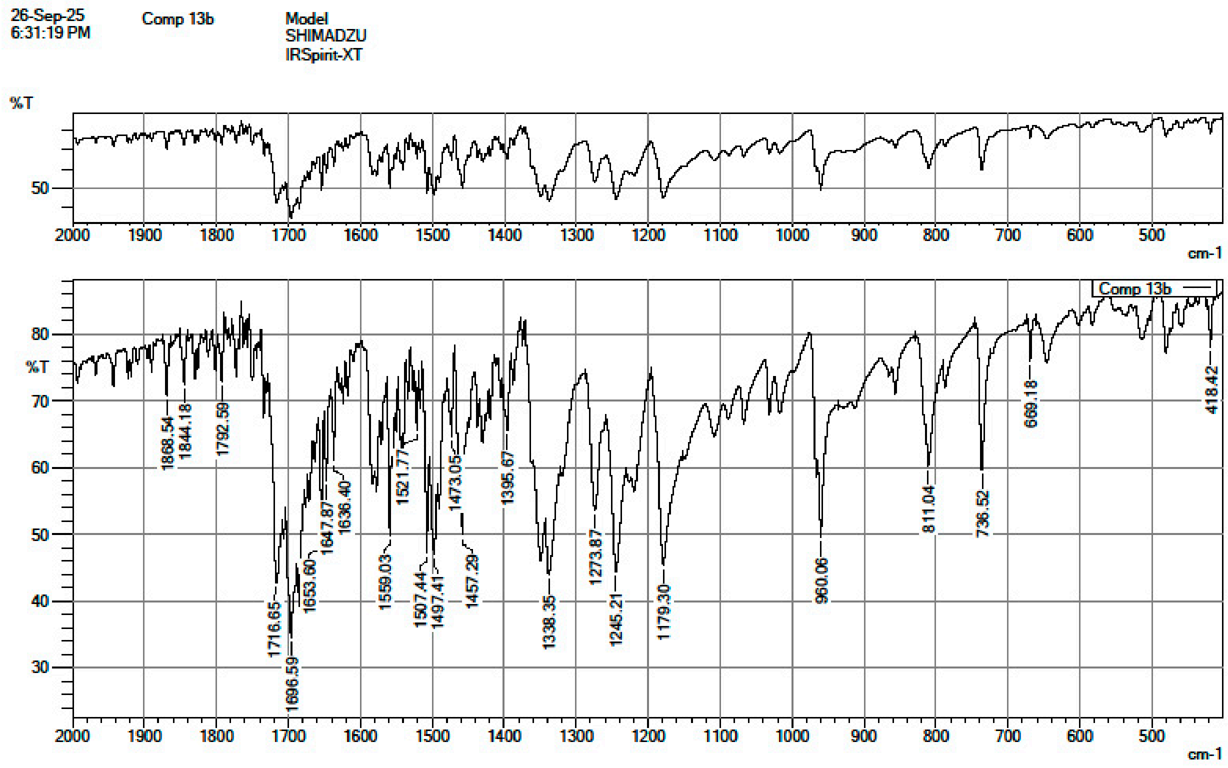

Figure S55. IR spectrum of compound **13b**

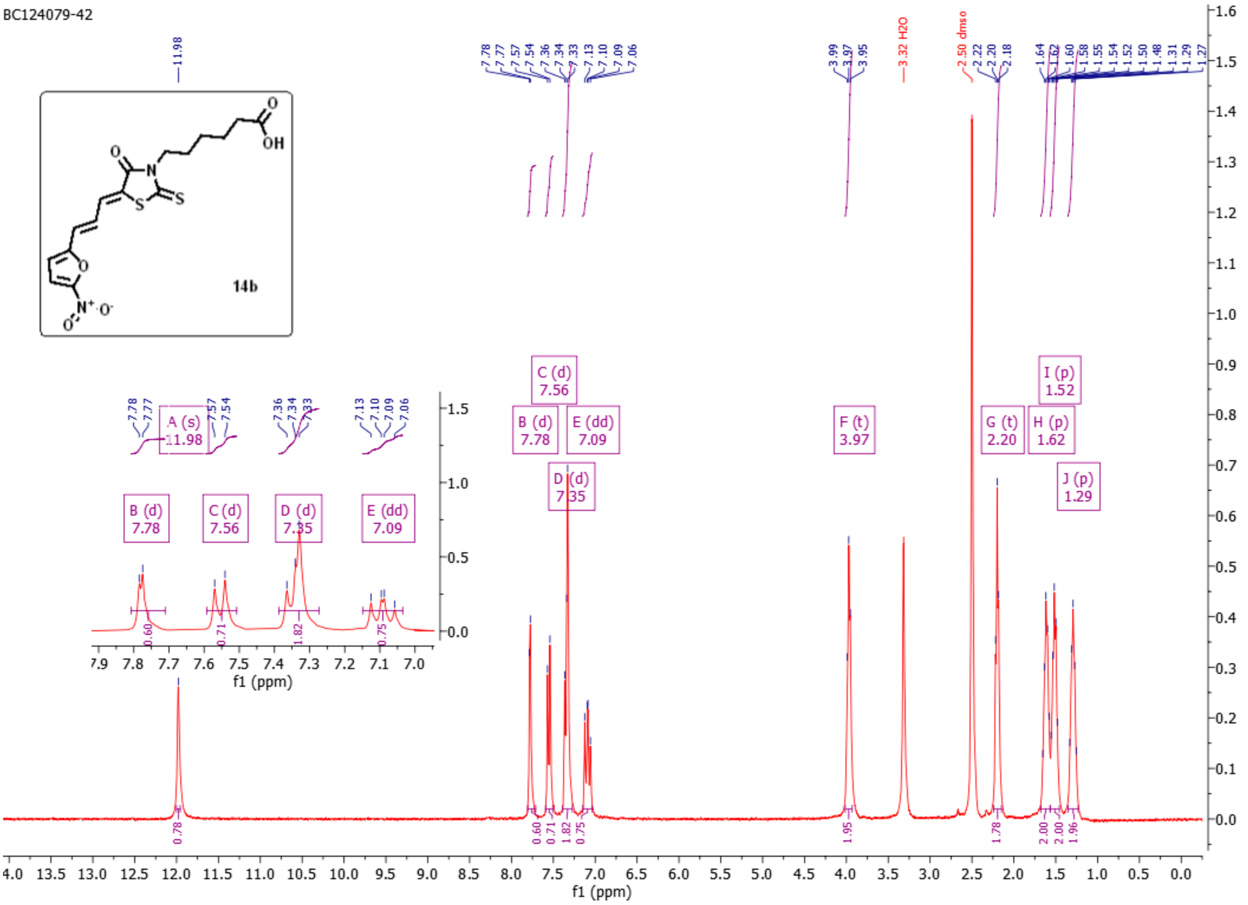

Figure S56. ^{1}H NMR spectrum of compound **14b**

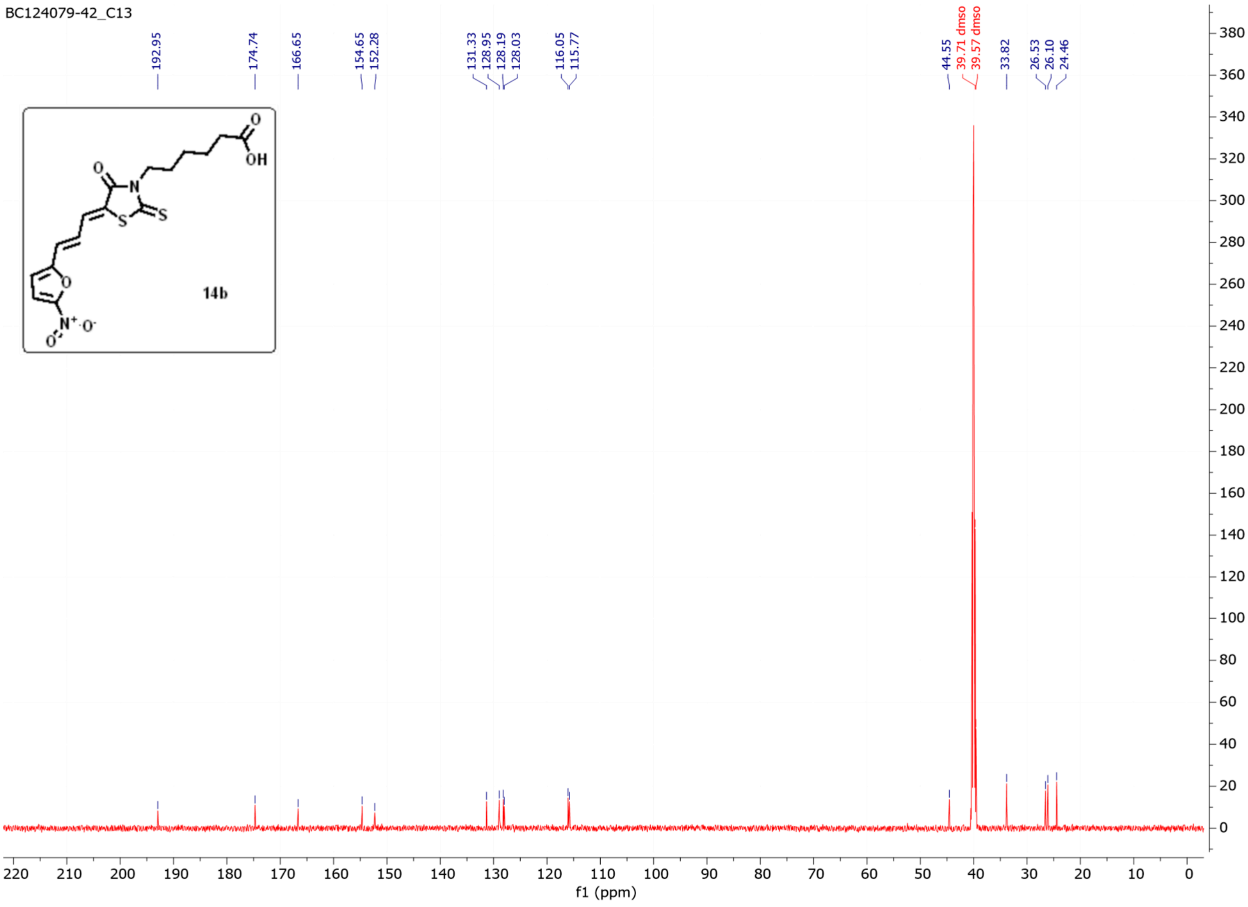

Figure S57. ^{13}C NMR spectrum of compound **14b**

#	RT	DAD1A	DAD1B	MSD1	MSD2	ELSD	MSD1 ions	MSD1 rt	MSD2 ions	MSD2 rt	Info
1	1.437	96.8%	96.7%	83.3%	98.3%	100.0%	397.0(79),379.0(21)	1.444	395.0(90),353.0(5),431.0(3)	1.446	
2	1.442	---	---	14.2%	1.7%	---	419.0(100)	1.449	348.0(100)	1.449	
3	1.470	3.2%	3.3%	2.5%	---	---	592.0(100)	1.479	---	---	

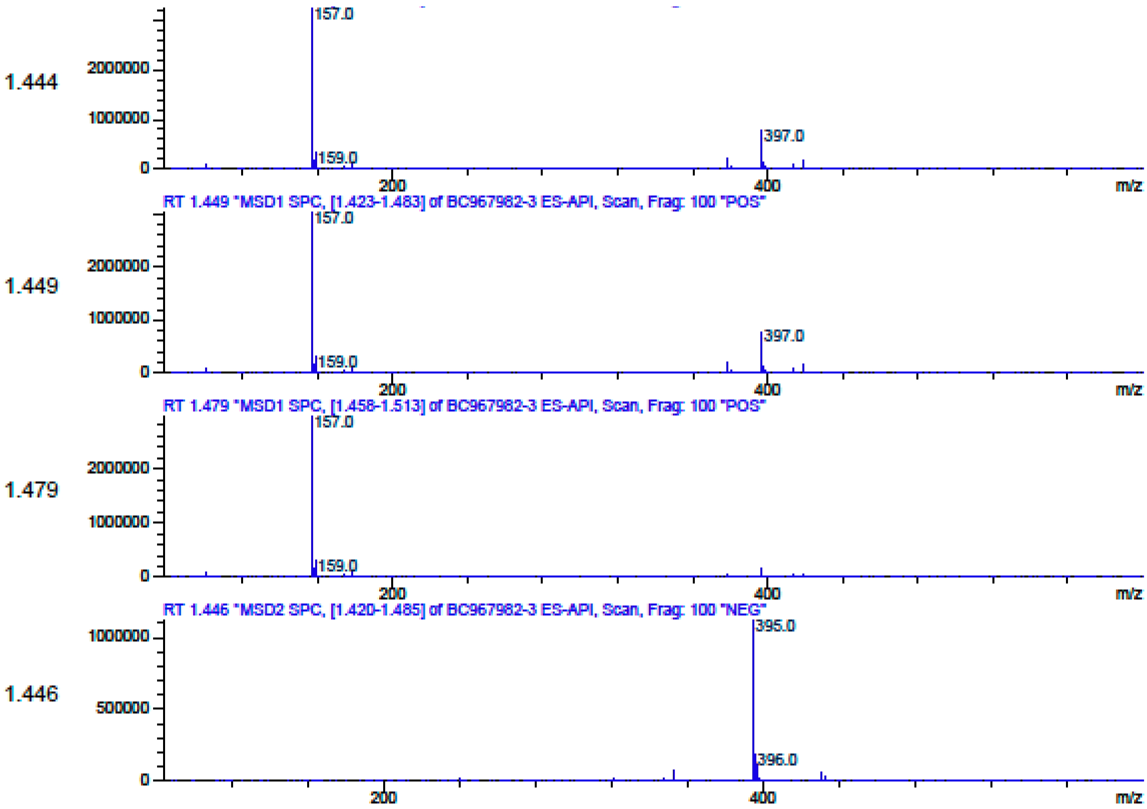

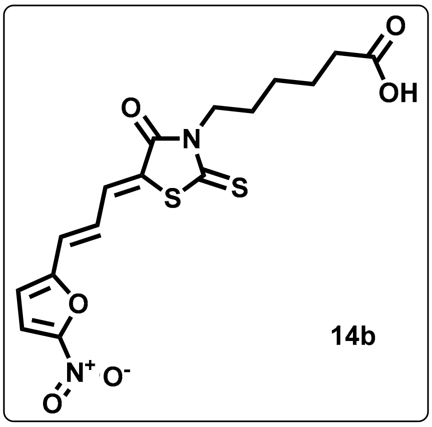

14b

Molecular Weight: 396,43

Figure S58. LC-MS spectrum of compound **14b**

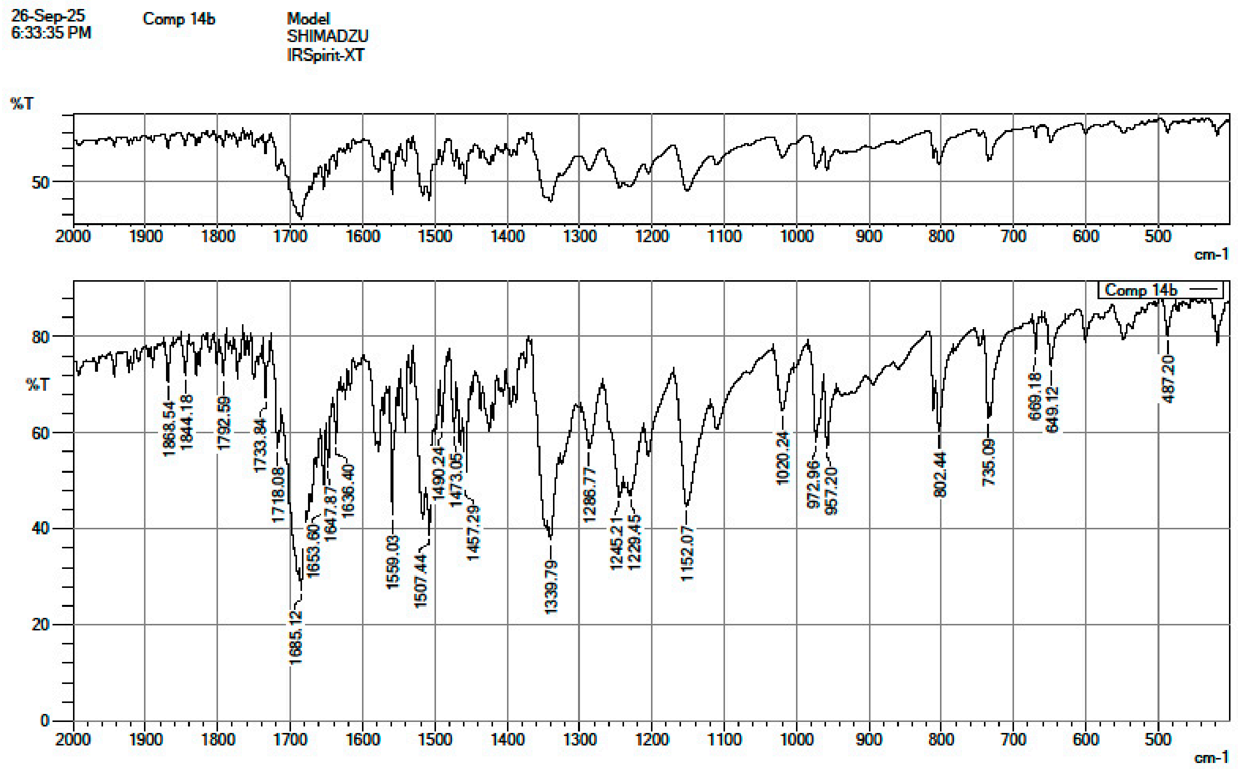

Figure S59. IR spectrum of compound **14b**

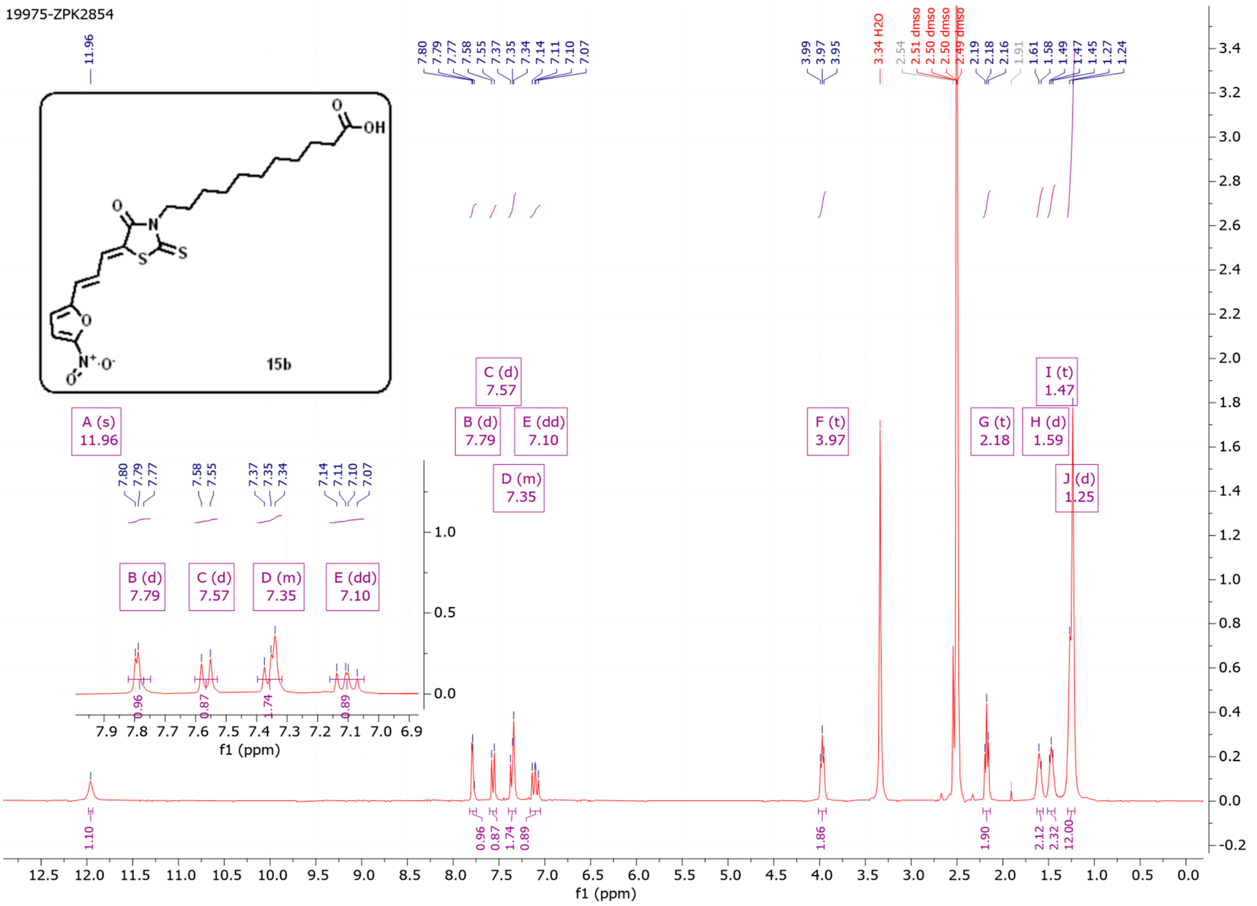

Figure S60. ^{1}H NMR spectrum of compound **15b**

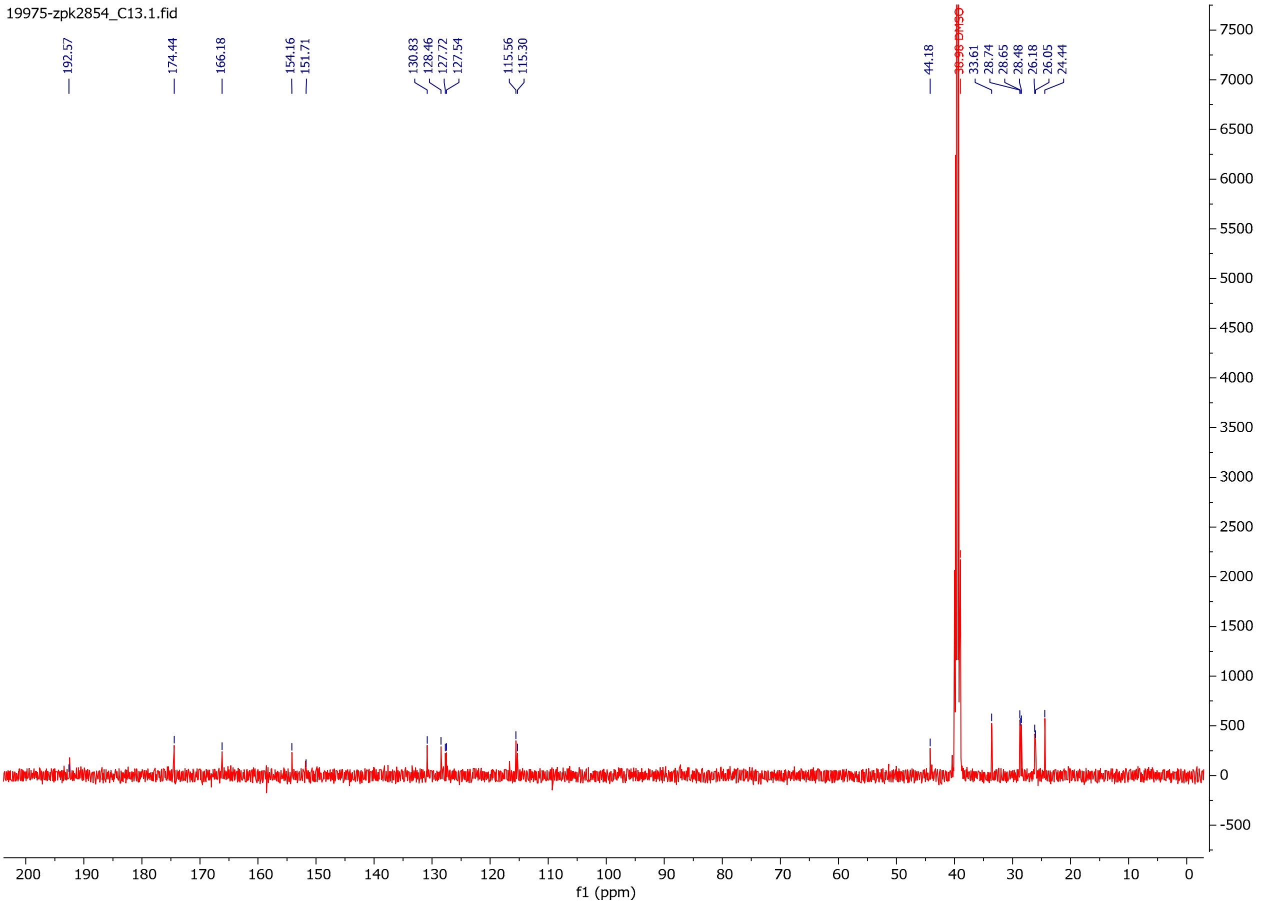

Figure S61. ^{13}C NMR spectrum of compound **15b**

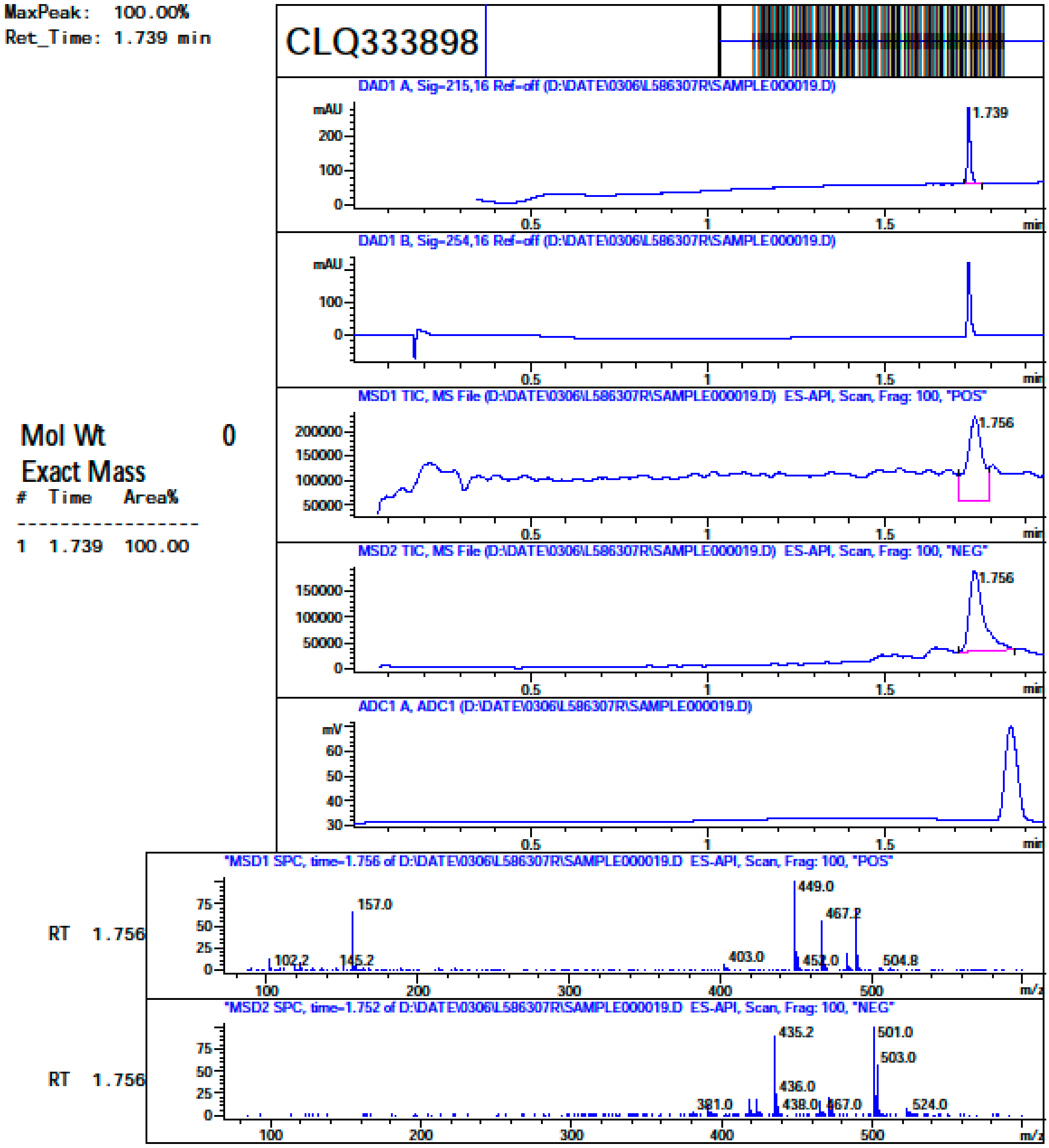

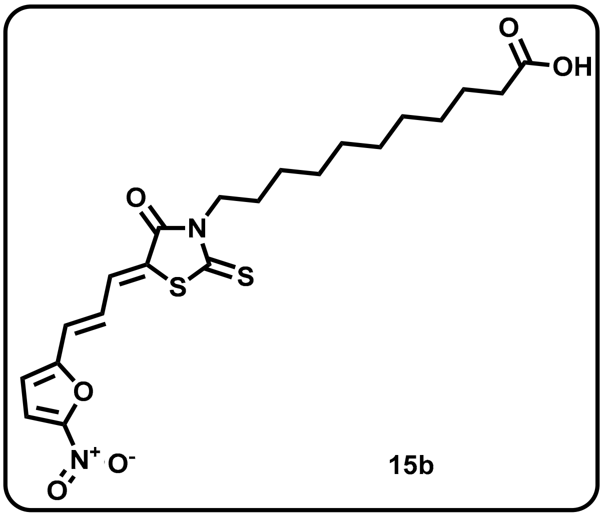

Molecular Weight: 466,57

Figure S62. LC-MS spectrum of compound **15b**

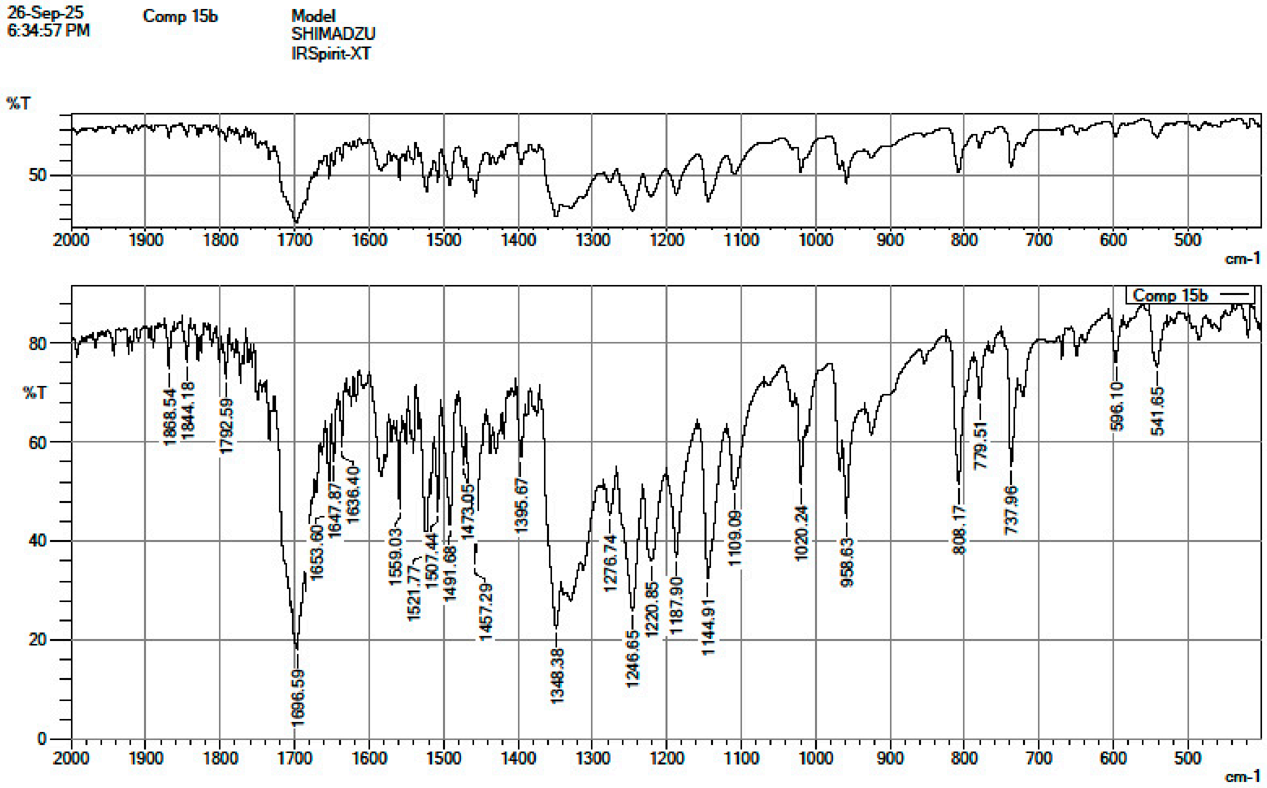

Figure S63. IR spectrum of compound **15b**

Table S1. NCI-60 anticancer screening data of compounds **4-6b**, **9b** and **12-14b** at 10 μM concentration on numerous cancer cell lines.

Comp. NSC	Mean growth, %	Range of growth, %	Sensitive cell line growth GP, % (cancer line/type)[a]	Positive cytostatic effect[b]	Positive cytotoxic effect[c]
**4b**	36.52	-98.90 to 45.89	-99.90 (SK-MEL-5 / Melanoma)<br>-95.04 (SN12C/ Renal Cancer)	4/55	51/55
**5b**	-73.80	-99.69 to 2.33	-95.90 (HCC-2998 / Colon Cancer)<br>-98.47 (SF-539 / CNS Cancer)<br>-97.77 (SNB-19 / CNS Cancer)<br>-95.39 (SK-MEL-28 / Melanoma)<br>-99.69 (SK-MEL-5 / Melanoma)<br>-95.93 (OVCAR-3 /Ovarian Cancer)<br>-99.54 (ACHN / Renal Cancer)<br>-97.14 (CAKI-1 / Renal Cancer)<br>-98.94 (DU-145 / Prostate Cancer)<br>-98.16 (BT-549 / Breast Cancer)	2/55	53/55
**6b**	-48.79	-99.30 to 46.65	-99.30 (SK-MEL-5 / Melanoma)	6/55	49/55
**9b**	26.29	-88.21 to 175.37	-88.21 (NCI-H522 / Non-Small Cell Lung Cancer)	28/55	10/55
**12b**	-62.05	-99.00 to 96.94	-95.81 (HCC-2998 / Colon Cancer)<br>-96.95 (SF-539 / CNS Cancer)<br>-95.70 (LOX IMVI / Melanoma)<br>-97.62 (SK-MEL-5 / Melanoma)<br>-97.43 (UACC-62 / Melanoma)<br>-96.70 (OVCAR-3 /Ovarian Cancer)<br>-96.94 (A498 / Renal Cancer)<br>-99.00 (ACHN / Renal Cancer)<br>-95.84 (SN12C/ Renal Cancer)	4/55	50/55
**13b**	-76.34	-100.00 to 5.30	-94.79 (HOP-62 / Non-Small Cell Lung Cancer)<br>-93.85 (NCI-H23 / Non-Small Cell Lung Cancer)<br>-95.27 (HCC-2998 / Colon Cancer)<br>-98.91 (SF-539 / CNS Cancer)<br>-96.10 (SNB-19 / CNS Cancer)<br>-96.12 (LOX IMVI / Melanoma)<br>-98.67 (SK-MEL-5 / Melanoma)<br>-95.10 (UACC-62 / Melanoma)<br>-97.74 (OVCAR-4 /Ovarian Cancer)<br>-97.64 (OVCAR-5 /Ovarian Cancer)<br>-100.00 (ACHN / Renal Cancer)<br>-96.66 (CAKI-1 / Renal Cancer)<br>-97.90 (RXF 393 / Renal Cancer)<br>-96.81 (SN12C/ Renal Cancer)<br>-97.38 (TK-10/ Renal Cancer)<br>-99.43 (DU-145 / Prostate Cancer)<br>-95.61 (MDA-MB-231/ATCC / Breast Cancer)<br>-98.54 (BT-549 / Breast Cancer)	2/55	53/55
**14b**	-67.89	-99.76 to 114.53	-95.72 (HCC-2998 / Colon Cancer)<br>-98.05 (SF-539 / CNS Cancer)<br>-95.58 (LOX IMVI / Melanoma)<br>-96.88 (UACC-62 / Melanoma)<br>-95.33 (OVCAR-3 /Ovarian Cancer)<br>-97.80 (OVCAR-4 /Ovarian Cancer)<br>-96.01 (OVCAR-5 /Ovarian Cancer)<br>-99.76 (ACHN / Renal Cancer)<br>-96.43 (CAKI-1 / Renal Cancer)<br>-95.04 (TK-10/ Renal Cancer)<br>-98.04 (DU-145 / Prostate Cancer)<br>-99.86 (MDA-MB-231/ATCC / Breast Cancer)<br>-97.16 (BT-549 / Breast Cancer)	4/55	50/55

[a] The most sensitive cell lines (GP < -95.00%); for **9b** - cell line with the greatest GI.
[b] Ratio between number of cell lines with GP from 0 to 50 and total number of cell lines.

[c] Ratio between number of cell lines with GP of <0 and total number of cell lines.

Table S2. NCI-60 assessmnet of GI_{50}/TGI/LC_{50} for compounds **4-6b**, **9b** and **12-14b** effects on individual tumor cell lines.

Cell lines/ comp.	**4b**	**5b**	**6b**	**9b**	**12b**	**13b**	**14b**
	GI_{50}/TGI/LC_{50}, μM	GI_{50}/TGI/LC_{50}, μM	GI_{50}/TGI/LC_{50}, μM	GI_{50}/TGI/LC_{50}, μM	GI_{50}/TGI/LC_{50}, μM	GI_{50}/TGI/LC_{50}, μM	GI_{50}/TGI/LC_{50}, μM
**Leukemia**							
CCRF-CEM	2.27/7.26/>100	0.36/1.81/>100	3.10/10.2/>100	3.12/13.3/>100	0.25/0.87/-	0.19/0.82/-<br>0.12/0.78/>100	0.18/0.57/4.49<br>0.27/0.96/>100
HL-60(TB)	2.17/6.43/>100	1.20/3.87/>100	2.68/10.1/>100	14.8/42.2/>100	1.28/3.30/-	0.60/2.51/-<br>0.29/1.09/>100	1.11/2.81/7.10<br>0.75/3.32/>100
K-562	2.77/14.5/>100	0.45/2.86/>100	2.60/10.5/>100	3.45/19.3/>100	0.34/2.73/>100	0.16/>100/>100<br>0.09/>100/>100	0.20/0.79/>100<br>0.28/>100/>100
MOLT-4	3.30/14.4/>100	2.34/10.7/>100	3.28/13.5/90.2	12.1/39.9/>100	0.58/2.56/>100	0.41/1.86/8.99<br>0.29/1.16/-	0.37/1.50/7.28<br>0.31/1.21/>100
RPMI-8226	1.79/5.11/>100	0.35/1.49/>100	1.78/6.09/>100	3.19/12.7/>100	1.23/4.48/>100	0.36/1.58/>100	0.33/1.32/-
SR	1.86/8.87/>100	0.32/1.89/>100	1.98/8.66/>100	3.15/18.0/>100	0.30/1.15/-	0.27/1.05/-<br>>100/>100/>100	0.27/0.96/-
**MG_MID**	2.36/9.43/>100	0.84/3.77/>100	2.57/9.84/98.4	6.64/24.2/>100	0.66/2.52/>100	9.34/28.3/87.0	0.41/11.35/64.9
**SI**	0.92/0.62/0.28	1.67/0.94/0.22	0.89/0.64/0.29	1.36/1.00/0.63	2.47/1.86/0.20	0.17/0.17/0.22	2.68/0.31/0.28
**Non-small cell lung cancer**							
A549/ATCC	2.22/5.40/23.1	1.86/3.83/7.89	2.33/5.86/52.4	16.8/47.0/>100	3.21/12.4/44.5	1.36/4.91/29.2<br>0.78/13.6/>100	1.62/3.25/6.53<br>2.38/11.6/>100
EKVX	2.53/7.77/27.7	2.01/4.80/13.9	2.26/5.74/19.7	14.3/28.1/55.1	2.69/9.26/31.1	1.83/4.24/9.84<br>1.05/2.52/6.05	1.74/3.48/6.94<br>1.68/3.55/7.52
HOP-62	2.23/5.43/42.6	1.35/3.08/7.03	2.25/5.20/31.3	13.1/44.5/>100	1.61/3.57/7.91	1.08/2.67/6.60<br>0.48/1.90/5.65	1.44/3.05/6.48<br>1.45/3.01/6.28
HOP-92	2.63/7.69/31.9	2.13/4.17/8.15	3.57/9.85/42.2	13.4/27.5/56.2	1.83/3.50/6.72	1.67/3.25/6.33<br>0.28/0.88/4.24	1.74/3.34/6.41<br>1.05/2.48/5.84
NCI-H266	2.62/7.83/78.9	2.40/7.65/88.8	2.22/5.84/76.1	12.2/33.1/89.7	2.83/12.5/79.7	2.79/11.9/76.1<br>1.65/9.78/>100	2.21/7.06/90.2<br>1.96/7.95/96.3
NCI-H23	1.97/4.11/8.57	1.75/3.41/6.63	1.85/3.51/6.64	14.4/31.0/66.9	1.69/3.37/6.70	1.53/3.09/6.24<br>0.53/2.04/5.70	1.53/3.10/6.29<br>1.23/2.69/5.89
NCI-H322M	3.72/14.2/38.1	1.82/3.88/8.29	3.84/15.0/43.9	13.4/26.6/52.5	7.06/19.8/44.7	2.52/8.09/28.3<br>1.97/5.50/22.8	1.68/3.39/6.84<br>1.82/3.55/6.92
NCI-H460	1.70/3.25/6.23	1.76/4.93/34.5	1.84/3.61/69.6	11.6/29.2/73.6	1.33/2.93/6.46	0.63/1.99/4.94<br>0.33/1.39/-	1.00/2.36/5.54<br>0.63/2.31/-
NCI-H522	1.70/3.32/6.48	1.12/2.53/5.69	1.74/3.43/6.78	4.09/19.1/64.6	1.10/2.46/5.52	1.15/2.57/5.71<br>0.17/0.34/0.69	1.28/2.70/5.66<br>0.18/0.38/0.82
**MG_MID**	2.37/6.56/29.3	1.80/4.25/20.1	2.43/6.45/38.7	11.1/31.8/73.2	2.59/7.75/25.9	1.21/4.48/24.6	1.48/3.85/21.8
**SI**	0.92/0.89/0.95	0.78/0.83/1.08	0.95/0.97/0.74	0.81/0.76/0.86	0.65/0.61/0.82	1.33/1.09/0.76	0.74/0.92/0.83
**Colon cancer**							
COLO 205	1.95/3.60/6.66	1.33/2.79/5.83	2.02/3.66/6.65	6.65/22.2/60.4	3.21/12.4/44.5	1.33/2.79/5.85<br>0.22/0.46/0.96	1.56/3.08/6.05<br>0.97/2.35/5.61
HCC -2998	1.83/3.33/6.07	1.72/3.22/6.04	1.83/3.34/6.07	15.6/29.9/57.5	2.69/9.26/31.1	1.77/3.38/6.46<br>0.44/1.65/4.21	1.78/3.32/6.19<br>1.60/2.98/5.53
HCT -116	1.82/3.90/-	0.32/1.13/-	1.73/4.05/-	2.68/8.14/>100	1.61/3.57/7.91	0.31/1.15/>100<br>0.17/0.64/3.79	0.31/1.05/4.96<br>0.20/0.42/0.91
HCT -15	1.65/3.39/6.97	0.73/2.18/5.32	1.71/3.58/7.47	7.73/22.9/59.0	1.83/3.50/6.72	0.18/0.52/2.48<br>0.11/0.33/0.97	0.19/0.51/2.05<br>0.16/0.35/0.80
HT29	1.95/3.81/7.45	1.37/2.90/6.14	1.98/3.71/6.95	5.02/22.4/94.5	2.83/12.5/79.7	0.45/1.72/5.44<br>0.26/0.64/-	1.19/2.71/6.19<br>1.13/3.39/>100.0
KM12	1.38/2.78/5.62	1.17/2.57/5.63	1.63/3.24/6.45	6.66/20.6/48.3	1.69/3.37/6.70	1.27/2.95/6.63<br>0.31/1.26/4.17	1.42/2.83/5.65<br>1.19/2.67/5.97
SW-620	1.55/3.08/6.12	0.59/2.55/13.4	1.80/3.38/6.36	2.94/9.66/43.9	7.06/19.8/44.7	0.24/0.54/1.72<br>0.28/0.83/-	0.23/0.57/2.20<br>0.24/0.61/-
**MG_MID**	1.73/3.41/6.48	1.03/2.48/7.06	1.81/3.57/6.66	6.75/19.4/66.2	1.33/2.93/6.46	0.52/1.35/11.9	0.87/1.92/11.7
**SI**	1.25/1.72/4.30	1.10/2.46/5.52	1.27/1.76/4.28	1.34/1.24/0.95	1.10/2.46/5.52	3.10/3.61/1.58	1.26/1.86/1.55
**CNS cancer**							
SF -268	2.04/4.45/9.72	1.61/3.57/7.92	2.15/5.24/16.8	10.8/23.7/52.2	1.44/3.18/7.02	1.27/2.84/6.37<br>0.63/2.89/22.8	1.26/2.71/5.82<br>1.32/3.63/9.99
SF -295	2.20/6.76/25.7	1.83/3.49/6.69	2.23/4.99/13.5	17.4/31.9/58.4	2.17/5.21/16.3	1.66/3.24/6.31<br>0.68/2.07/5.08	1.91/4.08/8.72<br>1.70/3.36/6.61
SF -539	2.12/4.77/12.2	1.44/2.91/5.88	1.89/4.17/9.17	12.0/24.7/50.9	1.66/3.65/8.01	1.56/2.92/5.47<br>0.20/0.43/0.93	1.57/2.93/5.45<br>0.27/0.80/2.85

SNB -19	2.01/4.12/8.45	1.64/3.01/5.53	2.10/4.88/13.5	13.5/26.3/51.4	2.30/6.37/22.9	1.65/3.16/6.05<br>0.63/2.10/5.50	1.76/3.62/7.43<br>1.45/3.11/6.67
SNB -75	1.53/3.27/6.99	0.68/1.98/4.89	1.77/3.77/8.06	2.11/7.39/27.9	1.10/3.67/14.1	0.32/1.37/5.49<br>0.38/1.99/8.73	0.35/1.43/4.03<br>1.20/2.61/5.68
U251	1.69/3.37/6.70	0.50/1.79/4.64	1.91/4.42/11.0	4.11/16.5/49.8	1.07/2.43/5.53	0.37/1.49/4.23<br>0.19/0.54/3.16	0.57/1.92/4.86<br>0.34/1.22/5.02
**MG_MID**	1.93/4.46/11.6	1.28/2.79/5.93	2.01/4.58/12.0	9.99/21.7/48.4	1.62/4.09/12.3	0.80/2.09/6.68	1.14/2.62/6.09
**SI**	1.12/1.31/2.41	1.09/1.27/3.68	1.14/1.42/2.38	0.90/1.11/1.31	1.00/1.15/1.73	2.01/2.33/2.81	0.96/1.37/2.97
**Melanoma**							
LOX IMVI	1.59/3.12/6.11	1.16/2.49/5.33	1.76/3.34/6.37	10.8/25.9/62.3	0.58/1.86/4.34	0.19/0.42/0.92<br>0.19/0.39/0.81	0.20/0.42/0.88<br>0.19/0.39/0.78
MALME -3M	1.80/3.40/6.40	1.32/2.73/5.64	2.21/4.47/9.02	5.40/18.7/48.8	0.50/1.66/4.25	0.24/0.60/2.18<br>0.15/0.35/0.83	0.28/0.76/2.85<br>0.24/0.72/2.72
M14	1.83/3.77/7.74	1.60/3.21/6.44	1.83/3.75/7.66	12.6/27.0/58.0	1.48/3.05/6.31	1.31/3.16/7.62<br>0.23/0.59/2.79	1.09/2.46/5.56<br>0.26/0.75/3.23
MDA-MB-435	1.62/3.31/6.77	0.85/2.17/5.01	1.81/3.95/6.61	3.04/13.0/40.6	1.16/2.48/5.32	0.44/1.69/4.52<br>0.20/0.43/0.94	0.42/1.69/4.47<br>0.28/0.75/2.74
SK-MEL-2	1.53/3.00/5.89	1.52/3.03/6.02	1.70/3.22/6.12	5.64/19.7/49.2	1.44/2.86/5.68	1.58/3.11/6.12<br>0.29/0.91/4.20	1.45/2.95/6.01<br>0.62/2.33/6.87
SK-MEL-28	1.76/3.51/6.98	1.46/2.83/5.48	1.91/3.73/7.29	10.7/23.4/51.1	1.57/3.05/5.93	1.36/2.70/5.38<br>0.21/0.52/1.80	1.64/3.11/5.90<br>1.19/2.71/6.21
SK-MEL-5	1.47/2.79/5.31	1.03/2.21/4.74	1.45/2.77/5.29	3.38/13.7/38.8	1.03/2.24/4.87	0.61/1.97/4.61<br>1.21/2.68/5.96	0.60/1.98/4.60<br>1.54/3.14/6.38
UACC-257	1.96/4.82/14.8	1.57/3.27/6.80	1.93/4.31/9.62	13.2/27.2/56.1	1.81/4.08/9.21	1.56/3.15/6.36<br>0.43/1.73/4.66	1.60/3.16/6.27<br>1.40/3.08/6.81
UACC-62	1.50/2.98/5.90	0.78/2.16/4.90	1.38/2.78/5.61	2.69/11.1/35.3	0.65/2.00/4.16	0.31/1.45/3.89<br>0.18/0.44/1.22	0.37/1.59/4.08<br>0.25/0.78/2.82
**MG_MID**	1.67/3.41/7.32	1.25/2.68/5.59	1.78/3.59/7.07	7.49/20.0/48.9	1.14/2.60/5.56	0.59/1.46/3.60	0.76/1.82/4.40
**SI**	1.30/1.72/3.81	1.12/1.32/3.90	1.29/1.75/4.03	1.47/1.80/3.83	1.47/1.80/3.83	2.73/3.34/5.22	1.45/1.97/4.11
**Ovarian cancer**							
IGROV1	1.94/3.87/7.75	1.78/3.42/6.57	2.16/4.86/18.0	14.3/30.8/66.6	1.43/2.86/5.70	0.55/1.92/5.14<br>0.23/0.70/7.77	0.63/2.06/5.01<br>0.23/0.57/2.67
OVCAR-3	2.18/5.39/17.6	1.34/2.77/5.73	2.23/5.33/16.6	9.26/21.4/46.9	1.36/3.01/6.65	0.77/2.04/4.59<br>0.39/1.36/4.01	1.12/2.35/4.91<br>0.96/2.22/5.02
OVCAR-4	6.03/19.1/44.3	2.43/6.72/26.3	6.12/19.0/44.5	13.9/28.0/56.1	1.79/3.79/8.07	1.56/2.94/5.54<br>1.16/2.58/5.75	1.49/2.84/5.41<br>1.31/2.69/-
OVCAR-5	1.89/4.32/9.85	2.13/3.83/6.89	2.08/4.62/10.8	12.7/25.8/52.6	1.99/3.70/6.88	1.85/3.32/5.95<br>0.72/2.02/4.69	1.85/3.31/5.93<br>1.56/2.97/5.67
OVCAR-8	1.98/4.64/13.0	1.49/4.78/25.1	2.12/4.86/13.8	11.1/25.5/58.5	1.84/7.11/28.2	0.46/2.25/9.54<br>0.27/1.24/25.8	0.57/2.12/6.43<br>0.30/1.02/9.71
NCI/ADR-RES	2.03/4.96/21.4	1.47/4.13/17.5	2.24/5.61/29.3	13.5/33.9/84.9	1.21/4.28/24.8	0.29/1.23/7.30<br>0.26/0.77/31.6	0.45/1.85/7.20<br>0.43/2.39/>100.<br>0
SK-OV-3	5.80/18.7/47.2	2.79/10.7/33.9	6.27/19.4/46.7	15.9/29.9/56.3	4.70/16.1/43.7	2.73/7.44/28.1<br>2.18/13.1/39.4	1.71/3.60/7.55<br>2.98/10.9/37.4
**MG_MID**	3.12/8.71/23.0	1.92/5.19/17.4	3.32/9.10/25.7	13.0/27.9/60.3	2.05/5.84/17.7	0.96/2.99/13.2	1.11/2.92/15.6
**SI**	0.70/0.67/1.21	0.73/0.68/1.25	0.69/0.69/1.11	0.69/0.86/1.05	0.82/0.80/1.20	1.68/1.63/1.42	0.99/1.23/1.16
**Renal Cancer**							
786-0	3.71/12.6/56.7	1.06/3.70/17.2	3.11/9.30/47.4	3.67/12.8/58.5	1.38/3.54/9.14	0.32/1.30/4.84<br>0.28/0.90/4.11	0.61/2.06/5.68<br>0.32/1.23/5.08
A498	2.24/7.21/26.7	2.69/4.81/8.62	3.03/8.28/31.6	20.8/36.5/64.0	14.3/27.4/52.5	4.13/11.5/34.7<br>3.32/8.16/29.8	8.13/20.8/46.5<br>10.2/22.8/51.2
ACHN	1.72/3.44/6.87	1.35/2.64/5.19	1.88/3.48/6.47	9.25/21.4/47.2	1.61/2.97/5.46	1.04/2.21/4.70<br>0.39/1.35/3.72	1.50/2.82/5.31<br>1.21/2.46/5.00
CAKI-1	1.75/3.70/7.84	1.17/2.46/5.17	1.67/3.29/6.48	6.27/19.7/44.9	1.42/2.74/5.30	0.67/1.97/4.59<br>0.77/2.04/4.57	0.83/2.09/4.64<br>1.40/2.72/5.26
RXF 393	1.89/3.40/6.11	1.74/3.27/6.12	1.69/3.20/6.06	4.01/14.6/39.4	1.58/3.02/5.83	1.35/2.74/5.55<br>0.18/0.53/2.04	1.65/3.10/5.83<br>0.31/1.11/3.76
SN12C	1.76/4.05/9.32	0.79/2.15/4.96	1.75/4.00/9.18	5.39/18.3/45.0	1.07/2.32/5.04	0.34/1.41/3.88<br>0.17/0.49/1.80	0.36/1.36/3.89<br>0.26/0.86/3.24
TK-10	3.27/8.16/27.6	2.24/4.03/7.23	3.13/8.15/28.3	16.1/30.0/55.9	2.02/3.81/7.19	1.73/3.18/5.84<br>0.71/2.03/4.77	1.88/3.32/5.84<br>1.85/3.47/6.33
UO-31	1.63/3.33/6.78	1.33/3.11/7.27	1.67/3.29/6.50	10.8/23.4/50.7	1.05/2.25/4.84	0.35/1.50/3.93<br>0.16/0.36/0.80	0.52/1.85/4.31<br>0.67/2.05/4.89
**MG_MID**	2.25/4.25/18.5	1.54/3.27/7.72	2.24/5.37/17.7	9.53/22.1/50.7	3.05/6.01/11.9	0.99/2.60/7.48	1.98/4.63/10.4
**SI**	0.96/1.38/1.51	0.91/1.08/3.00	1.03/1.17/1.61	0.95/1.09/1.25	0.55/0.78/1.79	1.63/1.88/2.51	0.56/0.77/1.74
**Prostate Cancer**							
PC-3	2.87/10.7/45.3	1.80/6.52/40.4	3.05/11.3/50.2	13.5/29.9/66.4	2.48/10.6/40.6	0.62/3.10/19.0<br>0.40/2.02/20.0	1.31/3.15/7.62<br>0.60/3.30/30.3

DU-145	2.42/6.42/22.8	1.43/2.82/5.56	3.35/10.2/32.9	11.5/23.7/48.8	2.61/10.6/33.3	1.09/2.32/4.92<br>1.15/2.83/7.01	1.52/2.87/5.44<br>1.59/3.18/6.35
**MG_MID**	2.65/8.56/34.1	1.62/4.67/23.0	3.29/10.8/41.6	12.5/26.8/57.6	2.55/10.6/37.0	0.82/2.57/12.7	1.26/3.13/12.4
**SI**	0.82/0.68/0.82	0.86/0.76/0.95	0.70/0.58/0.69	0.72/0.90/1.10	0.66/0.44/0.55	1.85/1.90/1.48	0.87/1.14/1.46
**Breast cancer**							
MCF7	1.60/3.70/8.54	0.43/1.83/5.60	1.64/4.11/12.1	3.67/16.5/59.3	0.46/2.10/6.46	0.35/1.97/7.03<br>0.20/1.58/6.61	0.39/1.82/6.04<br>0.44/1.99/6.87
MDA-MB-231/ATCC	2.21/4.29/8.33	1.56/3.03/5.90	2.27/4.45/8.73	10.8/24.2/54.2	1.70/3.24/6.15	1.12/2.41/5.19<br>0.21/0.49/1.32	1.36/2.72/5.44<br>0.28/0.64/2.23
HS 578T	1.87/10.1/>100	2.09/7.56/>100	3.38/27.4/>100	4.59/35.0/>100	1.45/7.93/>100	1.71/8.48/>100.0<br>0.33/3.22/>100.0	1.44/6.44/>100.0<br>0.79/7.76/>100.0
BT-549	1.67/3.37/6.77	0.75/2.14/5.13	1.49/3.12/6.55	2.46/7.81/35.7	1.35/2.88/6.13	1.12/2.52/5.65<br>0.19/0.43/0.96	0.77/2.15/5.07<br>0.24/0.67/3.03
T-47D	2.20/5.49/>100	1.12/3.24/-	1.75/4.03/-	11.1/39.4/>100	0.94/3.55/>100	0.34/2.01/>100.0<br>0.23/0.76/>100.0	0.64/3.12/>100.0<br>0.23/0.63/>100.0
MDA-MB-468	1.57/3.05/5.92	1.37/2.90/6.16	1.63/3.15/6.09	2.74/10.4/45.6	0.66/2.14/5.71	0.33/1.52/4.63<br>0.17/0.36/0.77	0.89/2.25/5.27<br>0.35/1.11/4.29
**MG_MID**	1.85/5.00/38.3	1.22/3.46/24.6	2.03/7.71/26.7	5.89/22.2/49.4	1.09/3.64/37.4	0.53/2.15/36.0	0.65/2.61/36.5
**SI**	1.17/1.17/0.73	1.15/1.02/0.89	1.13/1.99/4.68	1.53/1.09/1.28	1.54/1.29/3.73	3.04/2.30/0.52	1.69/1.37/0.50
**MG_MID**	**2.17/5.85/27.9**	**1.40/3.53/21.8**	**2.30/6.27/28.50**	**9.03/24.1/63.2**	**1.68/4.69/21.3**	**1.61/4.88/18.8**	**1.10/3.58/18.1**
